# Genome-Wide Identification of R2R3-MYB Genes and Expression Analyses During Abiotic Stress in *Gossypium raimondii*

**DOI:** 10.1038/srep22980

**Published:** 2016-03-24

**Authors:** Qiuling He, Don C. Jones, Wei Li, Fuliang Xie, Jun Ma, Runrun Sun, Qinglian Wang, Shuijin Zhu, Baohong Zhang

**Affiliations:** 1 Department of Biology, East Carolina University, Greenville, NC 27858, United States of America; 2 Department of Agronomy, Zhejiang Key Laboratory of Crop Germplasm, Zhejiang University, Hangzhou 310058, China; 3 Cotton Incorporated, Cary, NC 27513, USA; 4 Henan Institute of Science and Technology, Xinxiang, Henan 453003, China

## Abstract

The R2R3-MYB is one of the largest families of transcription factors, which have been implicated in multiple biological processes. There is great diversity in the number of *R2R3-MYB* genes in different plants. However, there is no report on genome-wide characterization of this gene family in cotton. In the present study, a total of 205 putative R2R3-MYB genes were identified in cotton D genome (*Gossypium raimondii*), that are much larger than that found in other cash crops with fully sequenced genomes. These GrMYBs were classified into 13 groups with the R2R3-MYB genes from *Arabidopsis* and rice. The amino acid motifs and phylogenetic tree were predicted and analyzed. The sequences of GrMYBs were distributed across 13 chromosomes at various densities. The results showed that the expansion of the *G. Raimondii* R2R3-MYB family was mainly attributable to whole genome duplication and segmental duplication. Moreover, the expression pattern of 52 selected GrMYBs and 46 GaMYBs were tested in roots and leaves under different abiotic stress conditions. The results revealed that the MYB genes in cotton were differentially expressed under salt and drought stress treatment. Our results will be useful for determining the precise role of the MYB genes during stress responses with crop improvement.

The MYB (myeloblastosis) transcription factor exists widely in eukaryotes and is one of the largest and most diverse families of transcription factors in the plant kingdom^1^,^2^. The first MYB gene, *v-MYB*, was identified from avian myeloblastosis virus (AMV)^3^. Subsequently, three v-MYB-related genes namely c-MYB, A-MYB and B-MYB were found in many vertebrates^4^. Homologous genes were also identified in fungi, insects and slime molds^5^. In comparison to animals, since the first plant MYB-encoding gene *COLORED1* (*C1*) was isolated from maize (*Zea mays*), a large number of MYB transcription factors have been explored in plants^6^,^7^. As compared to fungi and animals, the MYB superfamily is dramatically expanded in plant, with 100–250 MYB family members commonly found in an individual plant species^6^,^8^.

MYB family members have two structural regions. One is a highly conserved DNA-binding domain at the N-terminus, which referred to as the MYB domain^9^. However, MYB domains recently have also been discovered within the C-termini of MYB-proteins^10^. The MYB domain is well conserved among plants, yeast and animals. It is composed of approximately 53 amino acids residues in length that encode a helix–loop–helix structure^11^,^12^. The third helix is defined as a recognition helix that is in direct contact with DNA and intercalates in the major groove^13^,^14^. In contrast to MYB domain, the C-terminal region of MYB proteins is the flexible part of MYB transcription factors, and functions as transacting domain (TAD) responsible for the regulatory activity of the protein^13^,^15^,^16^. The MYB transcription factors in animal system consist of three sequential repeats (R1, R2, and R3)^2^. In plants, MYB-containing genes have greatly diversified, being divided into four classes, 1R-, R2R3-, 3R- and 4R- MYB proteins by the number and type of MYB domain repeats present^15^. It means 4R-MYB genes have four repeats (four R1/R2-type MYB repeats), the 3R-MYB genes (R1R2R3-MYB) have three consecutive repeats, the R2R3-MYB genes have two repeats and the MYB-related genes contain a single or partial MYB domain^2^,^6^. R2R3–MYB proteins comprise the largest group of MYB transcription factors in the MYB family and appear to be specific to plants and yeast^15^. For example, *Arabidopsis thaliana* was identified 198 MYB genes in genome sequence and 126 are two-repeat (R2R3) MYB proteins^9^. Rice (*Oryza sativa*) has over 110 predicted R2R3–MYB proteins and 244 R2R3-MYB proteins were identified in soybean^8^,^17^,^18^. The *Populus* genome contains 192 R2R3-MYB genes^19^. A genome-wide survey of the R2R3-MYB gene family in maize was performed and a total of 157 typical R2R3-MYB encoding genes in the maize genome were identified^20^. In addition, single-repeat MYB proteins have been identified in plants and animals in increasing numbers, and the majority of single-repeat MYB genes have been characterized in plants^21^.

Currently, in the plant transcription factor database, 8746 MYB and 6410 MYB-related sequences are available (http://planttfdb.cbi.edu.cn/). Furthermore, the varied function for a variety of MYB proteins have been investigated in numerous plant species by using both genetic and molecular analyses. MYB proteins play an important role in regulatory networks that involve in metabolic, cellular and developmental processes and response to biotic and abiotic stresses^6^,^15^,^22^. Of four MYB transcription factor groups in plants, the members of R2R3-MYB proteins are involved in an extensive diversification of functions^9^,^23^,^24^. They regulate different processes, including primary and secondary metabolism such as the regulation of various phytochemical biosynthesis pathways^25^,^26^; regulation of several developmental processes such as cell fate determination in root hairs^19^,^27^, secondary cell wall biosynthesis^28^,^29^,^30^, establishment of the axillary branch patterning^31^,^32^, leaf proximodistal axis and anther development^33^,^34^; and responses to environmental stresses^6^,^13^,^15^,^30^,^35^. For instance, *AtMYB5* from Arabidopsis regulated the outer seed coat differentiation while *AtMYB66* controlled root hair patterning^36^,^37^. *AmMYB308* and *AmMYB330* from *Antirrhinum majus* regulated lignin deposition in transgenic tobacco (*Nicotiana tabacum*) plants^38^. Two R2R3-MYB genes *EgMYB1* and *EgMYB2* have been characterized as repressors and activators of secondary cell wall formation in Eucalyptus (*Eucalyptus gunnii*)^39^. In *Arabidopsis thaliana*, three R2R3MYBs, *AtMYB37, AtMYB38* and *AtMYB84*, played a key role in controlling the pattern of lateral meristem initiation^40^; *AtMYB12* and *AtMYB111* controlled flavonol biosynthesis in *Arabidopsis thaliana* seedlings^41^. In cotton, *GhMYB25* regulated specialized outgrowths of epidermal cells, including cotton fibers^42^.

Several R2R3-MYB genes are involved in regulating responses to biotic and abiotic stresses such as: AtMYB2 was induced by dehydration and salt stress^43^; AtMYB62 is reported to be involved in phosphate starvation^44^; AtMYB96 acted through the ABA signaling mediate to drought stress^45^; AtMYB41 and AtMYB102 transcription factor genes were contributed to plant resistance against wounding and osmotic stress^46^,^47^. OsMYB3R-2 transgenic plants encodes a stress-responsive MYB transcription factor having a regulatory role in enhanced tolerance to freezing, dehydration and salt stress and decreased sensitivity to ABA in rice^48^,^49^. AmMYB1 transcription factor enhanced the tolerance to NaCl stress in transgenic tobacco^50^. GmMYB72, GmMYB96 and GmMYB117 were induced by ABA, salt, drought and/or cold stress treatment in soybean (*Glycine max*)^51^.

Cotton is one of the most important economic crops for its natural textile fiber in the world. It often encounters water stress such as drought or waterlog during its growth season^52^. *Gossypium raimondii,* a diploid cotton, belongs to D-genome species. The genome of *G. raimondii*, 761.4 Mb, is the largest in fiber crops that include cotton, flax (*Linum usitatissimum*) and mulberry (*Morus notabilis*). Full genome sequencing has been announced the date for completion by 2012^53^,^54^. Cotton genomic studies have boomed since the release of *G. raimondii* genome. Considering the multiple functions of MYB transcription factors, especially their important roles in response to abiotic stresses in plants, research was conducted concerning the evolution and expression properties of the R2R3-type MYB genes in cotton. In present study, we performed a genome‐wide identification of MYB transcription factors in the diploid cotton species *G. raimondii* and we focused on the whole genome-wide identification of R2R3-type MYB TFs in cotton. We carried out detailed studies of the phylogenetic relationship of the GrMYB proteins with other MYBs from different plant species, the genomic structure, the chromosome localization and other structural features. The expression pattern of 52 MYB-TF genes in response to abiotic stresses were also analyzed by quantitative real-time RT-PCR (RT-qPCR). Our study will serve as a foundation for future research into the functional roles of cotton MYB genes.

## Methods

### Database search for MYB proteins in *G. raimondii, Arabidopsis* and rice

Multiple database searches were performed to identify the MYB TFs in *G. raimondii*. The completed genome sequence of this species was downloaded from the Joint Genome Institute (JGI) Phytozome v9.1: *Gossypium raimondii* v2.1 website (http://www.phytozome.net/cotton.php). MYB transcription factor family genes of rice and *Arabidopsis* were obtained from MSU (http://rice.plantbiology.msu.edu/) and TAIR (http://www.Arabidopsis.org/), respectively^18^. The R2R3-MYB proteins from *Arabidopsis* and rice were used as query sequences. The gene identifiers were denoted to AtMYB genes in *Arabidopsis* and the locus id in rice to avoid confusion when multiple names are used for same gene.

To identify number of domains present in MYB protein, we used executed domain as queries to search against *G. raimondii* genome databases of JGI with BlastP and tBlastN program (default parameters). All hits considered as candidate sequences were executed by pfam database^55^ (http://pfam. sanger.ac.uk/) with both local and global search strategy. An E‐value cutoff of 1.0 was applied to keep significant matches, which were then further analyzed. On the basis of the results of BlastP and tBlastN searches in the *G. raimondii* genome database of JGI (using the predicted proteins of cotton MYB genes), we obtained information on cDNA sequences, genomic sequences, intron distribution patterns, phases, intron/exon boundaries and chromosome locations.

### Multiple sequence alignment and Phylogenetic analysis

Using ClustalW with default option^56^, we performed multiple sequence alignment on the obtained sequences of the MYB domains, and then manually adjusted the alignment by location of the corresponding amino acids in the R2R3-MYB motif^57^. We used MEGA v5.0^58^ to manually correct the alignment and recheck the results. The resulting amino acid sequence alignment were then used to guide the alignment of CDSs (coding domain sequence). In order to compare the evolutionary relationship of different plant MYB genes, we constructed a phylogenetic tree of the 205 R2R3-GrMYBs, 138 AtMYBs and 89 rice R2R3-MYB proteins, using the neighbor-joining (NJ) method of MEGA 5.0, with the following parameters: Poisson correction, pairwise deletion and bootstrap analysis with 1000 replicates for statistical reliability. To test the reliability of the result, we further performed maximum likelihood (ML) analysis of the R2R3-MYB gene family in cotton, using the software PhyML (http://atgc.lirmm.fr/phyml/)^59^, as well as a NJ analysis of whole GrMYB protein sequences by MEGA5.0, which based on the multiple sequence alignment of all predicted *G. raimondii* R2R3-MYB DNA-binding domains.

### Gene structure analysis and motif detection in cotton

The Intron‐Exon Structure pattern represents an independent guide to support subgroup designation of phylogenetic analysis^20^. Therefore, we analyzed the intron pattern (including the distribution, position, and phases) of the cotton R2R3-MYB gene family. The Intron/Exon organization for individual MYB genes were determined by aligning the cDNA sequences to their corresponding genomic DNA sequences that were used as the input for graphical display at the Gene Structure Display Server of Peking University, China (http://gsds.cbi.pku.edu.cn/).

In order to investigate the conserved motifs other than MYB repeats in the MYB protein sequences, we exploited the program of Multiple Em for Motif Elicitation (MEME; version 4.9.0) tool to analyze all of the full-length protein sequences of 205 GrMYBs^60^. The following parameter settings were used: distribution of motifs, zero or one per sequence; maximum number of motifs to find, 20; minimum width of motif, 6; maximum width of motif, 300 (to identify long R2R3 domains). Other options used the default values. Only motifs with an e-value of <1e^−20^ were retained for further analysis.

### Chromosomal location, tandem repeat and duplication

To map the locations of R2R3MYB genes in *G. raimondii*, the chromosomal distribution of MYBs were generated by MapInspect software according to their position given in the cotton sequence^61^. Two types of gene duplications are recognized: tandem duplication and segment duplication. Gene duplications were detected provided that the length of the aligned sequence covered >80% of the longer gene, and that the similarity of the aligned regions was >80%^62^,^63^. KaKs_calculator was then run on these MYB gene pairs to estimate their synonymous and non-synonymous rates of evolution^64^. To estimate time of the occurrence of duplication events, Ks value were translated into duplication time in millions of years ago^65^ based on a rate of 1 substitution per synonymous site per year. The duplication time (T) was calculated as T = Ks/2λ × 10^−6^ Mya (clock-like rates λ = 1.5 × 10^−8^ for *G. raimondii*)^54^.

### Plant materials, growth condition and stress treatments

For expression analysis of cotton MYB genes under stress, *G. raimondii* and *G. arboreum* were used to check the gene expression level in all experiments. The seed coats were removed and the seeds were sterilized by using 10% bleach followed by 70% ethanol, and then were cultivated on the full-strength Murashige and Skoog (MS) medium. The plants were grown in a chamber with a 16-h light/8-h dark cycle under a temperature 30 °C in light and 26 °C in dark. The drought and salt treatment was modified from the method described in our previous study^66^. At the appearance of the true leaf, the seedlings were subjected to the treatment by transferring them to a MS medium with additional 25 mM, 50 mM NaCl and 2.5%, 5% PEG. After two weeks of treatment, roots and leaves of seedlings were harvested, immediately frozen with liquid nitrogen and then stored at −80 °C until RNA isolation. Three biological replicates were conducted for each treated and untreated groups.

### Expression analyses of MYB genes under different stress treatment conditions

Fifty-two pairs of MYB gene specific primers from *G. raimondii* and 46 pairs of primers from orthologs in *G. arboreum* were used to study the expression of MYB transcription factors using qRT-PCR. Total RNAs of leaves and roots were isolated from both stress treated and control samples by using the mirVanaTM miRNA Isolation Kit (Ambion) with a minor modification according to our previous report^66^. The concentration and quality of total RNAs were detected by using a Nanodrop ND-1000 (Nanodrop technologies). Approximately 1 μg RNA was reverse transcribed using TaqMan^®^ MicroRNA Reverse Transcription Kit (Applied Biosystems) to generate cDNA. Real-time PCR was conducted in a 7300 Sequence Detection System (Applied Biosystems, USA) using SYBR Green ROXTm qPCR Mastermix (QIAGEN, USA). Reaction parameters for thermal cycling were: 95 °C for 10 min, followed by 45 cycles of 94 °C for 10 s, 60 °C for 1 min. The primer sequences are listed in Supplemental material Table S1. In each qRT-PCR experiment, each gene was run in triplicate with different cDNAs synthesized from three biological replicates of different tissues and treatments. *G. raimondii SAD1* and *TUA11* and *G. arboretum UBQ7* were used as reference genes to normalize all data. The amplification data were analyzed using software version 1.0 (Applied Biosystems, USA). The threshold cycle^67^ values of the triplicate PCRs were averaged. 2^−∆∆Ct^ was taken as the relative expression level for each sample. Statistical analysis was carried out using the data obtained from three separate cDNA sets of three independent biological samples. Results from different treatments were compared using one-way analysis of variance^30^. Statistical analyses were carried out using SPSS20.0.

## Results

### Genome-wide identification of the MYB family in *G. raimondii*

To identify MYB-related proteins in cotton genome, we implemented BLASTP searches of the complete genomes of *G. raimondii. Arabidopsis* MYB protein sequences were used as queries with the E-value cutoff set at 0.001. A total of 449 deduced amino acid sequences in cotton D genome, containing MYB or MYB-like repeats, were identified via BLAST searching of the cotton D genome sequence database. Subsequently, the redundant sequences of candidate MYBs were discarded from the data set according to their chromosome locations. All the putative MYBs were analyzed using simple modular architecture research tool (SMART) to confirm the MYB domain. The remaining cotton MYBs possessing incomplete ORFs were also excluded for further analysis. All cotton MYB proteins were manually inspected to ensure that the putative gene models contained 2 or 3 MYB repeats, and that the gene models mapped to unique loci in their respective genomes. After extensive analysis, a total of 205 genes were defined as *G. raimondii* R2R3-MYB proteins in cotton genome. To distinguish the remaining R2R3-MYBs, we provisionally named them GrMYB1 to GrMYB205, based on the order of the corresponding chromosome locations identified from the cotton genome browser (Supplemental material Table S2). The *G. raimondii* genome encodes more abundant R2R3-MYB family members (205) than either *Arabidopsis* (126)^6^,^9^ or rice (over 110)^17^,^18^; second in number only to soybean (*Glycine max*)^8^ in all currently known plant species. The identified GrMYB genes encode proteins ranging from 170 (GrMYB165) to 1026 (GrMYB160) amino acids in length with an average of 319 (Supplemental material Fig. S1).

**Figure 1:**
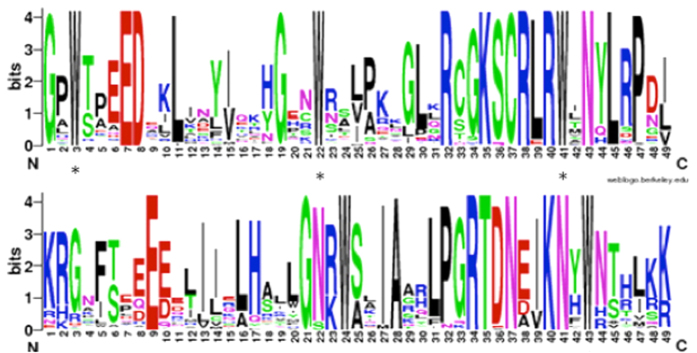
The R2 and R3 MYB repeats are highly conserved across all R2R3-MYB proteins in the *G. raimondii* genome. The sequence logos of the R2 (**a**) and R3 (**b**) MYB repeats are based on full-length alignments of all GrR2R3-MYB proteins. The bit score indicates the information content for each position in the sequence. Asterisks indicate the conserved tryptophan residues (Trp) in the MYB domain.

To investigate the feature of the homologous domain sequences, and the frequency of the most prevalent amino acids within the MYB domain, multiple alignment analysis was conducted to investigate the homologous R2R3-MYB domain in cotton. In general, the basic regions of the R2 and R3 repeats both had 49 basic residues. The R2, R3 repeats of cotton revealed highly-conserved sequences, furthermore 13 out of 49 and 10 out of 49 amino-acids were 100% conserved in all GrMYB proteins. Congruent with previous studies in other plant species, the R2 and R3 MYB repeats of the *G. raimondii* R2R3-MYB family contain characteristic amino acids, including a series of evenly distributed and highly conserved Trp residues that play a role in sequence-specific binding of DNA^9^,^19^,^68^ (Fig. 1).

### Phylogenetic analysis of the R2R3-MYB gene family among cotton, Arabidopsis, and rice

**Figure 2:**
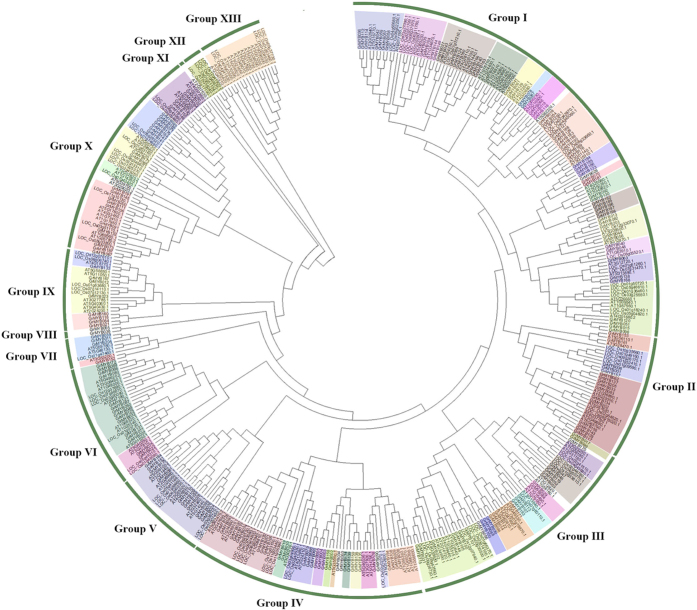
Phylogenetic analysis of the MYB transcription factors of Arabidopsis, rice, and cotton. Neighbor-joining phylogeny of 432 R2R3-Myb genes of 3 species, as determined by MEGA5.0. The colored shadow marks the subgroups of the MYBs. Numbers on branches are bootstrap proportions from 1000 replicates.

To examine the evolutionary relationships among the R2R3-MYB proteins of different plant species, all identified cotton R2R3-MYBs and all R2R3-MYBs from *Arabidopsis* and rice^18^,^69^ were subjected to a multiple sequence alignment. Based on sequence similarity and topology, then an unrooted phylogenetic tree was constructed by using MEGA 5.0 (Fig. 2). As shown in Fig. 2, the phylogenetic tree divided the R2R3-MYBs into 13 well-supported clades, named from I to XIII groups. With the exception of Group VIII, XI, XII and XIII, each group could be further divided into subgroups. Group I was composed the largest clade with 82 GrMYB genes. Phylogenetic comparative analysis of R2R3-MYBs in different plant species revealed considerable diversification and conservation of the MYB gene family in plant^20^. In present study, most subgroups were found to be share among the MYBs from cotton, rice and *Arabidopsis*, which suggested the existence of a common ancestor before the divergence of monocots and dicots. In contrast, several cotton MYBs were not clustered together with Arabidopsis and rice MYBs, suggesting that these MYB genes may be acquired in cotton after diverging from the last common ancestor or have been either lost in *Arabidopsis* and rice. In other cases, group XIII and some subgroups only include *Arabidopsis* and rice MYB proteins but no one was identified in cotton, indicating that these genes may have been lost in cotton during the evolutionary process. Several clades do not include any rice R2R3-MYB proteins but only members from cotton and *Arabidopsis*. This suggests that the presumed rice-specific loss of gene in these clades might have specialized roles that were either lost in rice or acquired in the dicotyledons lineages after divergence from the last common ancestor with monocotyledons. The birth and lose of species-specific MYBs results in functional divergence. Though the roles of most GrMYBs remain to be elucidated, it is likely that the R2R3-MYB genes with the conserved functions exhibited a tendency to cluster into the same subgroup and may have recent common evolutionary origins.

### Phylogenetic and conserved gene structure and protein motif analysis of R2R3-MYB gene family in cotton

**Figure 3:**
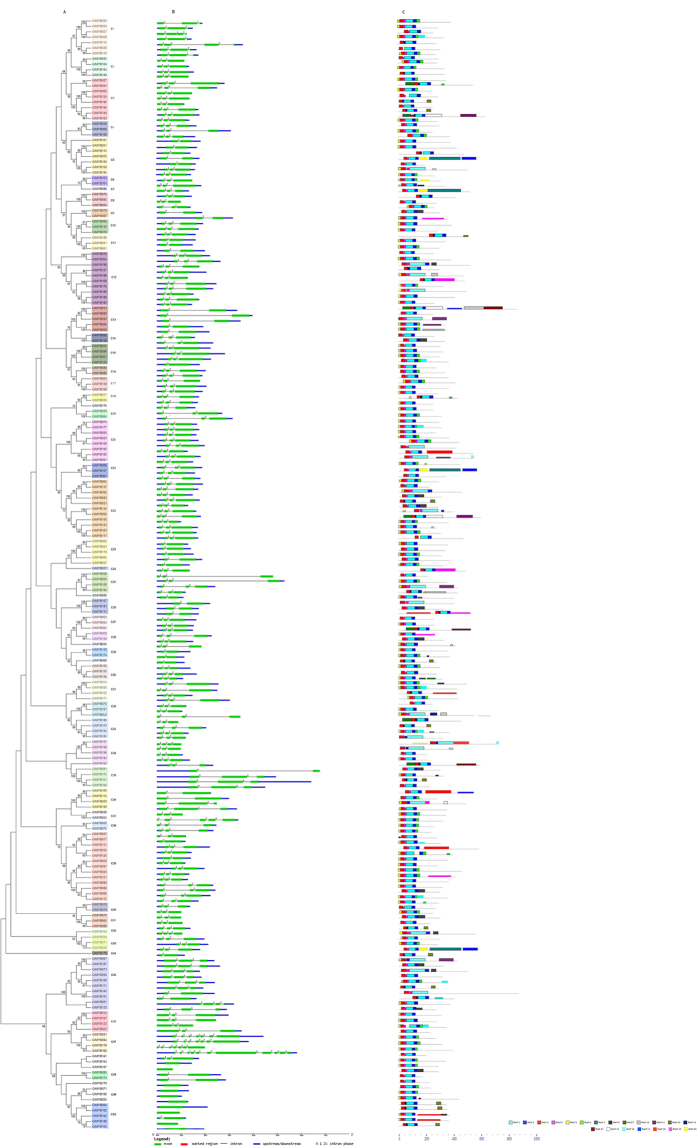
Phylogenetic relationships, gene structure and motif compositions of the *Gossypium raimondii* R2R3-MYB genes. (**A**) The phylogenetic tree was constructed with MEGA 5.0 using the Neighbor‐Joining (NJ) method with 1,000 bootstrap replicates based on a multiple alignment of 205 amino acid sequences of R2R3-MYB genes from *G. raimondii*. Bootstrap values higher than 50% are shown on the nodes. The 50 major subfamilies are indicated with C1 to C0 are marked with colorful backgrounds. (**B**) Exon/intron structures of R2R3-MYB genes from *G. raimondii*. The exons and introns are represented by green boxes and black lines, respectively. The scale bar represents 1.0 kb.(**C**) Protein motif. Schematic diagram of the conserved motifs in the R2R3-MYB proteins in cotton, which were elucidated using MEME. Each motif is represented by a number in the colored box. The black lines represent the non-conserved sequences. The scale bar represents 200 aa.

To better understand the similarity and diversity of gene structure and motif compositions among GrMYBs, we constructed a separate phylogenetic tree using NJ method in MEGA 5.0. The 205 typical members of the cotton R2R3-MYB gene family were subdivided into 50 subgroups (designated C1–C50), according to clades with at least 50% bootstrap support (Fig. 3A). Moreover, the tree topology of maximum likelihood (ML) analysis was essentially the same with the former unrooted phylogenetic tree as well, indicating that these phylogenetic trees were in good agreement. The low bootstrap support for the internal nodes of these trees was in accordance with phylogenetic analysis of MYBs in other organisms^20^. A total of 70 sister pairs of putative paralogous genes were found among the 205 GrMYB genes, and 34 of them had high bootstrap support (≥99%). In addition, C45–C50 was located at the outer portions of the phylogenetic tree, so we indicated that the genes in these six subgroups had relative ancient origins.

In order to gain further insight into the structural diversity of GrMYBs, the Intron‐Exon Structure pattern were investigated through *G. raimondii* MYBs. In the present study, a detailed illustration of the gene structures is shown in Fig. 3B. In general, R2R3-MYBs possessed at least 1 intron in the DNA-binding domain and up to 95% (195) of the 205 GrMYBs possessed 1–10 intron(s). In contrast, 10 of the 205 GrMYBs lacked introns. The majority of GrMYBs were typical splicing of 3 exons and 2 introns (145 of the 205 GrMYBs). However, GrMYB160 had the most 11 exons and 10 introns in its CDS. Remarkably, phylogenetic analysis of the GrMYB gene family showed that the genes in the same subgroups generally contained nearly the same intron pattern, with the position(s) of the intron(s) being fully conserved (Fig. 3B). For example, the subgroup C11 and C13 lacked introns 1 and subgroup C23 lacked intron 2. This finding constituted an independent criterion for testing the reliability of our phylogenetic analysis. Moreover, the number of introns in the MYB appeared to be limited, a majority of GrMYBs (92%) had no more than 2 introns, which is similar in *Arabidopsis*^70^. These results indicate that the intron patterns are not random, but highly conserved. Introns would be inserted or excised from the MYB coding region in a subfamily-specific manner, suggesting that the introns have been specifically inserted into plants and retained in the genome during the evolution^20^,^71^.

Conserved motifs in the 205 GrMYB proteins were identified using the MEME online tool (Fig. 3C). Twenty motifs were identified on the GrMYBs (Fig. S1). The motif lengths identified by MEME were between 11 and 164 amino acids. Most GrMYBs have motifs 1, 2, 3, 4 and 5. The number of the conserved motifs in each MYB varied between 3 and 9. This indicates that most members of a same subgroup shared 1 or more identical motifs outside the MYB DBD. The majority of close members in the phylogenetic tree exhibited common motif compositions, which suggested that there were functional similarities within the same subgroup.

### Chromosomal location and duplication events of cotton R2R3-MYB genes

**Figure 4:**
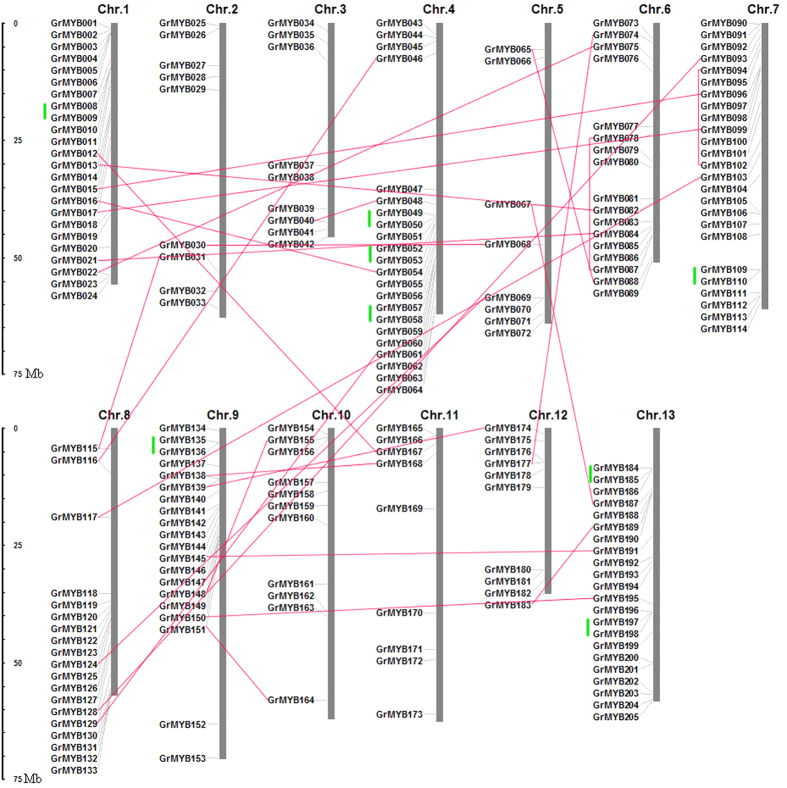
Chromosomal locations and region duplication for cotton R2R3-MYB genes. The chromosomal position of each GrMYB was mapped according to the *G.raimondii* genome. The chromosome number is indicated at the top of each chromosome. The scale is in mega bases (Mb). The segmental duplicated genes are indicated by red lines and the tandemly duplicated genes by green vertical lines.

To date, the information regarding expansion events of the R2R3-MYB gene family in cotton remain unclear. In order to determine the relationship between genetic divergence and gene duplication within the R2R3-MYB gene family in cotton, we investigated the chromosomal location of GrMYBs, based on the annotation of the *G. raimondii* genome. The result indicated that 205 R2R3-MYB genes were assigned to 13 chromosomes (Fig. 4). We found the distribution appeared to be uneven. In general, the central sections of chromosomes located with less MYB genes and relatively high densities of GrMYBs were observed in the top and bottom sections of most chromosomes. Especially chromosomes 7 harbored the most R2R3MYB genes (25 myb genes) followed by 24 on chromosome 1 among all chromosomes. Additional, 8 GrMYBs were present on chromosome 5; 10 on chromosome 12; 9 on each of chromosomes 2, 3 and 11; 22 on each of chromosomes 4 and 13; 17, 18, 19 on chromosomes 6, 9 and 8, respectively; and 11 on chromosome 10.

Segmental duplication and tandem duplication events are the main causes of gene-family expansion. Two or more genes located on the same chromosome, one following the other, confirms a tandem duplication event, while gene duplication on different chromosomes or within the same chromosome but not one following the other is designated a segmental duplication event^72^. In the present study, cluster formed by GrMYBs in cotton D genome was identified to elucidate the mechanism behind the expansion of the R2R3-MYB family in cotton. We observed that large-scale segmental duplication and tandem duplication events were logically the contributors to the expansion of the MYB gene family in cotton, following their divergence. According to a whole genome analysis of gene duplications, 27 pairs of MYB genes were produced by segmental duplication, including 25 duplication events between chromosomes as well as 2 duplication events within the same chromosome (GrMYB78 and GrMYB87, GrMYB94 and GrMYB102). At the same time, 16 MYBs were produced by tandem duplication and the others are produced by dispersed duplication (Fig. 4). Interestingly, the gene structure pattern and motifs between GrMYB49/GrMYB50, GrMYB52/GrMYB53, GrMYB109/GrMYB110 and GrMYB135/GrMYB136 were almost identical and their corresponding whole sequences were also very similar (>80%).

To examine the driving force for gene evolution, we performed nonsynonymous and synonymous substitution ratio (ka and ks) analysis of the duplicated genes. A Ka/Ks ratio of 0.5 was suggested as a useful cut-off value to identify genes under positive selection^73^. In our study, eight of the 35 gene pairs had a *ka*/*ks* ratio between 0.5 and 1 (Table 1), suggesting that these genes have likely experienced positive selection. In addition, 27 (77%) pairs of orthologs had a ka/ks ratio <0.5 (Table 1), implying that most cotton R2R3-MYB gene pairs had evolved mainly under the influence of purifying selection.

We further used Ks as the proxy for time to estimate the dates of the duplication events. The segmental duplicated events in cotton appear to have occurred from 6.33 (Ks = 0.19) to 32.56 mya (Ks = 0.98), with an average of 15.68 mya that is consistent with the ages of the genome duplication events^54^. The Ks of tandem duplications of GrMYB genes occurred from 0.97 mya to 14.32 mya, dating the duplication event at 7 mya.

### Quantitative real‐time RT‐PCR analysis of expression profiles for 52 selected MYB genes under drought and salt stress

**Figure 5:**
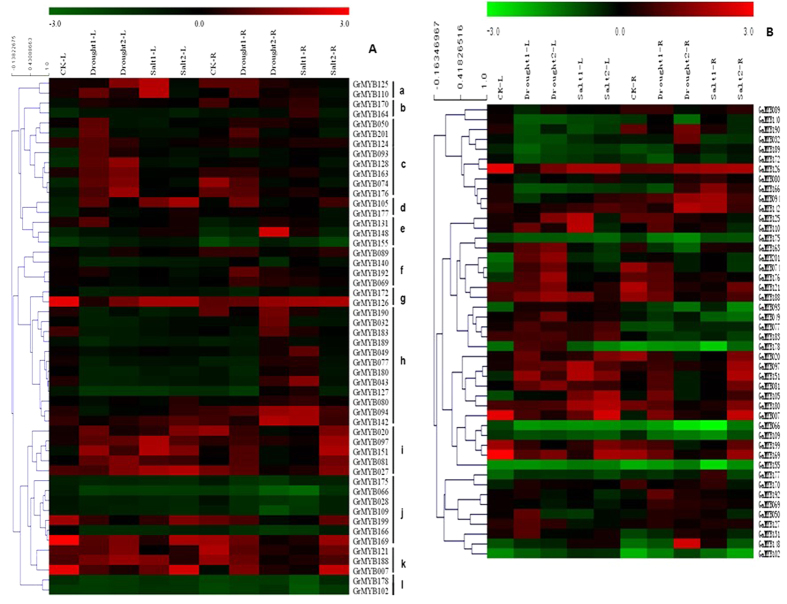
Expression profiles of 52 GrMYB genes under various abiotic stresses. The heat map shows the real-time quantitative RT-PCR (q-RT-PCR) analysis results of GrMYB genes with drought and salinity treatments with three biological and three technical replicates. Expression levels are illustrated by graded color scale. Red, green, and black represent positive, negative, and zero, respectively. The heat map was generated using cluster software. The sources of the samples were as follows: CK-L ( leaves of control), CK-R ( roots of control), Drought-1-L /R(leaves/roots of cotton seedlings under 2.5% PEG treatment), Drought-2-L /R(leaves/roots of cotton seedlings under 5% PEG treatment), Salt-1-L /R(leaves/roots of cotton seedlings under 25mM NaCl treatment), Salt-2-L /R(leaves/roots of cotton seedlings under 50mM NaCl treatment).

**Figure 6:**
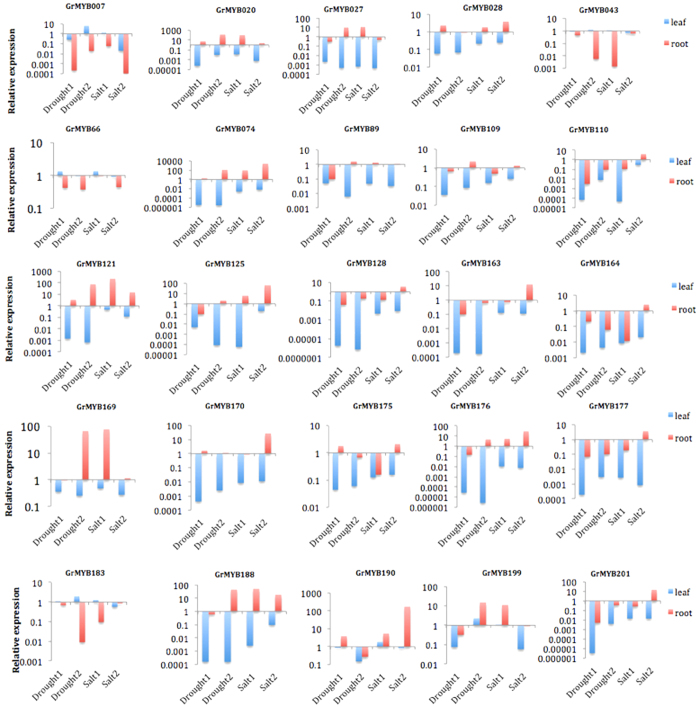
The significantly expressed MYB genes in cotton under salt and drought treatments. Each column represents the mean ± SE of three independent experiments each with three replicates.

Gene expression pattern are usually related to gene’s function. Previous studies have indicated that the MYB TFs play key roles in the regulation of gene expression to adapt environmental change^74^,^75^. It has been reported that the majority of MYB genes are involved in response to diverse abiotic stress, such as drought and high salinity^23^,^45^,^47^,^76^,^77^. Therefore the stress responses of the 52 *G. raimondii* MYB genes chosen from stress-related subgroups and their 46 orthologs in *G. arboreum* were screened using their expression profiles from the quantitative real-time RT PCR (RT-qPCR) under different abiotic stress conditions as shown in Figs 5 and 6. The data were presented with clusters using fold-change values. Most groups of MYB-encoding genes showed preferential accumulation of transcripts in tissues or under a specific condition. The expression of a number of GrMYB and GaMYB genes was significantly altered (Fold change ≥2) upon drought and salt stresses. In *G. raimondii*, all of the 52 GrMYBs having expression profiles were clustered into twelve groups based on their expression pattern (Fig. 5A). Of the 52 analyzed GrMYB genes, the genes in cluster k and I were mainly expressed in leaves and roots under drought and saline conditions. A number of GrMYB genes in cluster c including *GrMYB050, GrMYB074, GrMYB093, GrMYB124* and *GrMYB201* strongly and preferentially expressed in leaves under drought stress, whereas GrMYBs in cluster h were highly expressed in roots under drought and salt stress. The expression pattern of GrMYBs showed that some of the genes clustered in the same subgroup of the phylogenetic tree (Fig. 3A) had similar expression pattern. For example, the expression level of most GrMYBs in subgroup C39 was decreased. However, some GrMYB genes in same subgroup had totally different expression pattern.

In *G. arboreum,* some orthologs of *G. raimondii* were not found, such as *GrMYB27, GrMYB43* and *GrMYB124*. Compared to their orthologs from the DD genome, MYB genes in *G. arboreum* were not clustered into same groups as their Orthologous genes. Furthermore, some GaMYB genes showed different expression pattern, comparing to their orthologs (Fig. 5B). *GaMYB049, GaMYB077* and *GaMYB183* showed highly leaf-specific expression under drought and salt stress, and only slightly in root, while their orthologs have significantly high expression level in root. *GaMYB093* and *GaMYB163* were expressed moderately in leaves under drought stress and had similar but significantly lower expression level in root, when compared with their orthologs from the DD genome. The highest transcript level for *GaMYB066* and *GaMYB109* were detected in leaves and roots under salt stress, respectively, while very low level of expression of these genes were observed in leaves and roots under drought stress.

The relative expression of 25 genes (Fig. 6) were significantly different as stress conditions. The greatest increase in expression (nearly 226 fold) was found in *GrMYB121* at 25 mM NaCl treatment in roots, while the expression of *GrMYB007* and *GrMYB043* demonstrated down regulated with a spike of expression in root under salt stress. Expression of *GrMYB020, GrMYB027, GrMYB074, GrMYB109, GrMYB121, GrMYB125, GrMYB169, GrMYB176* and *GrMYB188* were significantly up-regulated in roots in response to drought stress, but the number of transcripts in leaves decreased under more severe drought conditions, even decreasing to undetectable level in leaves. In contrast, *GrMYB007* and *GrMYB199* were continuously induced and kept a high level in leaves while GrMYB028 and GrMYB190 were significantly down-regulated in seriously drought conditions. The expression of 10 GrMYB genes (*GrMYB028, 074, 121, 125, 169, 170, 176, 177, 188 and 190*) were significantly increased under salt stress, while the expression of *GrMYB007, GrMYB043* and *GrMYB183* was significantly down-regulated in roots (Fig. 6). Additionally, for the salt treatment, transcripts of *GrMYB190, GrMYB183, GrMYB169, GrMYB043 and GrMYB007* initially increased and then dropped in leaves, whereas *GrMYB164* and *GrMYB170* showed opposite response in leaves and roots. Furthermore, eight GrMYBs (*GrMYB109, 110, 128, 163, 164, 175, 177* and *201*) showed a down-regulated trend in low salinity condition and up-regulated at the higher concentration, while *GrMYB027* and *GrMYB199* were down-regulated at low stress condition, but up-regulated at higher concentration treatment. The expression level of *GrMYB066* in leaves and *GrMYB089* in roots did not change significantly under salt treatment. *GrMYB074* was continuously induced in roots with treatments, while *GrMYB066* showed continuously decreased expression level. The expression pattern of cotton MYB genes under different abiotic stress conditions indicated that some of them were major factors involving in cross-talk among different signal transduction pathways in response to abiotic stresses.

## Discussion

### R2R3-MYB gene family and expression pattern in cotton

Genes encoding MYB transcription factors constitute a rather large family of genes in higher plants; R2R3-MYB transcription factors are the more prevalent type among plant MYBs. As more whole plant genomes have been sequenced, many R2R3 genes have been identified in various plant species. In this study, we systematically identified 205 R2R3-MYB members in *G. raimondii* genome. The phylogenetic tree including 205 distinct protein sequences clearly demonstrated that these genes could be divided into 50 subgroups. This classification was further supported by the results of gene structure and motifs analyses. The topology of our phylogenetic tree constructed from R2R3-MYB genes of three species (cotton, rice and *Arabidopsis*) is generally consistent with that derived from *Arabidopsis* alone. In previous studies, R2R3-MYB gene numbers show variety among the species, such as 126 R2R3-MYBs was identified in *A. thaliana*^6^,^9^, 192 in *Populus trichocarpa*^19^, 244 in Soybean^8^, 108 in *Vitis vinifera*^70^, 88 in *Oryza sativa*^18^ and 157 in maize^20^. It indicates that the abundance of R2R3-MYB genes in cotton have expanded, that may be related with genome duplications. The proportion that between the numbers of R2R3-MYB genes in *G. raimondii* and other species is consistent to the rate of the number of predicted genes in genomes. For instance, in dicots, the number of R2R3-MYB genes in cotton is nearly ~1.6 times than in *Arabidopsis* that is congruous with the rate between the numbers of predicted genes in genome (*G. raimondii*: 40,976/*Arabidopsis*: 25,498 ≈ 1.6). In monocots, the number of GrMYBs is approximately ~1.3 times more than that in *Zea mays*, which is consistent to the proportion of predicted genes in their genomes (*G. raimondii*: 40,976/corn: 32,000 ≈ 1.3)^54^,^78^. It suggests that this expansion appeared to be the result of multiple gene duplication processes, including a whole-genome duplication event in the cotton lineage as well as multiple segmental and/or tandem duplication events.

Gene expansion shapes the genome architecture that is important for gene family evolution. The duplicated genes provide the raw materials for the generation of new genes. Segmental duplication, tandem duplication, and transposition events are the main causes of gene-family expansion^79^,^80^. Previous studies revealed that *G. raimondii* has undergone the hexaploidization event shared by all eudicots and a whole genome duplication approximately 13–20 million years ago^54^. In the present study, the average duplication time of GrMYBs (13.7 mya) is very close to the cotton‐specific whole-genome duplication date of *G. raimondii*. Chromosomal distribution analysis revealed that some of the duplication events are likely to have contributed to the expansion of this gene family. Twenty-seven gene pairs were identified as segmental duplication in 205 GrMYBs (26.3%), while 16 GrMYBs (7.8%) were found to be located as tandem repeats in cotton (Fig. 4), indicating that segmental duplications and tandem duplications contributed to the expansion of R2R3-MYB in *G. raimondii*. The results also indicated that, among the duplication events in the GrMYB gene family, the gene pairs that appeared to be derived from segmental duplication events occurred earlier than those that arose from tandem duplication (Table 1). Taken together, our results suggested that the expansion of the MYB gene family in *G. raimondii* mainly arose from whole genome duplication events and segmental duplication, accompanied by tandem duplications. The expansion of other transcription factor gene families in plants has been similarly associated with this process^19^,^20^,^81^,^82^,^83^.

qRT-PCR analysis of the stress-response MYB genes showed some orthologs that were not present in *G. arboreum* responded to the stress treatment indicated that substantial gene expansion occurred in the *G. raimondii* genome. The stress-responsive genes came from the *G. raimondii* and *G. arboreum* genome, suggesting that expansion and contraction in the numbers of R2R3-MYB genes in different cotton species may have altered their adaptation for abiotic stress conditions, and duplications have a significant role in the expansion of the R2R3-MYB gene family in *G. raimondii* after its divergence from *G. arboreum* ~5 million years ago.

### MYB genes as a potential gene network for improving abiotic stresses in cotton

**Figure 7:**
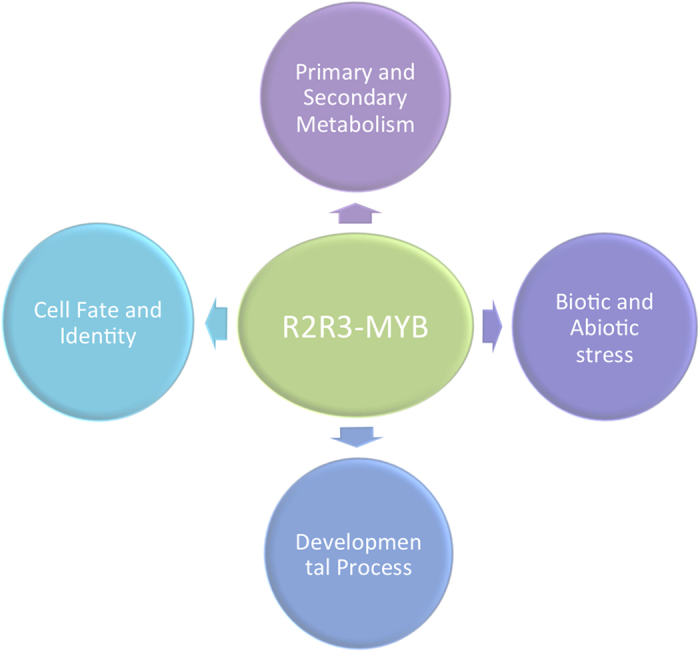
The multiple functions in R2R3-MYB transcriptional factor.

The large size of the MYB family in plants indicates their importance in the control of plant specific processes. The R2R3-MYB genes have been extensively studied and have been found to be involved in a wide variety of roles in plant-specific processes, including primary and secondary metabolism, development and respond to biotic and abiotic stresses (Fig. 7)^6^,^23^,^84^. Plants respond to environmental changes with a number of physiological and developmental changes to tolerate stress. Functional analyses of plant R2R3-MYB genes indicated that they responses to abiotic stress^84^. In this study, 52 selected GrMYB genes and their orthologs in *G. arboreum* that all clustered into the groups with stress response function categories, suggesting that they may have similar functions to the homologous proteins of *Arabidopsis* or rice in abiotic stress condition. The results indicated that they showed different expression pattern in response to salt and drought stress. The relatively high expression level of MYB genes coincident with varied concentration of stress treatment support the hypothesis that MYB plays a key role in the response of cotton to abiotic stress. For example, *GrMYB020, GrMYB074, GrMYB121*, and their orthologs in *G. arboreum* (Figs 5 and 6) showed significantly higher expression level and underwent significant changes in expression during stress treatment compared to their paralogs in these two cotton species. Roots and leaves have very different transcriptome response to abiotic stresses because roots were the tissue directly exposing to the treatment^85^,^86^.

*Arabidopsis* R2R3-MYB transcription factor *AtMYB41* was induced by drought and salt. Transgenic *Arabidopsis* with overexpressing *AtMYB41* was linked to lipid metabolism, cell wall expansion, and cuticle deposition demonstrating a key function of *AtMYB41* in plant drought protection^47^,^87^,^88^. *AtMYB96* is a molecular link that mediates ABA and auxin cross talk in drought stress response and lateral root growth, which provides a fitness adaptation to drought stress. Transgenic Camelina plants overexpressing *AtMYB96* enhanced tolerance to drought^45^,^89^. In our study, the expression of orthologous gene *GrMYB27* and *GrMYB128* was induced significant in the root under drought and salt stress, which were consistent with the biological function of *AtMYB41* and *AtMYB96*. Corresponding to these characterized *Arabidopsis* MYBs, cotton MYBs orthologous genes include *GrMYB020, GrMYB074, GrMYB163, GrMYB170* and *GrMYB201* that also showed significant induction under stresses of drought and/or salt, whereas *GrMYB121, GrMYB169, GrMYB 176, GrMYB188* and *GrMYB190* were strongly induced in response to salt, PEG (Fig. 6). Taken together, the results of these studies and our present results suggest that these members might play a very important role in cotton stress resistance. However, multiple *G. raimondii* MYB genes appeared to participate in responding to one stress stimulus, suggesting that there are multiple signaling pathways involving the response to abiotic stress treatment and the signaling pathways in the plant abiotic stress response are very complicated systems.

Divergence of expression pattern and response to various environmental stresses has been considered as a major reason for retaining duplicated genes in a genome^90^. There are 43 pairs of duplicated MYB genes in *G. raimondii* (Fig. 4). These pairs of genes exhibited quite different level of expression in different tissues and in response to different abiotic stresses. In roots, *GrMYB176* showed dramatically up-regulated in response to salt and drought stresses, while the closely-related gene *GrMYB177* showed down-regulated response to these stresses. This suggests that these genes have functionally diverged after duplication. By comparing the difference expression level of paralogous gene pairs in different tissues and stress conditions, it is suggested that the variable expression pattern of the paralogous gene pairs may be due to a functional divergence that has occurred during the evolutionary history of the MYB family in cotton.

In the present study, the evolutionary origins, phylogenetic relationships and exon/intron structures of R2R3-MYB family members were evaluated in *G. raimondii*, and the tissue expression pattern of the two diploid cotton species were detected. The results offered a useful framework for future research to understand the evolution of R2R3-MYB gene family and the potential physiological role of each MYB gene during abiotic stress. Since only a few MYB genes in cotton have been functionally characterized to date, our results may help in the further study to examine their biological functions, and analyses with knock out mutants and/or gene suppression lines may be useful.

## Additional Information

**How to cite this article**: He, Q. *et al*. Genome-Wide Identification of R2R3-MYB Genes and Expression Analyses during Abiotic Stress in *Gossypium raimondii. Sci. Rep.* **6**, 22980; doi: 10.1038/srep22980 (2016).

## Supplementary Material

Supplementary Information

## Figures and Tables

**Table 1:** Synonymous and nonsynonymous substitution rates and the estimated dates for the duplication events in GrMYB genes.

Gene1	Gene2	ka	ks	ka/ks	Estimated time^65^
GrMYB012	GrMYB167	0.20	0.67	0.29	22.44
GrMYB013	GrMYB082	0.27	0.98	0.27	32.56
GrMYB015	GrMYB096	0.16	0.30	0.52	10.04
GrMYB016	GrMYB054	0.17	0.42	0.41	14.07
GrMYB017	GrMYB099	0.16	0.37	0.42	12.48
GrMYB021	GrMYB084	0.14	0.33	0.42	11.03
GrMYB022	GrMYB075	0.17	0.64	0.27	21.28
GrMYB030	GrMYB068	0.18	0.60	0.30	20.14
GrMYB031	GrMYB115	0.11	0.41	0.26	13.58
GrMYB040	GrMYB048	0.23	0.49	0.46	16.45
GrMYB046	GrMYB116	0.24	0.36	0.65	12.16
GrMYB061	GrMYB129	0.12	0.34	0.36	11.24
GrMYB065	GrMYB088	0.16	0.56	0.29	18.73
GrMYB067	GrMYB187	0.12	0.38	0.31	12.80
GrMYB069	GrMYB124	0.17	0.30	0.58	9.97
GrMYB074	GrMYB177	0.17	0.35	0.48	11.81
GrMYB078	GrMYB087	0.28	0.71	0.40	23.63
GrMYB093	GrMYB128	0.22	0.52	0.42	17.21
GrMYB094	GrMYB102	0.11	0.46	0.24	15.28
GrMYB103	GrMYB117	0.19	0.41	0.47	13.68
GrMYB138	GrMYB168	0.17	0.49	0.34	16.23
GrMYB139	GrMYB174	0.16	0.39	0.40	12.93
GrMYB145	GrMYB191	0.17	0.40	0.42	13.31
GrMYB148	GrMYB155	0.15	0.67	0.22	22.19
GrMYB150	GrMYB195	0.14	0.41	0.33	13.77
GrMYB151	GrMYB164	0.11	0.19	0.57	6.33
GrMYB183	GrMYB189	0.27	0.54	0.51	17.89
GrMYB008	GrMYB009	0.18	0.43	0.41	14.32
GrMYB049	GrMYB050	0.10	0.30	0.32	10.11
GrMYB052	GrMYB053	0.10	0.15	0.64	5.14
GrMYB057	GrMYB058	0.03	0.04	0.76	1.18
GrMYB109	GrMYB110	0.07	0.12	0.53	4.17
GrMYB135	GrMYB136	0.14	0.36	0.39	11.84
GrMYB184	GrMYB185	0.02	0.03	0.67	0.97
GrMYB197	GrMYB198	0.13	0.27	0.47	8.92
