# Supplementary material for: Genome-Wide Identification of R2R3-MYB Genes and Expression Analyses During Abiotic Stress in Gossypium raimondii
Source: Sci Rep. 2016 Mar 24;6:22980. doi: 10.1038/srep22980 (PMC4806351; doi:10.1038/srep22980)
Supplement: Supplementary Information [file srep22980-s1.pdf]

Supporting Information

Genome-Wide Identification of R2R3-MYB Genes and Expression Analyses during Abiotic Stress in *Gossypium raimondii*

Qiuling He [1, 2], Don C. Jones [3], Wei Li [2], Fuliang Xie [1], Jun Ma [1], Runrun Sun[1,4], Qinglian Wang [4], Shuijin Zhu [2], Baohong Zhang [1,*]

[1] Department of Biology, East Carolina University, Greenville, NC 27858, United States of America

[2] Department of Agronomy, Zhejiang Key Laboratory of Crop Germplasm, Zhejiang University, Hangzhou 310058, China.

[3] Cotton Incorporated, Cary, NC 27513, USA

[4] Henan Institute of Science and Technology, Xinxiang, Henan 453003, China

Table S1. Primers for 52 selected GrMYB genes and 46 selected GaMYB genes.

Gene	5'>
GrMYB027-U1	AGAGGGGGAAGTTCTCAATCGAAG
GrMYB027-R1	AAGTCGAGGAGCATGAGTTACAG
GrMYB043-U1	GCTTGTTCTTCAGACAAAAAGGG
GrMYB043-R1	AGCCTGCAACTCTTTCCACA
GrMYB069-U1	TTCGGTCAAGCGTTCCCCTC
GrMYB069-R1	TCGGGAGTCCAGGGACCTTT
GrMYB124-U1	AACCCACAACTGCTACGGCT
GrMYB124-R1	AGCTGGGAACCAGATGTTGCT
GrMYB180-U1	AGTGGTCCGCCATTGCTTGT
GrMYB180-R1	AGACGTTGAGCATGTGTTACAGG
GrMYB140-U1	GGTATGGAAATTGGAGGACGCTG
GrMYB140-R1	ATAGCCGACCACTTATTGCCC
GrMYB163-U1	ACCATTGCCACGCACAAGTC
GrMYB163-R1	GCGGTAGCGGCACGTAATCT
GrMYB166-U1	GCTGGGTTACAAAGGTGTGGA
GrMYB166-R1	TGCAGCTATGGCAGACCACT
GrMYB105-U1	GGGTAACAAATGGGCGGCAA
GrMYB105-R1	TGAAGGGTGGGGGTCCAAAG
GrMYB201-U1	ATGGGCAGCCATAGCATCGT
GrMYB201-R1	CATCACCCGCCATTGAAGGA
GrMYB192-U1	GCCAGCTATGGAACTCCCCC
GrMYB192-R1	TGGTGACGCTCTCATCCAACC
GrMYB020-U1	CACCTTCAAGCCCTCTTAGGCA
GrMYB020-R1	CCCACTGACCTCTACAAATCTGGT
GrMYB177-U1	GGCAACAGATGGGCTGCAAT
GrMYB177-R1	TGCAGCTGATGATGTGAACCC
GrMYB074-U1	AGAGGCCAATGGGAGAGAAAGT
GrMYB074-R1	ACTTGAAGCAGGTTTTGGTGGA
GrMYB93-U1	AACAAGCACTCACCGAGGCAT
GrMYB93-R1	GCACTGGAAGCGTAAGTGGG
GrMYB128-U1	TGAGTTCAAGCTCTCTCACCCT
GrMYB128-R1	TGCGGGTTTGGGTGGATTCT
GrMYB170-U1	TAGGGAATAGGTGGTCGGCA
GrMYB170-R1	TCTGAGTGGCTTGTGGTGGA
GrMYB151-U1	ACGGAATGGGAAAAGTGTGGT
GrMYB151-R1	GGGGTCAAAACACGGTCAGC
GrMYB164-U1	ACCCCGTAATAGTGGCCTGC

GrMYB164-R1	AGGCTCACCACAGTTTTCCCA
GrMYB175-U1	GTAGTTTCATGCCACCAGCAA
GrMYB175-R1	CTGCTCAAACAAGGGGCAGT
GrMYB081-U1	GAAGCTGGTAGGAGAGGCAACA
GrMYB081-R1	AGGCGACAGCTTTTCCCACA
GrMYB028-U1	GGCCTCCCACTTTACCCTCC
GrMYB028-R1	GGAGAGGGTGTGCTGGTGTT
GrMYB199-U1	CTCGTATGGCTGCTCGGCTT
GrMYB199-R1	TAAAGTGGGAGACCTTGCCGT
GrMYB109-U1	CCCTCACAGACCACCACCTC
GrMYB109-R1	TTGAAGCGGAAGGGCTAGGG
GrMYB110-U1	AAATGAGGGTGGTGGGTCCTT
GrMYB110-R1	TCGCACAGCATTCCAATTCCC
GrMYB077-U1	CCCGTGTGCAAAAACAGGCA
GrMYB077-R1	GTCCGAATTTTGCTGCCCCA
GrMYB131-U1	CAAGCACAAGTTGCAGTGAAGA
GrMYB131-R1	GCAACTCTTGCCTGTTCTCCTT
GrMYB007-U1	AGCAGCTTAGAAGAGGAACTTGGT
GrMYB007-R1	GTTCTCCTCAACCCTGCTCGTT
GrMYB032-U1	GCACCCAAAGCTCAGTTCCT
GrMYB032-R1	CTGTGGGACAAATGTTCTGGG
GrMYB125-U1	ACATTGGTCGTCATGGGGTAG
GrMYB125-R1	GTTTCCGCGCTTAATGTCAGG
GrMYB050-U1	CGTTGCGTTTGGATTCCTCGT
GrMYB050-R1	GCCGTCCTGGGAGTCTGATG
GrMYB097-U1	TCAGACTCCCTCGACACCCA
GrMYB097-R1	GACTCCCCACCACCAACCAA
GrMYB049-U1	GCGGTACGTTTGGATCCCTC
GrMYB049-R1	ACGGAAGAGACTTGGGCGT
GrMYB121-U1	GGACGTAAGGAAAGGTCAGTGG
GrMYB121-R1	GGGTGATGCTGCCTCGTCTAA
GrMYB089-U1	ATAGAGCTGAGACGAGGACCA
GrMYB089-R1	TCAAGGGTGATGTTCCCACG
GrMYB080-U1	TGCCCGGAAGAACCGACAAC
GrMYB080-R1	CCGAAGTGGACGCGGTGTTA
GrMYB066-U1	AGATGGATTTGATACGCGGTCCT
GrMYB066-R1	AAACCTGCACAACGAGCAAGG
GrMYB172-U1	ATGGATGTTCGGGGAAGGGA
GrMYB172-R1	CCCGTCCTTTTGAGACCTGC
GrMYB142-U1	GCCCGGTCACAACTCACAAG
GrMYB142-R1	CGCTGCAAAGCCTCGTCTTC
GrMYB148-U1	AACGACGATTCACCTCAGCC

GrMYB148-R1	GGTGAGGCAATGGGCAAACT
GrMYB155-U1	AACACCGGCCTTTTACCCCC
GrMYB155-R1	ACCTTCGGTCATCGACAAGCA
GrMYB102-U1	CGTAAGTGCTTGCCGGTTGG
GrMYB102-R1	TGGGCTCCCTGGACTCATGT
GrMYB094-U1	GACCTAGAAATTGGTCTTTGGTCAG
GrMYB094-R1	GAGGAGTCGGGCTATTGTGG
GrMYB169-U1	TCCGATCACTCACAAGCCAAA
GrMYB169-R1	GAGTGAGGCGGTGTTGAAATG
GrMYB176U1	AAAGCAGCCAACCTTAGCCA
GrMYB176-R1	TCAAGGGTAAAAGCAGGGGG
GrMYB190-U1	CCACACACAAGCCCAAATCG
GrMYB190-R1	AGGCACTGGGATTTTGAGCC
GrMYB127-U1	CAACCTCCCCATCACCGAAA
GrMYB127-R1	GCGGGTGGTAAGATTTGGGA
GrMYB188-U1	GGGCATAAGGAGGTGGGTTT
GrMYB188-R1	TCCAGCTTTCTCAGGCAAGG
GrMYB183-U1	TCGATCCTGTCACCCACAAG
GrMYB183-R1	GTGGTTGCTTTGAGGAGGGT
GrMYB126-U1	GATCGACCCTGCAACTCACA
GrMYB126-R1	GTGGAGGAGCCGATGAAGAC
GrMYB189-U1	CAATGGGAGAGTGCTCGGTT
GrMYB189-R1	TGAGCACATCGAGGCATTGT
GrMYB178-U1	AACCAGCAGGAAGCCATTGA
GrMYB178-R1	GTGGTCAATCATGGCAAGTGT
GaMYB069-U1	GGAAACTGGCGTACTCTCCC
GaMYB069-R1	ATAGCCGACCACTTGTTCCC
GaMYB180-U1	CTTCAAGCTCCCATGACAGA
GaMYB180-R1	TCTGGCTACCATCCATTGAA
GaMYB140-U1	AAAGAGTTGCCGTCTTCGAT
GaMYB140-R1	TGTCTGTTCTTCCAGGCAAC
GaMYB163-U1	CCCATACTCCTCGACTGGAT
GaMYB163-R1	TTCGACTTGTGCATGGAAAT
GaMYB166-U1	AACATGTCGACGAGGTTGCT
GaMYB166-R1	TGGCGGTTGCTGGTATTGAT
GaMYB105-U1	TGACGATGATGGTGATGATG
GaMYB105-R1	AGAAACGATAATGGCGGATT
GaMYB201-U1	ATCTCAGGCCAGGAATCAAA
GaMYB201-R1	TTCTTCAGATGGGTGTTCCA
GaMYB192-U1	GCAGGCTTCGATGGACAAAC
GaMYB192-R1	GCAATGGCTGCCCATTTGTT
GaMYB020-U1	AGGGACTGATCCAATGAAGG

GaMYB020-R1	GCTTTGAGCATTGACGTGTT
GaMYB177-U1	CAACAGATGGGCTGCAATAG
GaMYB177-R1	CGTGAAATCTGGTCTGATGC
GaMYB074-U1	TGGGAATTGGAGGGCTGTTC
GaMYB074-R1	AGCTATTGCAGCCCATCTGT
GaMYB93-U1	CAGCTCAGCCTCATCAACCA
GaMYB93-R1	GTTGAAGGAACTCCGGGTCA
GaMYB170-U1	TAGGGAATAGGTGGTCGGCA
GaMYB170-R1	GACAGTGCATCCGACCAGAA
GaMYB151-U1	TATGTTCGACAAGGCTGCTC
GaMYB151-R1	GAAGGGAGCTCCAACTTCAC
GaMYB175-U1	AAGAAAGGACCGTGGACATC
GaMYB175-R1	AACGTAAGCGGCAACTCTTT
GaMYB081-U1	AGCGGGCTAAGTCAGAATGT
GaMYB081-R1	AATCAGCAACATCGTCAAGC
GaMYB199-U1	CCCACTTTACCCTCCTGAAA
GaMYB199-R1	TGTGGATCGTAGGCAAGAAG
GaMYB109-U1	AATAAATGGGCTCGTATGGC
GaMYB109-R1	CTGGGAATAGAAGGGTTGGA
GaMYB110-U1	ATCCCAACATCACCACCGAC
GaMYB110-R1	CTGCATTGGTGAAAACGGCA
GaMYB077-U1	GTAGTTGCTACGGCATGGAA
GaMYB077-R1	CATGCTCCATATCCCATCTG
GaMYB131-U1	AGGCAAGAGTTGCAGGTTGA
GaMYB131-R1	TCTTCCAGGCAGATGCTGTG
GaMYB007-U1	GCAAAACGAGCAGGGTTGAG
GaMYB007-R1	TGACCACCTGTTACCCCACT
GaMYB032-U1	CATGGACCCTTGAAGAGGAT
GaMYB032-R1	TCAGGTTTCCACGCTTAATG
GaMYB125-U1	TGCAAAACAAGCAGCCATCC
GaMYB125-R1	TGGGCATATGCGTTTGGACT
GaMYB050-U1	AGCCAAGAATTCAGGGACAC
GaMYB050-R1	GGGAGTCTGATGAAGTGCCT
GaMYB097-U1	TCAGACTCCCTTGACACCCA
GaMYB097-R1	CTATGCTCCTCTGTCGCCTG
GaMYB049-U1	AATGCGACGTTAACAGCAAG
GaMYB049-R1	AGGTAGTGGTGGTGGAGGAG
GaMYB121-U1	GAAGCTTGAATGCGACGTTA
GaMYB121-R1	GACCTGGACCGACTCAGTTT
GaMYB089-U1	TGAACTTCACTCTCGTTGGG
GaMYB089-R1	TTGAGTTGCTTGGCATGTTT
GaMYB080-U1	TGTGACGTCAACAGCAAAGA

GaMYB080-R1	GGCAACAACATTTGCTCAAT
GaMYB066-U1	TCAAGGACACCATGCGTTAT
GaMYB066-R1	GTGCTCGAATTCTCCGAAGT
GaMYB172-U1	AACATTTGCCTGGAAGAACC
GaMYB172-R1	GGTAACGCATGGTGTCTTTG
GaMYB142-U1	ACCACTGGAACTCCACCCTA
GaMYB142-R1	GGGTACCGAGGTTCAAGCAA
GaMYB148-U1	ACCATAGTCCGAGCCCATAC
GaMYB148-R1	CGTTCAAATCATCGGTCATC
GaMYB155-U1	TCCAAGTAGTCCATCGGGGT
GaMYB155-R1	CAGAACCGGGTAACGAGAGG
GaMYB102-U1	TAACAAGTGGGCCACCATAG
GaMYB102-R1	CCCTCCATTAGCAACGAAAT
GaMYB094-U1	TCCAGCGTCCCCGTTTTATC
GaMYB094-R1	CAGTGAAGTCGGTGGATCGT
GaMYB169-U1	TCGAATCTCCCAAATCAACA
GaMYB169-R1	TTCCAAAGCCTTTCCATTCT
GaMYB176U1	AGCTGAAGCTCGACTGGTTC
GaMYB176-R1	ATCATCGGCATCGGACTCAC
GaMYB190-U1	TGCTAGGCTCGAAGCAGAAG
GaMYB190-R1	ACAACGTCAAGACACTGGGA
GaMYB127-U1	GACTCACAAGCCCTCAACCT
GaMYB127-R1	GGGACTGCTTCTGGTGAGTT
GaMYB188-U1	CTGATAAAGCTTGGCGTTGA
GaMYB188-R1	AACCTAGCTTCAGCCTCCAG
GaMYB183-U1	TCATGGAGAACACCACAACA
GaMYB183-R1	TGGAATTTGATCACTGCCAT
GaMYB126-U1	TGGGAGAGTGCTCGGTTAGA
GaMYB126-R1	TACATCGAGGCATTGCGGTT
GaMYB189-U1	GCCAGGCTGGTAAGAGAGTC
GaMYB189-R1	CTGGTGACTAAACCTTGCCA
GaMYB178-U1	GGGATTATGGGCAATGGAGGA
GaMYB178-R1	AGCCGACAACTTTTCCCACT

Table S2. List of 205 identified cotton MYB transcription factor genes.

Original ID		Name		Location	
Gorai.001G010000.1	Gorai.001G010000	GrMYB001	Chr01	959975	961727
Gorai.001G015200.1	Gorai.001G015200	GrMYB002	Chr01	1427446	1429657
Gorai.001G020400.1	Gorai.001G020400	GrMYB003	Chr01	1922948	1924536
Gorai.001G020500.1	Gorai.001G020500	GrMYB004	Chr01	1941630	1943118

Gorai.001G020600.1	Gorai.001G020600	GrMYB005	Chr01	1968499	1969954
Gorai.001G024300.1	Gorai.001G024300	GrMYB006	Chr01	2284179	2286009
Gorai.001G075000.1	Gorai.001G075000	GrMYB007	Chr01	7673996	7675174
Gorai.001G087200.1	Gorai.001G087200	GrMYB008	Chr01	9358315	9362986
Gorai.001G087400.1	Gorai.001G087400	GrMYB009	Chr01	9443193	9448317
Gorai.001G090100.1	Gorai.001G090100	GrMYB010	Chr01	9841357	9842696
Gorai.001G107900.1	Gorai.001G107900	GrMYB011	Chr01	12309409	12311484
Gorai.001G115300.1	Gorai.001G115300	GrMYB012	Chr01	13576177	13579065
Gorai.001G138800.1	Gorai.001G138800	GrMYB013	Chr01	18374665	18377897
Gorai.001G143200.1	Gorai.001G143200	GrMYB014	Chr01	19382882	19385359
Gorai.001G148500.1	Gorai.001G148500	GrMYB015	Chr01	20389327	20391486
Gorai.001G169700.1	Gorai.001G169700	GrMYB016	Chr01	24274925	24277071
Gorai.001G177100.1	Gorai.001G177100	GrMYB017	Chr01	26444283	26445987
Gorai.001G205300.1	Gorai.001G205300	GrMYB018	Chr01	39967220	39968780
Gorai.001G207500.1	Gorai.001G207500	GrMYB019	Chr01	40874823	40875813
Gorai.001G239000.1	Gorai.001G239000	GrMYB020	Chr01	47741619	47743217
Gorai.001G249400.1	Gorai.001G249400	GrMYB021	Chr01	50799491	50803775
Gorai.001G263200.1	Gorai.001G263200	GrMYB022	Chr01	53879028	53881423
Gorai.001G263600.1	Gorai.001G263600	GrMYB023	Chr01	53953852	53957268
Gorai.001G268000.1	Gorai.001G268000	GrMYB024	Chr01	54497394	54498755
Gorai.002G009300.1	Gorai.002G009300	GrMYB025	Chr02	606111	607372
Gorai.002G016800.1	Gorai.002G016800	GrMYB026	Chr02	1101940	1105297
Gorai.002G077200.1	Gorai.002G077200	GrMYB027	Chr02	9144204	9145947
Gorai.002G089900.1	Gorai.002G089900	GrMYB028	Chr02	11367102	11369519
Gorai.002G106300.1	Gorai.002G106300	GrMYB029	Chr02	14282313	14284944
Gorai.002G180300.1	Gorai.002G180300	GrMYB030	Chr02	47412504	47414102
Gorai.002G196700.1	Gorai.002G196700	GrMYB031	Chr02	53227891	53229513
Gorai.002G218600.1	Gorai.002G218600	GrMYB032	Chr02	57101040	57102442
Gorai.002G246700.1	Gorai.002G246700	GrMYB033	Chr02	61063059	61064319
Gorai.003G021100.1	Gorai.003G021100	GrMYB034	Chr03	1576363	1578160
Gorai.003G039500.1	Gorai.003G039500	GrMYB035	Chr03	4287453	4288545
Gorai.003G054100.1	Gorai.003G054100	GrMYB036	Chr03	8319150	8320732
Gorai.003G097900.1	Gorai.003G097900	GrMYB037	Chr03	30412950	30414279
Gorai.003G112700.1	Gorai.003G112700	GrMYB038	Chr03	34415733	34416780
Gorai.003G136400.1	Gorai.003G136400	GrMYB039	Chr03	39551225	39552974
Gorai.003G155400.1	Gorai.003G155400	GrMYB040	Chr03	42455373	42457344
Gorai.003G168100.1	Gorai.003G168100	GrMYB041	Chr03	43860897	43862341
Gorai.003G183700.1	Gorai.003G183700	GrMYB042	Chr03	45485383	45487203
Gorai.004G029000.1	Gorai.004G029000	GrMYB043	Chr04	2278700	2281117
Gorai.004G037300.1	Gorai.004G037300	GrMYB044	Chr04	3078926	3080794
Gorai.004G044000.1	Gorai.004G044000	GrMYB045	Chr04	3810733	3812558
Gorai.004G064100.1	Gorai.004G064100	GrMYB046	Chr04	6386072	6387937

Gorai.004G130900.1	Gorai.004G130900	GrMYB047	Chr04	35383172	35384497
Gorai.004G138300.1	Gorai.004G138300	GrMYB048	Chr04	38774326	38776159
Gorai.004G145800.1	Gorai.004G145800	GrMYB049	Chr04	40975674	40976983
Gorai.004G146000.1	Gorai.004G146000	GrMYB050	Chr04	41054383	41055541
Gorai.004G147600.1	Gorai.004G147600	GrMYB051	Chr04	41602813	41604990
Gorai.004G150800.1	Gorai.004G150800	GrMYB052	Chr04	42718120	42719380
Gorai.004G151000.1	Gorai.004G151000	GrMYB053	Chr04	42819188	42820558
Gorai.004G157600.1	Gorai.004G157600	GrMYB054	Chr04	44595515	44598072
Gorai.004G165400.1	Gorai.004G165400	GrMYB055	Chr04	46061863	46065142
Gorai.004G172700.1	Gorai.004G172700	GrMYB056	Chr04	47373415	47375535
Gorai.004G183500.1	Gorai.004G183500	GrMYB057	Chr04	49706791	49707986
Gorai.004G183600.1	Gorai.004G183600	GrMYB058	Chr04	49735278	49736661
Gorai.004G183700.1	Gorai.004G183700	GrMYB059	Chr04	49741434	49742864
Gorai.004G196800.1	Gorai.004G196800	GrMYB060	Chr04	51729563	51730684
Gorai.004G201900.1	Gorai.004G201900	GrMYB061	Chr04	53026504	53028439
Gorai.004G231100.1	Gorai.004G231100	GrMYB062	Chr04	56776023	56777881
Gorai.004G268300.1	Gorai.004G268300	GrMYB063	Chr04	60335281	60337017
Gorai.004G269400.1	Gorai.004G269400	GrMYB064	Chr04	60429562	60432614
Gorai.005G054900.1	Gorai.005G054900	GrMYB065	Chr05	5568625	5569666
Gorai.005G068900.1	Gorai.005G068900	GrMYB066	Chr05	7343104	7345268
Gorai.005G142000.1	Gorai.005G142000	GrMYB067	Chr05	38607519	38609832
Gorai.005G162600.1	Gorai.005G162600	GrMYB068	Chr05	47205925	47208903
Gorai.005G206300.1	Gorai.005G206300	GrMYB069	Chr05	58525968	58527381
Gorai.005G206800.1	Gorai.005G206800	GrMYB070	Chr05	58616362	58617555
Gorai.005G234900.1	Gorai.005G234900	GrMYB071	Chr05	61741646	61742938
Gorai.005G254000.1	Gorai.005G254000	GrMYB072	Chr05	63160590	63162320
Gorai.006G015200.1	Gorai.006G015200	GrMYB073	Chr06	3354943	3356681
Gorai.006G017400.1	Gorai.006G017400	GrMYB074	Chr06	4075329	4076944
Gorai.006G030800.1	Gorai.006G030800	GrMYB075	Chr06	7977274	7979550
Gorai.006G037500.1	Gorai.006G037500	GrMYB076	Chr06	10506877	10508283
Gorai.006G060300.1	Gorai.006G060300	GrMYB077	Chr06	22101723	22102879
Gorai.006G064000.1	Gorai.006G064000	GrMYB078	Chr06	23639843	23641669
Gorai.006G073500.1	Gorai.006G073500	GrMYB079	Chr06	29387144	29388124
Gorai.006G080800.1	Gorai.006G080800	GrMYB080	Chr06	30828162	30830523
Gorai.006G121900.1	Gorai.006G121900	GrMYB081	Chr06	37292368	37300348
Gorai.006G129100.1	Gorai.006G129100	GrMYB082	Chr06	38252629	38256479
Gorai.006G162800.1	Gorai.006G162800	GrMYB083	Chr06	42230328	42232051
Gorai.006G172800.1	Gorai.006G172800	GrMYB084	Chr06	43198705	43202398
Gorai.006G179700.1	Gorai.006G179700	GrMYB085	Chr06	43727765	43730347
Gorai.006G192400.1	Gorai.006G192400	GrMYB086	Chr06	44945490	44946782
Gorai.006G195700.1	Gorai.006G195700	GrMYB087	Chr06	45275944	45279007
Gorai.006G254200.1	Gorai.006G254200	GrMYB088	Chr06	49702966	49704317

Gorai.006G265800.1	Gorai.006G265800	GrMYB089	Chr06	50472152	50474420
Gorai.007G001800.1	Gorai.007G001800	GrMYB090	Chr07	197160	198238
Gorai.007G002200.1	Gorai.007G002200	GrMYB091	Chr07	227372	230478
Gorai.007G011100.1	Gorai.007G011100	GrMYB092	Chr07	876661	878200
Gorai.007G037100.1	Gorai.007G037100	GrMYB093	Chr07	2576395	2578078
Gorai.007G055600.1	Gorai.007G055600	GrMYB094	Chr07	3920682	3922729
Gorai.007G074500.1	Gorai.007G074500	GrMYB095	Chr07	5274358	5275325
Gorai.007G118200.1	Gorai.007G118200	GrMYB096	Chr07	9290190	9292934
Gorai.007G121000.1	Gorai.007G121000	GrMYB097	Chr07	9520124	9522037
Gorai.007G124000.1	Gorai.007G124000	GrMYB098	Chr07	9892396	9894830
Gorai.007G146200.1	Gorai.007G146200	GrMYB099	Chr07	12349029	12350685
Gorai.007G179300.1	Gorai.007G179300	GrMYB100	Chr07	16727088	16728629
Gorai.007G192900.1	Gorai.007G192900	GrMYB101	Chr07	18915887	18917647
Gorai.007G218100.1	Gorai.007G218100	GrMYB102	Chr07	24850129	24851061
Gorai.007G227500.1	Gorai.007G227500	GrMYB103	Chr07	27456478	27458089
Gorai.007G236800.1	Gorai.007G236800	GrMYB104	Chr07	32093876	32095145
Gorai.007G252700.1	Gorai.007G252700	GrMYB105	Chr07	40360237	40362000
Gorai.007G253200.1	Gorai.007G253200	GrMYB106	Chr07	40638426	40640763
Gorai.007G260600.1	Gorai.007G260600	GrMYB107	Chr07	42794266	42796412
Gorai.007G268800.1	Gorai.007G268800	GrMYB108	Chr07	45011316	45013670
Gorai.007G312400.1	Gorai.007G312400	GrMYB109	Chr07	52613645	52615821
Gorai.007G312700.1	Gorai.007G312700	GrMYB110	Chr07	52661130	52664039
Gorai.007G346400.1	Gorai.007G346400	GrMYB111	Chr07	57434322	57436192
Gorai.007G348600.1	Gorai.007G348600	GrMYB112	Chr07	57887881	57889534
Gorai.007G350500.1	Gorai.007G350500	GrMYB113	Chr07	58067193	58068885
Gorai.007G362200.1	Gorai.007G362200	GrMYB114	Chr07	59387412	59390851
Gorai.008G035700.1	Gorai.008G035700	GrMYB115	Chr08	4335881	4337235
Gorai.008G060100.1	Gorai.008G060100	GrMYB116	Chr08	9542441	9544133
Gorai.008G086400.1	Gorai.008G086400	GrMYB117	Chr08	18938519	18940194
Gorai.008G117400.1	Gorai.008G117400	GrMYB118	Chr08	35273907	35275845
Gorai.008G126700.1	Gorai.008G126700	GrMYB119	Chr08	36806220	36807699
Gorai.008G133700.1	Gorai.008G133700	GrMYB120	Chr08	38033631	38035360
Gorai.008G137300.1	Gorai.008G137300	GrMYB121	Chr08	38734814	38736065
Gorai.008G141000.1	Gorai.008G141000	GrMYB122	Chr08	39244456	39245895
Gorai.008G151100.1	Gorai.008G151100	GrMYB123	Chr08	40702373	40704365
Gorai.008G151400.1	Gorai.008G151400	GrMYB124	Chr08	40788246	40789547
Gorai.008G158000.1	Gorai.008G158000	GrMYB125	Chr08	42299918	42301289
Gorai.008G179600.1	Gorai.008G179600	GrMYB126	Chr08	45658105	45659814
Gorai.008G179800.1	Gorai.008G179800	GrMYB127	Chr08	45683230	45685232
Gorai.008G192900.1	Gorai.008G192900	GrMYB128	Chr08	47677489	47679417
Gorai.008G238800.1	Gorai.008G238800	GrMYB129	Chr08	52570763	52572329
Gorai.008G277900.1	Gorai.008G277900	GrMYB130	Chr08	55554827	55557101

Gorai.008G287100.1	Gorai.008G287100	GrMYB131	Chr08	56260623	56262754
Gorai.008G293100.1	Gorai.008G293100	GrMYB132	Chr08	56682637	56685453
Gorai.008G295800.1	Gorai.008G295800	GrMYB133	Chr08	56893345	56894808
Gorai.009G006700.1	Gorai.009G006700	GrMYB134	Chr09	572162	573751
Gorai.009G040800.1	Gorai.009G040800	GrMYB135	Chr09	3017583	3019191
Gorai.009G040900.1	Gorai.009G040900	GrMYB136	Chr09	3021881	3022948
Gorai.009G110800.1	Gorai.009G110800	GrMYB137	Chr09	8094615	8096288
Gorai.009G142200.1	Gorai.009G142200	GrMYB138	Chr09	10770738	10772733
Gorai.009G146500.1	Gorai.009G146500	GrMYB139	Chr09	11139656	11141013
Gorai.009G174500.1	Gorai.009G174500	GrMYB140	Chr09	13447354	13449055
Gorai.009G208900.1	Gorai.009G208900	GrMYB141	Chr09	16200751	16202181
Gorai.009G215900.1	Gorai.009G215900	GrMYB142	Chr09	16796205	16797379
Gorai.009G222900.1	Gorai.009G222900	GrMYB143	Chr09	17347243	17348899
Gorai.009G236900.1	Gorai.009G236900	GrMYB144	Chr09	18731247	18732722
Gorai.009G251700.1	Gorai.009G251700	GrMYB145	Chr09	20486081	20488408
Gorai.009G263200.1	Gorai.009G263200	GrMYB146	Chr09	21766462	21768042
Gorai.009G275100.1	Gorai.009G275100	GrMYB147	Chr09	23044460	23045101
Gorai.009G276400.1	Gorai.009G276400	GrMYB148	Chr09	23188450	23189304
Gorai.009G281000.1	Gorai.009G281000	GrMYB149	Chr09	23793609	23794878
Gorai.009G288900.1	Gorai.009G288900	GrMYB150	Chr09	24787370	24788923
Gorai.009G301100.1	Gorai.009G301100	GrMYB151	Chr09	26945119	26951323
Gorai.009G413600.1	Gorai.009G413600	GrMYB152	Chr09	63211445	63212474
Gorai.009G454000.1	Gorai.009G454000	GrMYB153	Chr09	70528760	70530201
Gorai.010G016800.1	Gorai.010G016800	GrMYB154	Chr10	1269361	1270773
Gorai.010G023500.1	Gorai.010G023500	GrMYB155	Chr10	1863674	1865585
Gorai.010G028800.1	Gorai.010G028800	GrMYB156	Chr10	2367547	2368521
Gorai.010G079900.1	Gorai.010G079900	GrMYB157	Chr10	11607138	11608670
Gorai.010G087200.1	Gorai.010G087200	GrMYB158	Chr10	13355067	13356532
Gorai.010G096800.1	Gorai.010G096800	GrMYB159	Chr10	16431787	16433146
Gorai.010G110000.1	Gorai.010G110000	GrMYB160	Chr10	20805182	20810817
Gorai.010G139300.1	Gorai.010G139300	GrMYB161	Chr10	33332574	33334368
Gorai.010G148300.1	Gorai.010G148300	GrMYB162	Chr10	39256503	39257828
Gorai.010G149300.1	Gorai.010G149300	GrMYB163	Chr10	40064323	40065625
Gorai.010G212500.1	Gorai.010G212500	GrMYB164	Chr10	57860099	57864460
Gorai.011G021300.1	Gorai.011G021300	GrMYB165	Chr11	1496258	1498528
Gorai.011G023800.1	Gorai.011G023800	GrMYB166	Chr11	1711464	1713121
Gorai.011G044100.1	Gorai.011G044100	GrMYB167	Chr11	3312189	3314568
Gorai.011G063900.1	Gorai.011G063900	GrMYB168	Chr11	5380565	5382413
Gorai.011G122800.1	Gorai.011G122800	GrMYB169	Chr11	17131798	17134197
Gorai.011G173900.1	Gorai.011G173900	GrMYB170	Chr11	39346543	39348101
Gorai.011G195100.1	Gorai.011G195100	GrMYB171	Chr11	47058124	47061069
Gorai.011G203900.1	Gorai.011G203900	GrMYB172	Chr11	49308106	49309787

Gorai.011G278500.1	Gorai.011G278500	GrMYB173	Chr11	61060995	61063770
Gorai.012G011700.1	Gorai.012G011700	GrMYB174	Chr12	1323016	1324130
Gorai.012G022000.1	Gorai.012G022000	GrMYB175	Chr12	2710898	2715690
Gorai.012G052500.1	Gorai.012G052500	GrMYB176	Chr12	6833486	6835761
Gorai.012G054500.1	Gorai.012G054500	GrMYB177	Chr12	7272830	7274538
Gorai.012G061800.1	Gorai.012G061800	GrMYB178	Chr12	8711631	8712762
Gorai.012G078200.1	Gorai.012G078200	GrMYB179	Chr12	12605107	12606381
Gorai.012G132200.1	Gorai.012G132200	GrMYB180	Chr12	30142713	30143807
Gorai.012G132400.1	Gorai.012G132400	GrMYB181	Chr12	30195617	30196643
Gorai.012G149000.1	Gorai.012G149000	GrMYB182	Chr12	32323029	32324043
Gorai.012G186500.1	Gorai.012G186500	GrMYB183	Chr12	35342381	35343800
Gorai.013G071600.1	Gorai.013G071600	GrMYB184	Chr13	8427885	8429164
Gorai.013G072900.1	Gorai.013G072900	GrMYB185	Chr13	8716277	8717437
Gorai.013G073000.1	Gorai.013G073000	GrMYB186	Chr13	8794152	8795019
Gorai.013G086400.1	Gorai.013G086400	GrMYB187	Chr13	12884531	12887077
Gorai.013G088200.1	Gorai.013G088200	GrMYB188	Chr13	13546998	13548246
Gorai.013G088300.1	Gorai.013G088300	GrMYB189	Chr13	13624584	13626067
Gorai.013G088400.1	Gorai.013G088400	GrMYB190	Chr13	13773959	13775670
Gorai.013G106600.1	Gorai.013G106600	GrMYB191	Chr13	22968509	22970105
Gorai.013G113000.1	Gorai.013G113000	GrMYB192	Chr13	27367333	27368577
Gorai.013G114900.1	Gorai.013G114900	GrMYB193	Chr13	28442942	28444636
Gorai.013G118300.1	Gorai.013G118300	GrMYB194	Chr13	29482996	29484163
Gorai.013G139200.1	Gorai.013G139200	GrMYB195	Chr13	37519857	37521249
Gorai.013G143000.1	Gorai.013G143000	GrMYB196	Chr13	39214321	39215373
Gorai.013G143100.1	Gorai.013G143100	GrMYB197	Chr13	39247984	39248979
Gorai.013G143200.1	Gorai.013G143200	GrMYB198	Chr13	39286109	39287071
Gorai.013G159200.1	Gorai.013G159200	GrMYB199	Chr13	43497073	43500294
Gorai.013G196500.1	Gorai.013G196500	GrMYB200	Chr13	50052381	50054179
Gorai.013G196800.1	Gorai.013G196800	GrMYB201	Chr13	50181346	50182812
Gorai.013G246200.1	Gorai.013G246200	GrMYB202	Chr13	56496323	56498095
Gorai.013G251100.1	Gorai.013G251100	GrMYB203	Chr13	56881058	56884423
Gorai.013G265300.1	Gorai.013G265300	GrMYB204	Chr13	57816908	57818823
Gorai.013G269300.1	Gorai.013G269300	GrMYB205	Chr13	58094145	58095592

Figure S1. Amino acid sequences of 205 identified cotton MYBs.

>GrMYB001

MVRAPCCDKSNVRKGPWSPDEDNTLVNYIHKHGTGGNWITLPRKAGLKRCGKSCRL
RWLNYLRPDIKHGGFSEEEDNIICSLYSTLGSRWSVIAAQLPGRTDNDIKNHWNTKLK
KKLLAAKIKGGNNEITTTISAAPFCKAVGSDGSSRTTSSSYMTDMKTYQKKYYEYPAL

VLDQTDQFSMPYLPFENNYGAAWCSNGVSNEGQGMMVDHFVDFEVDPQHVLSGSSF
QEENINGVGDDPWFGILSATHYNIFD

>GrMYB002

MGRRPCCAKEGLNKGAWTANEDQILKNYITIHGEGKWRDLPQKAGLKRCGKSCRLR
WLNYLRPDIKRGNISIEEEELIIRLHKLLGNRWSLIAGRLPGRTDNEIKNYWNTNLSKR
MEGQKRNISPNTPQVSSHNVIWTKAVKCTKAVNVDTLDPKSETLVNSPSETPSSSAINE
DNANNNSMDFLVDFDIDELLAFEYNPIPELYRTQNRGDKMGNNVADNGEYSHGTEFC
QACEHLDLRGLESYLNLEDEWIN

>GrMYB003

MGRSPSCPKEGLNKGAWTALEDKLLASYIKAHGEGKWSDLSRRAGLKRCGKSCRLR
WLNYLRPDIKRGNISRDEEELIIRLHNLLGNRWSLIAGRLPGRTDNEIKNYWNTVLRK
RAKGQASIGTPPSKSTHKMLSTESGVCEPLRSPQATKAKIKVILTKATRSSSKVRIPTQL
PAAQHTDKLRDGQLNSTNNNQEMSGDIATVEAHSGTQVLDSLYSDGGGSDLLNEQLK
ERDVEFEENPMQPLAFDEAMFKNWTVNHCLNGNDAMDFDSLAFLLETDEYSFCRNI
NFQT

>GrMYB004

MDLYKQRERVGEIRSMGKSSCCSKEGLKKGAWTDWEDKILASYINVHGEGKWRNLP
KRAGLKRCCRSCRLRWVNYLRPDIKRGNISHDEEELIIRLHNLLGNRWSLIAGRLPGRT
DNEIKNYWNSTLSKRTKAQASIKYPRSSNFTHKTIPIESKLKESSKRSAIQTKAIGGSSK
VMVPTQPPTSQDIDKLRDGKLYSTNNSQEMGGDIATVEAHNGTQVLDSLNSDGGSDL
LSFEINELRKASDGNFEENRMQLLPFDEAMFEDWTTYPYLNDNAATGWDSLAFLIDS
NDWP

>GrMYB005

MGRSPCCSKEGLNKGAWTALEDKILTSYIHVHGEGKWRNLPKRAGLKRCGKSCRLR
WLNYLRPDIKRGNISHDEEELIIRLHNLLGNRWSLIAGRLPGRTDNEIKNYWNTTLGK
RAKAQASIEAKTIPTESRLNKPSKSSTKIEVIRTKAIRCSSKVMVPLQPPATHQHGQHHY
TNNNEEMGGGISTIEAHNGIQMLESLYSDGGSNLLSFEINELLKSHDGGEFEENPMQQ
HFPLGEAMLKDWSTCPCLDDNGATDLESLAFLLDTDEWP

>GrMYB006

MGRAPCCDKANVKRGPWSPEEDATLKNYVQSHGTGGNWIALPLKAGLKRCGKSCRL
RWLNYLRPDIKHGGFTEEEDNIICSLYSQMGSRWSLIASQLPGRTDNDVKNYWNTKLK
KKLLAGKSSFNVENNGGLLNANNNNIPTQPLPYNLDYSTTSLPILSDVSYGFSVSNCST
SQNMGLDPVMQFSTPDTITNLSQSGPNLDNSHNNIVVVSSSQEGSALSDSTGNGYGED
TGSIILMDDQQFSYEFPYEFVNGVLAGPSFEFSHGDIKTSQGLNQSVATNPY

>GrMYB007

MLSYTNSSKGDAEQLRRGTWSAEEDSLLIHYIGLHGEGHWNLLAKRAGLRRTGKSCR
LRWLNYLKPDVKHGNLTPQEECLILQLHSKWGNRWSKIAKYLPGRTDNEIKNYWRTR
VNKHARHLKIDANSTPFQHVIRCCNWMPRLLPEMESLNQTSQHDQELLTMEQVPVQE
QISGSTDIMSTISQVAEYQTSPFSIISNNDDYALAKYCYHDDNIDKNCCYYNLASTSAFE
DFPYLVGDCHKPDYNCVNDGFADGLWSMGEQ

>GrMYB008

MEGSSLRVRKGAWTEEEDLLLKKCIEKYGEGKWHQVPARAGLNRCRKSCRLRWLNY
LKPNIKRGHFAADEVDLIIRLHNLLGNRWSLIAGRLPGRTANDVKNYWNTHLLKKIID
TSGKNSKPKSYQPNPNTKIIKPRPHILSKHSFLISLDEYNNNNNNNHAEASNNVALAND
GNDDYGYCFPNDHDEMMWWENMMINEKDVDGYQLQCSANDFDQSVLDQPMNED
NYGSIIDEVFLDEELWNVFNP

>GrMYB009

MEGSSLRVRKGAWTEEEDLLLKKCIEKYGEGKWHQVPARAGLNRCRKSCRLRWLNY
LKPNIKRGHFAADEVDLIIRLHNLLGNRWSLIAGRLPGRTANDVKNYWNTHLLKKNID
TFTKTSNPKSYQLKSNTKVIKPRPQILSKRNFLVNLDEYNNNNNNNNNNRAETSNNVV
LANCGDNHGGYCFPDHDEIMWWENMMMNEKKVDGHQLQCSANDIDESVLDQIMN
EDNFGNTMDELFLDEEPWNVFNP

>GrMYB010

MMVRGAPKQAWRKGPWTHEEDKLLAEYVTFHGEGRWSSVARSTGLNRSGKSCRLR
WVNYLRPGLKRGHITPQEEGIMVELHALWGNKWSTIAKYLPGRTDNEIKNYWRTHF

MKKERSAQRQQKRKALKLKQQPPQPNKEETAAVGDGGGATDHEGRMCEEAATLLDE
YLMNEGLWWNQQQQANNIAMQMQQVACNCYGGGVISNGGCIF

>GrMYB011

MVQDEIRKGPWTEQEDILLANFVHLFGDRRWDFIAKVSGLNRTGKSCRLRWVNYLHP
GLKRGKMTPHEERLVLELHAKWGNRWSRIARKLPGRTDNEIKNYWRTHMRKTAQEK
KRAIPISPSSSSSNCHSSSSTVTTVDSLPSSGTGNIVSFYDTGGLDMAGKKNSPEFEDGN
GYSMDDIWKDIDMPEEDTIIKPLPDNYSQQGCNFSWEYCWDSLWKMDDEEESKMFF
PPNQLVSCFDFGTESVTG

>GrMYB012

MKELRGRRVAFGYGNLPCNNNYKSSLCKSSPPLSAIDRFLWGQTQSLSPHSQAAHERP
YSIQNNVKINKGTVLGSTADALLRGFSFPNDAIGGYLPTQTNPEESFLDGLFVDGEILA
LTDDKNPNMEMKASIKGDFPKGVVKRNKKVASAALIKGQWTDDEDRKLIRLVKQYG
VRKWAQIAESLVGRAGKQCRERWHNHLRPDIKKDSWSEEEERILIEAHAKVGNRWAE
IAKFIPGRTENAIKNHWNATKRRQNSRKKNKQNDNQNGKPQSSILQDYIRSQNLNTPT
NSSTTSATPSSSTFSENLSTQFKYFLPEPSESDDSHPLVAQTYDDELMFMQNFFANNNN
TIHPSLDYSQTRNPTEVFKSVHVVDHNLSKGSSTIDCTTFADTRFGFSSSIQEPNNEPRT
TYLFSDLYLSRLLNGATTSSFPNGDDYYNGDVNTNLLSEQASSDGRKEMDLIEMVSSC
QFYP

>GrMYB013

MMRKPDPSVKVSSGGNTNTNTNTNTNNGTTTNKLRKGLWSPEEDDKLINYMLTNGQ
GCWSDVARNAGLQRCGKSCRLRWINYLRPDLKRGAFSPQEEELIVHLHSILGNRWSQI
AARLPGRTDNEIKNFWNSTIKKRLKNLSSTPSPKVSNSSTSEPNNDAMQGLMSMQEQ
GILPMYMDLPSASSNSSLQSMVLNHTGNSLLMLEHDLNVFGASGYFDPASCVTQVGV
NGESFYGENEMLGSVENGAERELYVPPLESIGENLKTENTTVDVWNINNNHFNIINSK
NNNSKSDNIGNAAVGNFWIGEELKVGEWDLENLMKDVSSFPFLDFQS

>GrMYB014

MGRAPCCEKVGLKKGRWTAEEDVLLTKYIQANGEGSWRSLPKNAGLLRCGKSCRLR
WINYLRADLKRGNFTSQEEEVIINLHATLGNRWSLIASYLPGRTDNEIKNYWNSHLSR
KIHSFRRPLTQSMPVIMDLTKTAVIAKRKGGRNSKGSMKENKSRSTQKDTGSCSNKPT

ENVCVNEVVPFPSTPLLEKETLSTTAIEDRMVLDQHGEDKERTTHVVPSPCHDTVVEG
MLGSSEERESLVSEEGTIENSMQCPSGNAEKGTGILAPHESIDSSEIEWFNDILDSELLQ
PSGDLTFTELGEDSGNVKTHTTAANNEEIVSRNCSADSGGDLSSCTSTTFYFVDDWEW
ENVVPRSELWDEKEYMCSWLWEPSDYHGKGERHKVDDNGFEGHNPMIAANASLFS

>GrMYB015

MGRHSCCYKQKLRKGLWSPEEDEKLLRHITKYGHGCWSSVPKQAGLQRCGKSCRLR
WINYLRPDLKRGTFSQEEENLIIELHAVLGNRWSQIAAQLPGRTDNEIKNLWNSCLKK
KLRQRGIDPVTHKPLSEVENGGEDSKSQPTNSLDMATSGASTELNLNTDNPKAGPPSV
TAHHFQLEMEGSPCSNKINSSNNSSKDLFMDSQPSDLVGHFPIQQLNYASNARLSSTTS
NPTLWFTQTSKPFDINSEFPSTSMSALLPPLTSSFLSAPMGFKPSDTPSIPSFTHINGSRY
WETAASANNSNSSSSTELQSNNSFFENNSYSWGLTDCSTSEKEAPNPIHLMETQADEIK
WPEYLNNPLLMAAALQNQTPQSFCNIEIKSETDFFTNTSSNTVWSLNQQQQQQQQQ
AALQNSDMCAKDIQRLTAAYGHI

>GrMYB016

MGRRPCCERKGLKKGPWGPEEDEILINYINKHGHGSWRSLPKLSALWKFLFCRVSPLQ
PIKGLISTLTGLRRCGKSCRLRWTNYLRPDIKRGPFTLDEEKLVIQLHAILGNRWAAIAS
QLPGRTDNEIKNLWNTHLKKRLLCMGVDPLTHEPFTSGGPTTRPRSSPATRHMAQWES
ARLEAEARLSKESLFFNSPPTSVKPDPDFFLRLWNSEVGESFRKLNGEAKADCRSPISQ
ASSSTKCGSVSGVTIDVGPIAAGSSTPKSSRTEDPIMFDASFSSSSNESEDSSDTALQLLL
DFPINNDMSFLDDVDTYATPPQC

>GrMYB017

MGRAPCCEKMGLKKGPWTPEEDQILINYINLYGHGNWRALPKQAGLLRCGKSCRLR
WTNYLRPDIKRGNFSREEEDTIINLHEMLGNRWSAIAARLPGRTDNEIKNVWHTHLKK
RLKPNHGSIENKRQPKSNVDSSKDIKKEQQEPMNFLSPAVNAADNRPASPPESSSEVST
VTTSENNSNMCMNKIETQEYVSEIDENFWSEVLSADNSSIASDLQVAGSDQQLHPQYL
PSSPLPKLETINDYGSNLYDTDTNMDFWYNLFARVGDLSELPEI

>GrMYB018

MGRQPCCDKVGLKKGPWTAEEDKKLFNFILTNGQCCWRAVPKLAGLLRCGKSCRLR
WTNYLRPDLKRGLLSEHEEKMVIDLHAQLGNRWSKIASHLPGRTDNEIKNHWNTHIK

KKLRKMGIDPLTHKPLTTTQEHHPQQQRDEQKKQASKPDDARSQAPKEPEAETSLKS
TITEAKEEEPMNNIGIDGFCTDEVPLIESHEILVRNPAPSISSSSSSSCHSSMFLEELQFSD
FEWPCDDYNTSNKELSLWDDDFNIWDFLSNDDTDKKLALDSLSSPLMQCPRMGFDQ
DSRPYQLL

>GrMYB019

MMGWEAPKIGLRKGPWTPQEDKLLTEYVNMHGEGRWSSVARSSGLNRSGKSCRLR
WVNYLRPGLKRGQITPQEEGIIIELHALWGNKWSTIARYLPGRTDNEIKNYWRTHYKK
KEKSTLKQQKRKAEILKLKQQQQQEKPDKDEGEDGKVNSEAVEITNHQSEGKQQMV
FMYPSSEDQCLAMMSQEPANAASWIDQYLVDEGLWSGLWNLDDDHHQPGNCCNNIA
MQSQADTDYNNSFGGMQATCTMEGTFSREP

>GrMYB020

MGRQPCCDKVGVKKGPWTPEEDLILVSYIQQHGPGNWRAVPTKTGLLRCSKSCRLRW
ANYLRPGIRRGNFTENEEKMIIHLQALLGNRWAAIASYLPERTDNDIKNYWNTHLKKK
LQGNESSSRYGFPSSSSSYQICRGQWERKLQTDIHMAKKDLSDALSPEKSSDLVEMKP
FNNHTSSPKPSGYASSTENIAKLLKGWMRNNPWKMADSADYSEEGTVPMKEDKNSK
EMAEAFQSHLGFESLDSSLSDISPSMSPETSLSQYESKPHLNAQSQLSLLEKWLFDEGK
DYQLCDITLDQNLYFF

>GrMYB021

MAELRLEERCLYKKQLTTASSSSVSEGGGNAVVKSPGVSSPAPTSPNHRRTTGPIRRAK
GGWTPEEDETLRNAVAAFKGKSWKKIAEFFPDRSEVQCLHRWQKVLNPDLVKGPWT
QKEDDKIIELVSKYGPTKWSVIAKSLPGRIGKQCRERWHNHLNPDIKKDAWTMEEEL
ALMNAHRTYGNKWAEIAKVLPGRTDNAIKNHWNSSLKKKLDFYLATGKLPPVSKNG
LQNGTKDIRTPTTQNFPVFLKKESGSTAQTSSGTTDTCKLEEDGKDQLESSAPVQDMT
ASTNVIPEEPTDTESTECKLQSVDINPCYCTSESGGKFGSHRISSQVVEILKEQVGLDTP
TYCSLCCDESPRSQHEWTSTPITSPISFFTPPRVKGSGLSTHSPEYILRIAAKSFLNTPSIF
RKRKMGAQVLTLPNKKGKLNEEIGKDGIQLSGEQQRTENSSEQAQFCDGDPCESPAC
QGNSPTLPNSTVFNASPPYRLRSKRTAVLMSVERKLELTFDKEA

>GrMYB022

MDKKPCKSQEVEVRKGPWTMEEDLILINYIANHGEGVWNSLAKAAGLKRTGKSCRL
RWLNYLRPDVRRGNITPEEQFLIMELHTKWGNRWSKIAKHLPGRTDNEIKNYWRTRI
QKHMNKPAEVCYSNQSDCELLDQQASSSQLPTNTTNADFMGTSYSPLSSFDYYNNIE
AFSGQQQQQQIMLQSPSNQEDNYWNMEDIWSMQILNGN

>GrMYB023

MEYDTSFKEDFPFLSSLFSENNSSSSFKPDFNGCFPLPDGSSPSSSSSPSSSSSKALVHNFI
LNQDQDTTPTGNNNTGSLLNNHPHHFHQFPIDGSSKNPFFEDSTTCTDPFIDPYTNDLN
AYIPSLSFTVPDHGTSLNNGLTFQAFSTESPCWDFSQNKASAPSETGEHHRSYQQQQLE
PMDFQDQPPTPPPPPPPPVATKVAEDASCITNQNGRNNNNQDRDDDDDDDDDDDEKNN
NNRRFLKAKRVNQATKKTSIIKGQWTPQEDRVLMQLVTRHGTKKWSQIAKMLNGRV
GKQCRERWHNHLRPDIKKDSWSEEEDMILIAAHKEIGNKWAEIAKRLPGRTENTIKNH
WNATKRRQFTRRSKAKDGNSNPPKGSLLQNYIKSVSSPTELTPAAAASSSSQHDNEFE
MTDASPHMVQPETSGLNSTGWSIGALNDHVQRQRQQQQEMNYCFDANVYNDMHR
NQSFGSMLEGDGNGNGSGMGNFELPLEMDSLKKELDLLEMISQGNL

>GrMYB024

MGRKPCCDKVGLKRGPWTIEEDHKLMNFIFNNGIPCWRHVPKLAGLLRCGKSCRLR
WINYLRPDVKRGCFTESEEDEIIRLHSQLGNRWSKIASHFPGRTDNEIKNHWNTRIRKK
LKLLGLDPVTHKPIENGEKNKQEETKSEDVRKDDEIQTTLDGTETDDLLNNYEILCGS
LDSSFSKEESNNPSMATQEEDCLKQWVDCVDSFFSWDNFTHLEEQIPFPPWL

>GrMYB025

MGQDIDRVRGPWSPEEDELLRKLVQRYGPRNWTVISTSIPGRSGKSCRLRWCNQLSPE
VEHRAFTAEEDRIIRKAHALYGNKWATIARLLNGRTDNAVKNHWNSTLKRKFIEDSES
EKKPAKSPRTASSSSPSRSDANDLGLGVVADELSQCSRVSTKLTLRSSWNESVDLNND
DDHLSKENDLKLTSEKHENDSSATVAAAMLPPEILAAAMLPPEILAAIKEMIKKEVRG
YMEEFGFRSESVKNAIGNIE

>GrMYB026

MVKKPCCDKSGLRQGTWTAEEDRKLTAYVTRYGCWNWRQLPKFAGLARCGKSCRL
RWMNYLRPNLKRGNFTKEEDETIITLHESLGNRWSAIAAMLPGRTDNEIKNHWHTNL

KKHAKHKPSTTKLHDKYTNNQNLNDDHLQINPIIPPLILESSPPPPLSTNTQSSFTTTDN
SVESTKADSVSDFWSEPFLLDILSDDLPTMGASAGFEFDLLDGEILSPFGFYDHLEPFSF

>GrMYB027

MGKPPGCDKNGLKKGPWTAEEDQKLIDYIQKHGHGRWRTLPKNAGLKRCGKSCRLR
WTNYLRPDIKRGKFSIEEDEVIIQLHSVLGNKWSAIAARLPGRTDNEIKNYWNTHIKK
KLLRMGIDPVTHAPRLDLLSSLLSSSLYNSSLNPAGFVGSMFNPSFFSLATAFLSSNQTK
NLEINNLAGNIQQIQNPYEPYQGNQVPVITQVAQADLNQFSANPGLPNLWQIDNNVSD
SKGFLPTTMQNINGYNHGFYLGDQNLQQVCTFSEKIPNLVPFGSLLSTPSSSSTPLNNG
SSTEDIERDSYCSSNNMLMFDVPKGLNVNGCM

>GrMYB028

MMMMGGNNQITTQNEGGGSNNGGGEGGIMLKKGPWTAAEDAVLADYVRTHGEGN
WNAVQKNTGLARCGKSCRLRWANHLRPNLKKGAFSPEEERIIVELHAKMGNKWARM
ATQLPGRTDNEIKNYWNTRVKRRQRQGLPLYPPEIQPLYSQHQRNQHRSLPSTPLPSPT
ATSTSSFSFQTPPNPSSSTTLHGSILTPAHPLHIPRSASSHVLYNPQTPPPLHSASAVSTPC
PSPLPSSSPSTPTPVSPLQSPHKPAFSTLPLFDSSTSNTCNNNINTNNAILQSPSDFFFPRA
TPPLQTPMRYKRFRPDVSESINNERHNNSISVNGGCSSTSSSFMSQYPPFQKTSFFNSHA
APNVTTSTPLTSPHYSPSYTLDPVTLDLASSSRILADHQNFDNGQFISTPGFDYTSKTDD
LPSNQFLSLDGTSEVTFDTKGNCNYISNDQNNHHHLSIHFGGGGLLDDMLEEAQLLA
GDNDILRRESCLDGFNTSSDGLTSGKEETVTDQEINATQEEYSRLLNAIPSSMPMPDWY
NNCGK

>GrMYB029

MGKGRAPCCDKDKVKRGPWSPQEDLRLITFIQKHGHENWRALPKQAGLLRCGKSCR
LRWINYLRPDVKRGNFSKEEEETIIRLHETLGNKWSKIASHLPGRTDNEIKNMWNTHL
KKRLAPKNGKIPQNDESKETCMVSSSCSSITFVSSPCGKRNLEVELEQQWHEGSPSKK
PREGFPVSDKAEDYKTEVPSHNSRPFEEPKEFPSSSISSSNSNITNSSQVNVPNPENHGD
SLLNFVGVCDWKNNTSEEVNKPEILNTAFDIPLESDSDFWDMLDSLGSFQPDEIQSNE
VEGNQSPDFGEEYSKENENNKWLQYLEIELGLEVTKNENHNNLSNTAAEPLVPEMYD
MLLKP

>GrMYB030

MRKPCCDKRDTNKGAWSMQEDEKLLNYIRKNGEAGCWRTLPQAAGLLRCGKSCRL
RWINYLRPDVKRGNFGEDEDDLIIKLHALLGNRWSLIAGRLPGRTDNEVKNYWNSHL
RRKLINMGIDPNKHSLTRHNRPHHNSNTNTSASATLISSASKLPKSHKCIAPPNNKPWR
DNDQASDAASCLLEDEDDDNLPCSTTQYQLPDLNLELTISVSAPTAKLEQHIHQIQEPN
HKQCSNISSDLRFSPTPTLLLFH

>GrMYB031

MGRSPCCEKAHTNKGAWTKEEDDRLIAYIRAHGEGCWRSLPKAAGLLRCGKSCRLR
WINYLRPDLKRGNFTEEEDELIIKLHSLLGNKWSLIAGRLPGRTDNEIKNYWNTHIRRK
LLSRGIDPATHRPLNEASQDVTTISFSGAKEEKEKINTNSNNNPIGFITKDEKKIPVQERC
PDLNLDLRISPPYYQQTQPESFKTGGRTLCFICSLGVKNSKDCTCSTITTAAGSSSSSSSS
HSNSNNSSGYDFLGLKSGILEYRSLEMK

>GrMYB032

MRAAMPPINTKNMSTPLEEESELRRGPWTLEEDTLLTHYIARHGEGRWNMLAKCAGL
KRTGKSCRLRWLNYLKPDIKRGNLTPQEQLLILELHSKWGNRWSKIAQHLPGRTDNEI
KNYWRTRVQKQARQLNIESNSQRFLDAVRCFWMPRLVQKVEQASPSSSSSSSSYLKE
MCTQSSVPSQLSSCCTVPSFPTVSPLANKTTDISNSSSVTTQNICPTDSINNISDQTEIPQ
HLTGSNVYGHTTLDQCYNIDSNDYGMEGISFASMSGVGFYEGTPSEGNWMCNEMSD
DLWNMDDIWQLRSI

>GrMYB033

MGRAPCCDKSNVKKGPWSPEEDAMLKDFIHKHGTGGNWISLPQKAGLRRCGKSCRL
RWLNYLRPNIKHGKFSDDEDRIICNLFASIGSRWSIIAANLPGRTDNDIKNYWNTKLKK
KLFGMVPHSYSSSSFSNITTAGSVLQSQQHRGFSANRLGRNKDSEFGYGGGSGGNHG
DQQIGNLESFFYNEGDKNDHHNLMYSSRDIANGFSSSVGSISSSFDSNFLYGETTSLDY
GIERINQQISSTSSSCEGFGFYQHNANDYPC

>GrMYB034

MGRKPCCLKEGVNRGAWSVKEDQLLSNYINLHGEGQWRTLPQKAGLNRCGKSCRLR
WMNYLRPGIKRGNISPDEEDLIIRLHRLLGNRWSLIAGRLPGRTDNEIKNYWNTILSKK
SNADVSKKTSESEDMKSVGKYLSAGISTPVVPKATRCTKIFFSSDEVDQHLKNDQCPP

LSTPTDTDDGLTLESSKDDDVPDMDNALDHVGFLQNGIADVFENSNMLESDKLAPMF
ETKFNNDLSSLLEFEDDWTNFL

>GrMYB035

MGRSPCCDENTGLKKGPWTPEEDQKLVKFINENGHGSWRALPKLAGLNRCGKSCRL
RWTNYLRPDIKRGKFSHEEEQTILNLHSILGNKWSAIASHLPGRTDNEIKNFWNTHLK
KKLIQMGIDPMTHRPRTDMFSSLPYLIALANLKDLMDQPWEEQALRLKAEAVQMANL
QSLQYLLQLTSVPTSSTSTIINTFTDMDAINNLLNPVSINKDSSNNIATAAAASLQGFND
SIPFPRLPDLQQVPSTKTNVEKLDNSSMVPNVAETLTHNLGDIACSYKEADHLSVWPH
HDDHHLIFEDPLFHELS

>GrMYB036

MMGRPPPCSRDKNGLKKGPWTPEEDRILVDYIQRHGHGSWRALPKLAGLNRCGKSC
RLRWTNYLRPDIKRGRFSEEEEHTIINLHASLGNKWSAIATHLPGRTDNEIKNFWNTHL
RKKLLQMGIDPVTHRPRTDLNILPNLPQLLAAANFTNLMMSNIPLDAAHLAKLQLLH
NIIQVLGTTPTMEALNFLAGPTFGENHEFSNLSLGFAPQELTQLQSNLLNLEAPQQHQP
EVTEYHNQPMKDSNNHQFPSLSPANIPPPLTQSQLPALVPASPERRPQTENKNINPINISN
PSSTSTTFEGWGDLMDDEESDSYWRDIIDQASSQPWPFS

>GrMYB037

MGRTPFCSSDGLKKGAWTAEEDQKLISYIQKHGEGGWRFLPQKAGLQRCGKSCRLRW
ANYLRPGIKRGDFTPEEDKTIIKLQAELGNRWAAIARHLPNRTDNEIKNYWNAHLKKR
LANMGADQVTVSGAASSSGNSDSNAVTDTECAKPQQSEPTKQRSASALLLNKLATRV
TQCVGRLRASQTLQQPTMPFNGGAESSDIFCHPLPSSTPESTSWADNNNISNSLTIPECG
TTNAIDERDSSSSAGVLNDIVASEFASPTCVDELSDWVNTNYSIEPEQVQIDYSDSMTV
GYGGLWDDDVIVVDDDDYTVGSLGFL

>GrMYB038

MERQSDGTFIKKGPWTGEEDEILMNYVKKYGPKGWSSIRSMGLLPRTGKSCRLRWV
NKLRPNLKTGCKFSAEEERVVIELQAEFGNKWAKIARHLPGRTDNDVKNFWSARRKR
LERISHTPKSKGKDHHALHEMPMVEVVPSNGVPLEQGSSSHQHPFFPGNLEEFKLVPL
PDLIKPDFLNMETGLSTLDIEPIRMIPQLQVDLPILPDSFDFNFAAMFNNQEASESESKPI
SLTKIPSAGIKGSDAELGKKENIGNSATPDSFFDEFPTDMFDCLEPLVSSSEW

>GrMYB039

MKAEQEETRKGPWTEQEDAVLVNFVHLFGDRRWDFIAKVSGLNRTGKSCRLRWVNY
LHPGLKREKMSLQEQRLVLELHAKWGNRWSRIARKLPGRTDNEIKNYWRTHMRKKA
QEKKKAMPKSLSPSSSSSSSSSSITLSSSSSTTTTTTTTTTVDSLPFSGTGKVSFYDTGGP
KMAALGDKSSTDFKDEKGYYSMDDIWKDIDMSEENMIKPLSHNYSEEGCNIFCPSMA
SPSLDYCWDSLWKMDDEDSKMFLPFSQSISCFEYGTSFFNKIG

>GrMYB040

MGHHSCCNQQKVKRGLWSPEEDEKLIRFITTHGYGCWSEVPEKAGLQRCGKSCRLR
WINYLRPDIRRGRFTPEEEKLIISLHGVVGNRWAHIASHLPGRTDNEIKNYWNSWIKKK
IRKTLVPPTQGTASITELVNQDMATTTTRAIPIPTQETLFSSQAPLFMFESTMTPIDQGVL
LQDVNVRGEVFHEQAQLNTWNNNNHLNQQLQLQVPFPPPSTSFTMDTNNNYLPPLID
NMETMVPIEVQSCTSLDEEGGGEITLESLRRQQQELNEWVESQQCSSFLLWDNIIEGQI
GGSVEGTTIPLPPSSSSSSNMGTTLSSFPSSL

>GrMYB041

MGRQPCCDKLGVKKGPWTAEEDKKLINFILTNGQCCWRAVPKLAGLRRCGKSCRLR
WTNYLRPDLKRGLLSEAEEQLVIDLHSRLGNRWSKIAARLPGRTDNEIKNHWNTHIKK
KLIKMGIDPVTHEPLNKEAKAAENHISETDGVGADSLEDNSSTPTENCSSSVGSNSLD
KFCNDESLLSSLWMNDEPPLIDASWDINIPAGGETCNGISLPSWEENCAWLLDCQDFGI
NDFGLDCLNDIELNTMNTLEMGDKTVA

>GrMYB042

MGRSPSCADDANVKKGPWTAEEDQKLVDYINKNGHGSWRIVPKQAGLNRCGKSCRL
RWTNYLRPDIKRGKFGEEEERLIINLHSVLGNKWSKIAAHLPGRTDNEIKNFWNTRIRR
KLLNLGIDPHTHKPRTDLDHLLNVSQLLCVAQLGNMMNPLDAAFKLQADAAQLAQA
QLFQNLMQIMNTNEAANVESDGFLALQENTNPIPQSIPNNGFIAGVVPQTSDQWGLSS
SFEQRENPVLPGLVSISPESNSTMENKGNNETDSTIYEAWEKLIMEDDEADGGSYWKE
MLDLASSTSSPISW

>GrMYB043

MGKQPACSSDKKGEVKRGPWTAEEDKKLIDYIQKHGHGKWRTLPKNAGLKRCGKSC
RLRWANYLRPDIKRGKFSDEEEQTIIQLHSVLGNKWSAIAARLPGRTDNEIKNYWNTR
IKKKLLKMGIDPILHNPTLHLLHLSSLLTSSLSNSYNLNNHPNKFLLGTESMSDPNFLIP
VLSSSQNKNQLQNIEKNQTENTFFQCPHNNQYEQGNQFQEYDSKGFFPMQSYGYYDC
NLSMDDQNQTCFSGNISSLSSFGSG

>GrMYB044

MRKPVADPPNMGKDNNKVKLRKGLWSPEEDEKLMKYMLSNGQGCWSDIARNAGLQ
RCGKSCRLRWINYLRPDLKRGAFCPQEEDLIIHLHSILGNRWSQIAARLPGRTDNEIKN
FWNSTLKKRLKTTTTTPTCSSLNNSDSISEQPRTDGVGAIFPVNEHDIDIMGASLSSSS
SPTSMSYPSMVTLNQFDPFPHLNNSYDMKGSFNAPTWLTTTQGGWGEGLFYGDYGKI
GLEGEFSLPPLETTAAIANNNNNSCFNNTAGEKNHGQSFKVEVDNMFGLENNHPHWH
GESLRMGEWDFEELMDNISSFPNFLDNFHC

>GrMYB045

MGRAPCCSKVGLHRGPWSPREDKLLVNYIQSHGEGKWKSLPKKAGLLRCGKSCRLR
WTNYLRPDIKRGNITPEEDDLIIRLHSLLGNRWSLIAGRLPGRTDNEIKNYWNCHLSKR
LLNQGTDPNTHKKLSQQATNTRKNISDTSKTFSERPKIHLPKPIRVTKSSFPRNQSFELD
NHQCNANTQAGGNPSSDYAYAADHGGENMRGFLADDLDIQFECESTVLVSEDDSSSL
EKLYEEYLQILNQNDDDEMQLDTFAQSLLI

>GrMYB046

MGRQPCCDKVGLKKGPWTAEEDQKLINFILTNGQCCWRAVPKLAGLLRCGKSCRLR
WTNYLRPDLKRGLLSEYEEQMVIDLHAQLGNRWSKIASHLPGRTDNEIKNHWNTHIK
KKLKKMGIDPLTHKALSSTTTEPQQSQQKNQVSKGGLKSQVVDKSPKEPETSLQSTIS
EEKSMGSPLFDPMEMMMMMDNVDGFCTDEVPLIEPHEILVPAAPSTSSSCDSSKLLEE
LQLPDFEWPSDCNTSSDDNNNKKMSLWDDDDFNNTWGDLLSDRSELALDDSLSSPLI
QCPTGMPFDQEYSWAYI

>GrMYB047

MGRAPCCSKVGLNRGPWTAREDTLLINYIQAHGEGHWRSLPKMAGLLRCGKSCRLR
WINYLRPDIKRGNITPDEDDLIIRLHSLLGNRWSLIAKRLPGRTDNEIKNYWNSHLSKR
VLNNSQTNSTTRSSRKAKKATEKKQKANNVEEKEEIKVLHQPKASRVSPFSVISRSSSF

DSLISGSSSSGEGSIGTNDAYVFDIPSYWSDFAADTNLEFPDAEAFSDATPVGDSNLLD
DIFEEYQKLLDGDDPGERDSIFDVLSCF

>GrMYB048

MGHHSCCNQQKVKRGLWSPEEDEKLIRYITTHGYGCWSEVPEKAGLQRCGKSCRLR
WINYLRPDIRRGRFTPEEEKLIISLHGVVGNRWAHIASHLPGRTDNEIKNYWNSWIKKK
IRKPSAPPTSTAPPPSTTEHSQINYGSNQLELVNQELMTRAIPNHQETLFSSPAPLFIFDTT
PLDGAQVHQHGNNVRGELFHEPANLNTESSWNLNQHQVQAFPPSNSFTVGMDTNNY
LPPLVENMENMVVPVEVQSCSIEEEGEMTLECLQRQQQELNEWVESQQCSNFLFWDT
LEGQLGGEALPPTSSNMGTTLSSFPTSL

>GrMYB049

MNVYETGFISETPQSEEDMDVKKGPWTEEEDFTLKAYVNIHGEGRWNSVARLSGLKR
TGKSCRLRWLNYLRPEVRRGNISLQEQLLILQLHSQWGNRWSKIAQHLPGRTDNEIKN
YWRTRVQKQAKQLKCDVNSKQFRDAMRYVWIPRLVEQICSSSGSHSAQQSSSTTTTT
YADTIGSVRVDPRLLPELSGTSSDSLDAQVSSVSDLTNSKYPNSLQNGSGSGNTIAGTW
GGVEIEATDGGDSMESVWNEENIWFLRQQLYDDDDLN

>GrMYB050

MDVYESGFISEMTPQSKEDMDVRKGPWTEEEDYMLKTYVNVHGEGSWNSVARLSG
LKRSGKSCRLRWLNYLRPEVIRGNISLQEQLLILQLHSRWGNRWSKIAQQLPGRTDNEI
KNYWRTRVLKQAKQLKCDVNSQEFRDTMRCVWIPRLIERICASSGSPSAQPSTTYEKT
SVLPELSGTSSDSQDGQLSSVSDLTDCYNPPSYSNYPNSLQNGWGLWSENLGGTWNIE
DGVGFQATEGEESMESVWNEENIWFLRQQLYDDKDDMN

>GrMYB051

MGRHSCCYKQKLRKGLWSPEEDEKLLRHITKYGHGCWSSVPKQAGLQRCGKSCRLR
WINYLRPDLKRGTFSQEEENLIIELHAVLGNRWSQIAAQLPGRTDNEIKNLWNSCLKK
KLRQRGIDPVTHKPLSEVENGEDNNKSQHTNSLDMVSGPSSELKLNTDNLKPGTALYE
QRPTSSITAPAPGYQLEMEGCSSSNNLMTSTTRKDLFLDRFASTATTTNGQPCDFMPHF
PVQQLSYASTNPRLSPWFSQSEFSSSSMSTLLPPLTTSPFLSSPIGFNPSVTLPSHTPSMPS
FTVNNSYAWGMADCSTSEKEEAPIHLMETQPDEMKWPEYLNNPLLMAAALQNQAPQ

SSLYNIEIKSETHCSTNTKSLSNSMWPQNQQPQQQQEGLQNSDMCAKDIQRLTTAYGH
I

>GrMYB052

MGRAPCCTKVGLHRGPWTPREDTLLINYVQTHGEGHWRSLPKKAGLLRCGKSCRLR
WMNYLRPDIKRGNISTDEDDLIIRLHSLLGNRWSLIAGRLPGRTDNEIKNYWNTYLSK
RLLSQGIDPNTHKKLPKPVVQQVSKRKNSRGSNNKQNPKKAKTTLEPAVIPKVHLPKP
SKATSIYFPRNDSFDQCNTFSTVCSSQGEEGIGTQVIQGPWSEYVSHGENGTGFLSESG
CHPRVPSSEGDNSLEKLYEEYLQFLKANEDQLQLDAFAESLLI

>GrMYB053

MGRSPCCSKVGLHRGQWTSREDTLLVNYIQAHDEGHWRSLPMKAGLLRCGKSCRLR
WMNYLRPDIKRGNISPDEDDLIIRLHSLLGNRWSLIAGRLPGRTDNEIKNYWNTHLSK
RLLSQGIDPNTHQSLPNPVVQQVKKKNNNRGSNNKQNPKKANATHEPAVIPKLHLPKP
SRITTVFLPRNDSFDQCTRFSTVSSSQRGEGALDTEAIQCPWSEYVNDGENGTGLLSYL
KCHSGLPASEDENSLEKLYEEYLQLLRTDEDQLQLDDFAESFLI

>GrMYB054

MGRTPCCDKKGLKKGPWAPEEDEILTNYIKKHGHGSWRSLPKLAGLLRCGKSCRLRW
TNYLRPDIKRGPFTLEEEKLVIQLHAILGNRWAAIAAQLPRRTDNEIKNLWNTHLKKRL
LCMGLDPQTHKPFTPCGGPTVAAPTSPATRHMAQWESARLEAEARLSKESLQTNSTPI
AKPDSDHFLRLWNSEVGESFRKINTEYKTVYPCPSPISQTSSSTKCGSVSAVTMDVCPN
IAGSLNPASNPIEETECKSFIKSCIEEPSDSSCSSESEDSSDTALQLLLDFPINNDMSFLEN
C

>GrMYB055

VSTNLLMEYMQQLPFDQGAINKMNDLHLSFDANPPKSSNLHRPQRSLGVGGFDSPW
VLCLHISSYLNSNLIGGVVIIQNDNGTHFLAEVKLGEMEGKLVEISQIRKGPWKAEEDE
VLINHVKKYGPREWSSIRSKGLLQRTGKSCRLRWVNKLRPNLKNGCKFTAEEERVVIE
LQAQFGNKWAKIATYLPGRTDNDVKNFWSSRQKRLARILQNSGTPSSSSSSSSSKSLKL
KREIPAFHDVPVFEAPNLSSSMEEISCTMAQSCSSSSYLDNTETIIKVEQFPKLVNPKLY
TDANMAQLELMSIGNNPYAAAEAQPQAFFPQTPHPQPYLTLSLESQDLLAKFEDPYFS

QVFAPMDVPELDSGNVEQQPFLEPVRSGGFGAREEADNPMIPDAFFDDFPADMFDQM
EPLPNPST

>GrMYB056

MRKPDPNMPKTNTNTTNKMKLRKGLWSPEEDEKLMKYMLTNGQGCWSDIAKNAGL
QRCGKSCRLRWINYLRPDLKRGAFSLQEEELIIHLHSILGNRWSQIAARLPGRTDNEIK
NFWNSTLKKRLKNNTSTSSSPNNSDSSEPRDVVGGIFPLHEHHAMTFCMDSISSSSTIS
MPPMGTGNQFDPFPLLNINRYDKTGGAAGLLDLPTCLTQGGLGEALYGDYGVLEPGK
IALEGDFSLPPLESRSIEESNAAINSIDRKSNNNNHHHHHNNNTCFNNTDQSFKVGEAF
GLENHHWEAENLRMGEWDLEGLMDNISSFPNFLDFQVE

>GrMYB057

MGRSPCCSSDDANLKKGPWTPEEDEKLLDYINTHGHGNWKTLPKHAGLNRCGKSCR
LRWANYLRPDIKRGRFSEEEERLIVNLHSTLGNKWSKIATYLPGRTDNEIKNFWNTHIR
KKLLNMGLDPNTHKPRTDLNHLLNLTQLICAAQLGSLMNPWESSALKVQADAAQLL
QNLIQTLNTNQLSTITAGLMGSQKSYPYEGLINGTSSLYADEPAPVPQKFHSMSQGYES
ADMSDFLISELVSESPETATTSMNQMGDDKTNTNHSSTMSPADTSMFQAWEKLMGDE
TDSSFWKDILDLTSSPSSPIQR

>GrMYB058

MGRSPCCSSDDANLNKGPWIPEEDEKLLDYINTHGHGNWKTLPKHAGLNRCGKSCR
LRWANYLRPDIKRGRFSEEEERLIISLHSTLGNKWSKIATYLPGRTDNEIKNFWNTHIRK
KLLNMGLDPNTHKPRTDLNHLLNLTQLICAAQLGSLVNPWESSAPKVQADAAQLLQN
LIQTLITNQLSTISAGLMGSRNSYPYEGLINGTSSLYADEPAPVPKKFHLMSQTSSDQHQ
GQGVIENSWASFEGYESSDISGFLISELVPESPKTATTSMNQMGDDKTNTNHGSTMSPD
SSIFQAWEKLMDDETDNSFWKDILDLASSPSSPIL

>GrMYB059

MGRSPCCAEDANLKKGPWTPEEDQKLVDCINKHGHGSWRALPKLAGLNRCGKSCRL
RWTNYLRPDIKRGKFTEEEERVIINLHAVLGNKWSRIAAHLPGRTDNEIKNYWNTQIR
KKLLNMGIDPQTHKPRTDLNHLLNLSQLLCASQLGNLMNPWDTAFKLQVDAAQLAK
TQLLQNLLKIINTNKPATVDTAGFLGSQNPYPIEGLLGGTSTLSDKELAPTPENITNNAG

ATSQTSTDFQALEDPCAYFGNGFNYESNLGMNNQSLSNSFEQTENPLPELVSASLGQT
SSINQTDNKTETAPDSTIYEAWDKLIMEDEADGSYWKEILDLTSSSSSPISW

>GrMYB060

MFLYSFTSLLLFLTNHLSLPFSLFASSDCWLIMAPKKAGVSKRVFNKGSWTAEEDRRL
AKYIEIHGAKRWKTIAIKSGLNRCGKSCRLRWLNYLRPNIKRGNISDEEEDLIIRLHKLL
GNRWSLIAGRLPGRTDNEIKNYWNSHLSKKMINHDVRTEQTSSLEQIVPYKAWETVQ
MEEEEVVKGSDEIENSEFSIDVDEFFDFSTEGCFGLDWVNKFLELDDQQDPLAMV

>GrMYB061

MGRQPCCDKLGVKKGPWTAEEDKKLINFILTNGQCCWRAVPKLAGLRRCGKSCRLR
WTNYLRPDLKRGLLSETEEQLVIDLHARLGNRWSKIAARLPGRTDNEIKNHWNTHIK
KKLIKMGIDPVTHEPLNKEARTQESSSHAHHSADNHTTENDGIANSSEDNSSSPTENCS
TTDDPNLLDAICSDESLLNSLWMDEPPLVDVSWTSISAGETNCNLTSLPSWEEDCAWL
LDCQDFGANDFGFDYLNDIELNTLEMGDKSVA

>GrMYB062

MGRAPCCDKASVKKGPWSPEEDSKLKDYIEKYGTGGNWIALPHKAGLKRCGKSCRL
RWLNYLRPNIKHGEFSEEEDMVICNLFATIGSRWSIIAAQLPGRTDNDIKNYWNTKLKK
KLMALSSQSQRKPPPLPIPSSHQTIPPSYKDCSSYYCTPSTRSFAGFEPLSTVQSDLLNH
NNTFLATDSSLNHTTPDSFVSYYPVKEKFLMFGSEPSCSSSDGSSHGKEIKQEDVSNFQ
GFCAPNGYEDNHKFMLSYGNRNQWAEKPSGYHGETPLDYDLEDVKRLIDSSSSNNSC
NNNNNNHFFIDESKRQEKVMYYYY

>GrMYB063

MGHRCCSKQKVKRGLWSPEEDDKLVKHITTHGHGSWSSVPKLAGLQRCGKSCRLRW
INYLRPDLKRGSFTAEEEQIIIDVHRILGNRWAQIAKHLPGRTDNEVKNFWNSCIKKKL
LSQGLDPKTHNLLSSRQRASNKLACKYSSSSPSSQSQHGSFTVFNITSHAKDTNNNNN
TTTTTMMMNPPPPVVTFPHQSPNPNTTYAQNLYGSSMDTPFVSSSSCFGNMLYESDPCI
WDDANAVVETFEEPRVDSLPPAPQPQQQENDDKIDMDCSLMEGGAGSFDLGLLESTL
LCAAMDDFGWNF

>GrMYB064

MAGKGRAPCCDKEKVKRGPWSPSEDLRLITFIQKHGHQNWRALPKQAGLLRCGKSC
RLRWINYLRPDVKRGNFTQDEEDTIIRVHATLGNKWSKIASYLPGRTDNEIKNVWNTH
LKKRLGSMNGNADKKDESMATSSSSSCVTSMSSFNDKKTNEAFEMAVVSDKANHGS
PSEEPEALSSSSISSNVTNSNQVNVSNPEIQEGPLFNFTGCYYNTSEEVNKPETAFDIPLE
SDLEFWNMLDSLVPFQPEGIQSHNETQCPDFGEAENKWLLYLEQELGLDPKDGGFEA
EPLLVPETNGMGTMGHYHNTVQPYEPNSKQ

>GrMYB065

MSWAMMAGHLGWGFIEEGWRKGPWTAEEDKLLIEYVKLHGEGRWNSVARLAGLKR
NGKSCRLRWVNYLRPDLKRGQITPHEESIIIELHARWGNRWSTIARSLPGRTDNEIKNY
WRTHFKKKNKLPSDNNTSDHKSKARLLKRQQFQQQLLQQQQQLQLQQQQEQHE
QQLQLNQLDMKRIMSLLDETEHKSSVLPYVPQLRQEMATAVPYPNTTVEQQQAGLFY
PMMEFDGNVSGSGSDTSNEVDVLWDGLWNLDDINGNFGATSKVSFHNLATPFS

>GrMYB066

MDGKKRGCTGNVQSEEDHEMDLIRGPWTVEEDFKLINYIAIHGEGRWNSLARCAGL
KRTGKSCRLRWLNYLRPDIRRGNITLEEQLLILELHSRWGNRWSKIAQHLPGRTDNEIK
NYWRTRVQKHAKQLKCDVNSKQFKDTMRYLWMPRLVERIQAAAGSTTTTTTTTAVV
PSASVMGKDFSTHVTPSYTSENSSTATAASSESFEAQVSPVSDLTNCYNNGFQVTNNN
NPNPDYNFQACSSQVDYYEHSLSAANYYNHGDGGCLDLQTNNYPMLDGADLSDNL
LNAEDFLFLQQQFNFNM

>GrMYB067

MNRLSCHSSTTPSSSSSSMGMVYPDMGCLSLPPNYGIVVCSSRENERASSGFRFMENS
KTRSFEENHSSDAVEGKGSDYSDGFGDNNMPINLNSNLNEENPNENAVSGKETDSGQS
KLCARGHWRPAEDTKLKELVALYGPQNWNLIAEKLEGRSGKIVAKNSGKRKSKSCRL
RWFNQLDPRINRRAFTEEEEERLMQAHRLYGNKWAMIARLFPGRTDNAVKNHWHVI
MARKYREKSSAYRRRKLSQSVYRKMEETPNIVCRDAVTKAEPPPYCLNMSDRRLGIIS
HYQFGIFNGGDAGGVNGGSNVSPHMTSEREAILSSEASYRNGFCAQAQQPPFDFFPDL
SNDMMGTFSQTRFWDRPIDEPQVRGYYPQLQHQQLHPSHMMTMQQSEFLSSQSNTE
STASITPQVSAIESSSSVAGSSNYSHYETILPPFIDFLGVGAT

>GrMYB068

MRKPCCDKQDTNKGAWSKQEDEKLINYIRLHGEGCWRTLPQAAGLLRCGKSCRLRW
INYLRPDLKRGNFTEDEEDLIIKLHALLGNRWSLIAGRLPGRTDNEVKNYWNSHLRRK
LINMGIDPNNHRLITHNHLPRPHDSNSGASIISPASKVPVTDNSNIHPPEKSRGDNDQVS
DAASSLEDHQLPDLNLDLTISVSASTVDKLKMIDEKARNSPT

>GrMYB069

MVPILFPTLYIYICVEVYDSFRIDELHINFSLFSVKRSPLSLTPSRKLQHTSKLNLSKGKP
MGRTPCCDKSGLKKGPWTPEEDLKLTNYIQVHGPGNWRTLPKNAGLQRCGKSCRLR
WTNYLRPDIRRGRFSFEEEETIIQLHGILGNKWSAIAGQLPGRTDNEIKNYWNTHIRKR
LLRNGIDPVTHAPRLDLLDLSSIISSTLCNQSLLNVSNLLGTQALLNPQLLSLANTLLSL
KQENPEMLMQYLHQNQLNLDPSLQAPIQIASACTTTSTVPCCTSMLSQTGLIQATGEG
FFSSDMTNFSYPNSQENLTNSTLTNEFAAQPHYVQCSTNPTVPILSETSKFQSVDGSFDS
VRSSAISSPTALNSSSTFVNSSSTDDERESFSSLLKFEIPEGLDINDFM

>GrMYB070

MVRTPCCDKTGLRKGTWTPEEDRKLMAYVTRYGCWNWRQLPKFAGLARCGKSCRL
RWMNYLRPNIKRGSYSKEEEETIIRLHDSLGNRWSAIAAQLPGRTDNEVKNHWHTTL
KRRFKHKPSAAAKDGKDNSSQDPKLRRGTSREKEETKLNDVDHLLISSPNPPLILESSL
SSQQPASSDQNSSITTDNTVVTSKLDSGSDDNSTTTNTNTDIHTDTLLEAYEAESSNFW
TEPFFSDINYMWSDVSSVVPYEADQFPLLDGEILYPFDFYDQLQGLDL

>GrMYB071

MDQDIDRVKGPWGPEEDESLRKLVQLHGPRNWSLISRSIPGRSGKSCRLRWCNQLSPE
VAHRPFTAEEDEIIIKAHAKFGNKWATIARLLNGRTDNAVKNHWNSTLKRRRSAEDGD
SVQESERRSSKFPRSGSISNPGSPTGSDVSDLGLSVAESTPHSRVSTALTLGRSWSDELV
ELNNDNSCTDKESGSVAEKHDNGTKTASLGTELLAAMQEMIRKEVRDCMAEFGFHN
QCWG

>GrMYB072

MGRSPCCEKAHTNKGAWTKEEDDRLIAYIRAHGEGCWRSLPKAAGLLRCGKSCRLR
WINYLRPDLKRGNFTEDEDELIIKLHSLLGNKWSLIAARLPGRTDNEIKNYWNTHIRR
KLLNRGVDPLTHKQLNESTARHVANAICCNGVNYEGNKEDSNYDIKNPNEYIHKDDD

DEMKKIPDLNLDLRISPPTEPIMSSSMNKSKSKSKNNGNGYDFLGLESGFLDFRSLEM
K

>GrMYB073

MKMTLQQFHNSSCFHHQENNGFSKYYTIYQPPSNSSMEVLKPIEGFSYCLEDHFSKGS
EMGAVSMSVVRGRSEFFLNGKDVGVSKICSRGHWKPAEDSKLKELVTLYGPQNWNLI
AEKFQGRTGKSCRLRWFNHLDPRINKKAFTEEEEERLMAAHRAYGNKWAMIARLFPG
RTDNAVKNHWHVIMSRKYREQASAYRRRKLSQAAIGDFTDSTQNPYLASFVGPNGSY
NKGSTSRKEDEINGGDLFLGSKSKSFKACENSTPLDFFSDYRSQEGLISRNSRQWKMV
QEETMLMQESCNHYQPSGFSDSMASASQVTVTEPSSSSVSHLETTITAPSAAAAAAAA
SPPFIDFLGVGA

>GrMYB074

MGRPPCCDKAGLKKGPWTPEEDIILVSYIQQHGPGNWRAVPTNTGLLRCSKSCRLRW
TNYLRPGIKRGNFTDHEEKLIIHLQALLGNRWAAIASYLPQRTDNDIKNYWNTHLKKK
LKKINGSESYSREGFSSEKHQISRGQWERKLQTDIQMAKQALSDALSPDKSSGLASKP
RGYASSTENIAKLLKKWMRNPPKPASSDNMGGIGTPWKENKSSNSIEMSEVFESLDVF
ESFDSSNSDFSQSLSPDQARLFQDESKPDVNELGQLTLLEKWLFDDGANQGKDDQLS
DIKLDGNAIFF

>GrMYB075

MDKKPCKSQDVEVRKGPWTMEEDLILINYIATHGEGVWNNLAKAAGLKRTGKSCRL
RWLNYLRPDVRRGNITPEEQLLIMELHAKWGNRWSKIAKHLPGRTDNEIKNYWRTRI
QKHINKQAETFSFHISTPELLDNQASTSQLPCNGSTSFMETYSPMSSFNHCNLEPFPGQ
QQEVQQQQQQQPQQQQIVDQPNNESYWSMEDFWSMQLLNGD

>GrMYB076

MGRQPCCDKVGLKRGPWTIEEDHKLMNFILNNGIHCWRTVPKLAGLLRCGKSCRLR
WINYLRPDLKRGAFTESEEDQIIQLHGRLGNRWSKIASHFPGRTDNEIKNHWNTRIKK
KLKLLGLDPVTHKPIEKVEKNDGDSSNKSNGDDQQQGIELDDDEKVEGIELSLDETND
LFNSYQMLCESFDLDSWLNQDAANNTCSSSYSMEESSNKSSTGETNSTIGEDSLKQW
VDSVDSFLSWDSFI

>GrMYB077

MSSFTKASSSTSCSEEDTEQLRKGPWTLEEDSLLTRYVARHGEGHWNLLAKLAGLRRT
GKSCRLRWLNYLKPDVKRGNLTPEEQFLILELHCKWGNRWSKIAQELPGRTDNEIKN
YWRTRVQKQARHLKIDANSAAFRNVIRCFWMPRLLQMMKGSSTLPQQVAGLGQQNS
DSEQCASSCVSSSESMNNNMSKISEFAEYQTSSFGITDNNEYNTVAKDCYYVDNSSCY
GMETINLPSTSAVGDFMTADCHMVDNNWVNDGFADGIWSMGELWELPLKGNL

>GrMYB078

MGRQPCCDKLGVKKGPWTAEEDRKLVNFIVTHGQCCWRSLPKLAGLRRCGKSCRLR
WTNYLRPDLKRGLLNQDEEQLVIDLHARLGNRWSKIAARFPGRTDNEIKNHWNTHIK
KKLIKMGIDPVTHEPLQKPDTQHEPSYSHGANDYHPNLDKNQLELPKICANVSSTAAD
VLRTSSETGSDEDPLMNLIWSDAFLNDLSWNLTGLDSWEECSEVGLFSSELLENGNAI
DGYHSFGFGGFTEIDFNSLDMGAKF

>GrMYB079

MSWAMMGEQFGWGVIEEEAWRKGPWTAEEDRLLTEYVRLHGEGRWNSVARLAGLK
RNGKSCRLRWVNYLRPDLKRGQITPHEESIILELHARWGNRWSTIARSLPGRTDNEIKN
YWRTHFKKKAKLSPDNSDQAKARTLKRQHFQQQLLQQKQQQQYLQLNESDMKRM
MSLLEETEPKAPFMPQLRQEMGAATSCPNMVEEQSLFYPMGNGNETSNEDVVWDGL
WNLDDYQWKLWCSKPS

>GrMYB080

MDVKARSSSGPNMVSTEEEDLKRGPWTIEEDFKLINYISIHGKGRWNSLAHSAGLKRT
GKSCRLRWLNYLRPDVRRGNITLEEQLLILQLHSRWGNRWSKIAQFLPGRTDNEIKNY
WRTRVQKHAKQLKCDVNSKEFKDTMRYLWMPRLVERIQAATTTTNTASTSAAMTSN
ASIEQMMLPNLSYYTPDNASTTASSDSIGTQVSPISDSIDYYNGVSTNYNPNHDYLQAN
HESLISPIGSYCNNGDGMDFGLMELNHHWASDGDGDGSHNLLNAEDFFFLQHQFNFN
M

>GrMYB081

MRKGQSDFSLVEEAGRRGNMRESCHLKKGPWTSAEDAILVDYVKKHGEGNWNAVQ
KHSGLSRCGKSCRLRWANHLRPDLKKGMFTPEEERRIVELHAKLGNKWARMAAELP

GRTDNEIKNFWNTRIKRLQRAGLPIYPPDVCLQVNLSQEESHNVASMPNQDSFGSNLV
QSDAFGIPHVEFENLELNQQFLSYSPELLNIPPKTIGLSHGYGHVFPMACPPKHVQESV
SNYMSLPNQMTDFACKENFDEPYKSSSPYDSDFSTNDQSSFGIHPGSDALLNSNFSPSE
PLSGPMNLELPSYQYSDNQQDSWVNSSVPLALVESVDTLIQSPPMKRAKSECFALQNS
GLLEAVLYESKKLNSSKDDSRQQSSNLMLDDVADFHLKDCELEFEAHCDPNSPSALSA
ASVLSEHTPVCGSSLDDESQFVESILGGNGKNETPNQMAISGLDDFLGRGQFGHDNGC
VNDKPIVTDVIAALLGEDACCGY

>GrMYB082

MMRKPPSMKGNNSNGTNKHKKGLWSPEEDDKLVTYMLTNGRGCWSDVARNAGLQ
RCGKSCRLRWINYLRPDLKRGAFSPQEQELIVHLHSILGNRWSQIAARLPGRTDNEIKN
FWNSTIKKRLKHSSSTASHNASDSSSEPNKDAMAAGFMTMLEQEVPPIYLDLSSAWSN
SFLQSMVLNHSGNSLPMLQHGRNVVGAVGYFDPAGSCVTQAEVNGDSSLGESEIFGS
VDNGIERELYVPPLESIGKDLKTENSVDGNINNGFNIINTSGVRSNNNNNMSKNMDSD
DVGSFWIGEELKVGEWDMENLMKDVSSFPFLDFQS

>GrMYB083

MGRAPCCDKANVKKGPWSPEEDAKLKEYIQQHGTGGNWIALPQKAGLKRCGKSCR
LRWLNYLRPNIKHGEFSDDEDRIICTLFASIGSRWSIIASQLPGRTDNDIKNYWNTKLKK
KLFGMVPQSQRKPHQIPHASFSSVLQSSSPSSPSPSSPLLYNCSNNTYHTPARSFSCFEA
SYSSSFSNSSASCVNAASVLQPQESLLGQIQHYQLHDNGVQMFGGEASCSSSDGSCSN
QISHNKELENSNGTVYDGDHQQFGMYSYNNFYSEAADGSQKLMIALNGDHGTDGW
SDHHKSSNGLLDETETPLDYGIEGIKQLISTTSTSSCNSFIFDENKADENLLYY

>GrMYB084

MTELKIEERCLENKQLTTASSSSVSEGGGSAIVKSPATSSPAPTSPNHRRTTGPIRRAKG
GWTPEEDETLRNAVATFKGKSWKKIAEFFPDRSEVQCLHRWQKVLNPDLVKGPWTQE
EDDKIVELVSKYGPTKWSVIAKSLPGRIGKQCRERWHNHLNPDIKKDAWTLEEELAL
MNAHRIYGNKWAEIAKVLPGRTDNAIKNHWNSSMKKKLDFYLATGKLPPVVKIVLK
NEIKDIETPAATENLLVCSKKESDSTAQTSSGTFDICKPEEDGKDQLESSTPVQDMAATS
SIIPSESVETESAEFKPQSFDANPCSCNSVSMVKFESRRTSSAIVEDKVVETQLRSDTPT
YGPLCYEPPRLQGGTSLDSDNLNKKGMQHEWTITPITSPLSFFTPPCVKGSGFGTQSPE
SILRIAAKTFPSTPSIFRKRKTSPNKIGKLNEETTKNRVQLSGEQERTENSREQAQFRDG

SACESPAFQGNSSIGPNCTAFNASPPYRLRSKRTAVFKSVERQLEFAFDKERHEDSMKS
SNLLVNGSSSIEDCLHATKMEVT

>GrMYB085

METEVEAVDNGNGTTVAEINVPSPPPLDGGEGGDCGSRDDSAAGGRTRKQRVKGPWS
PEEDAILSELVSKFGARNWSLIARGIAGRSGKSCRLRWCNQLDPAVKRKPFTDEEDRMI
ISAHAVHGNKWAVIARLLPGRTDNAIKNHWNSTLRRRYMDLGRINTESANMMEDAG
CLDKTKASSEETLSCAEDSSFRSLEWKDASSSEKLDNQSKGKALAGTQSNHELIEPPTL
FRPVARISAFNVYNNMDAPEFVSPCSRPVPVLGPSIQTSKPDVGTCKLLEGVYTDRLVP
LHCGHGCCGTQNGQNKSLLGPEFVEFSEPPSFPNYELAAIATDISNLAWLKSGLENNSV
RAMDDATAGTSHRSQVHMGHFEESQMSDRLRIEERNSKFMGTMTNLLST

>GrMYB086

MQSVTNPTAGSRKSSHGSWGQTQTLVYIYILLVSLFISPPLPLLWSVAFACLNSLPLGLQ
MRKPCCEKKDTSKGAWSKLEDQKLTDYILKHGEGCWRSLPQAAGLLRCGKSCRLRW
VNYLRPDLKRGNFGEDEEDLIIKLHALLGNRWSLIAGRLPGRTENEVKNHWNIHLRK
KLLMMGIDPNNRRSLLSPTLTKSNASNDSSSACQPFNPATASETISSTCLPDLNLDLTIAP
PLSHQNLALN

>GrMYB087

MVGVDWCKVRGMGRQPCCDKLGVKKGPWTAEEDKKLVNFMRTHGYYCWRAVPKL
AGLRRCGKSCRLRWTNYLRPDLKRGLLNHDEEQLVIDLHARLGNRWSKIAARLPGRT
DNEIKNHWNTHIKKKLIKMGIDPVTHEPLQKPDTPQNKPNLDPNQPENLSKSCAHEFS
LPTGPDKWRSSWESDDDPSINLIWPEAFLHDLSENFHGTDSWWKEYSEIGTSSKEETC
FSWLVGDAGEQ

>GrMYB088

MSWALMARHFGWSIMEESWRKGPWTAEEDKLLIEYVKLHGEGRWNSVAKLAGLKR
NGKSCRLRWVNYLRPDLKRGQITPHEESIILELHARWGNRWSTIARSLPGRTDNEIKN
YWRTHFKKKPDNSIKAKARILKRQQFQQQQILQQQQQQYQQQQNQLDMKRIMSLLD
ETEHKVPYVPQMSRQETMGTTNSYLNHNSTTDQEQGFVYPMIIDGNVSSSDTSNEEFL
WDGLWNLDDDFNGNFSAACATSKASLHNLATPFC

>GrMYB089

MDVKAKGNVRREEEDHQIELRRGPWTVEEDLKLVNYIAAHGEGRWNSLALFAGLKR
TGKSCRLRWLNYLRPDVRRGNITLEEQLLILELHSHWGNRWSKIAQHLPGRTDNEIKN
YWRTRVQKHAKQLKCDVNSKQFKDTMRYLWMPRLVERIQAGIASSSSTTATSTGQIVI
PQVALSYNTHDNSSTAISSETQVSPTSDFSDCYGNMVPINENPNPYYFQASQIGSSNDY
NHGMDFQPWLRGVDNITSDHFLDCDDFLLQQQFNFNM

>GrMYB090

MEEKSEYKKGLWSAEEDSILMEYVRKHGKGKWNRIPKVTGLKRCGKSCRLRWINYL
SPGVKRGRFSDQENDLIIRLHNLLGNRWSLIAGRVPGRTDNQVKNHWNTHLSKKLGII
KNPTAHSSQSQTPSKSSRDDDNSHSQAQDQVMMTTTDALPSPRDYGNGSSSWFWQC
HNNDHFNLHTPTPVNVLDLLDHDHRVSLDFVWHGFA

>GrMYB091

MGQAYLRFFIANQQMCSRGHWRPAEDDKLLDLVQRYGPHNWNAIAEKLQGRSGKSC
RLRWFNQLDPRINRSPFTEEEEERLLASHRIHGNRWSVIARLFPGRTDNAVKNHWHVI
MARRCRRQRSSSKTLPPSTSTSTSTSHQHHITSTTLFPHNNYMHAFPPYSYFKHFYAHN
PNPSLTTTTQAIADTTQPTEFYDFLQVNTDSNESEVTDNTCKRDGEEVDQEHQSRPAVP
FFDFLSVPRKLYTIHKRRLR

>GrMYB092

MGSKSSDQKPRAKHRKGLWSPEEDLKLRNYVLKHGHGCWTSVAINAGLQRNGKSCR
LRWINYLRPGLKRGMFSPQEEDTILTLHHLLGNRWSQIAQNLPGRTDNEIKNYWHSHL
KKRVAKAEELEPQTRTPYTTSSSENTDSALSPRNFADMRIPSFESFHHIEKPSISTDQTVA
RQLNISNEPHGSLMPRLLFAEWLSIDQEGESVANSASLNGFNHGSSSNFQDPFLSAYLG
SFDNDFQDGLSYALVNDEMFSSEFKFETQISEEEFLGNEINNNVLYI

>GrMYB093

MGRPPCCDKIGVKKGPWTPEEDIILVSYIQEHGPGNWRSVPTNTGLLRCSKSCRLRWT
NYLRPGIKRGNFTEEEEKTIIHLQALLGNRWAAIASYLPQRTDNDIKNYWNTHLKKKL
KKGQNQDGVSYCQSVPKGQWERRLQTDIRMAKQALTEALSLRKQNPSTGLNDFNDL
LNSAQPHQPPTYASSADNISRLLQNWMKNTPKPASATATTNSAETMTRSSFNSNDEGA

LSDKGLDSFFSFNSCSTCSDNDVSHESENSVVFQVESKPNMGDQIPLTLIEKWLLDDVL
F

>GrMYB094

MAVTSTSNKKEMDRIKGPWSPEEDDLLQKLVQKYGPRNWSLVSKSIPGRSGKSCRLR
WCNQLSPQVEHRAFTPEEDETIIRAHARFGNKWATIARLLNGRTDNAIKNHWNSTLKR
KCLSVGEESYFITHGGYDGNLGGEGEQQPLKRSVSAGLYMSPGSPSGSDLSDSSVPVL
SSSHVYKPIPRTGGVNVDVNVMPLPGAEAAASSSNDPPTSLSLSLPGAESYELSVSTLP
VTESTQTRNEAKNDGKGGGVMGFSAEFMAVMQEMIRMEVRDYMVQMQQQNGGVS
GGEGMGMCLDAGFRNVLSMSRIGVNKID

>GrMYB095

MGRQPCCDKVGLKRGPWTIEEDHKLTNFLLNNGIICWRKVPKLAGLLRCGKSCRLRW
INYLRPGLKRGSFTDSEEHQIIQLHSRLGNRWSKIASHFPGRTDNEIKNHWNTRIKKKL
KQLGLDSQKPFEIEKSQKNYGNKNNMNSDSSSSNNQEAISLESRPEDDHGMKICPKKA
EKPEEMKASVDETNDPLNNYQMLCGSSDLDWLKESNNVSMGETACVEEEYWVDGV
DSFLSWDSFVQVEDKFFPSWEIGN

>GrMYB096

MGRHSCCYKQKLRKGLWSPEEDEKLLRHITKYGHGCWSSVPKQAGLQRCGKSCRLR
WINYLRPDLKRGTFSQEEENLIIELHAVLGNRWSQIAAQLPGRTDNEIKNLWNSCLKK
KLRQRGIDPVTHKPLSEVEHGEDKSQPRTNSKDMASSELNINTNNPKPGTSPVTPRGY
QLEMEGSPTSKTVNSSDNNGNTTTNLMVSTTTASKGFFLDKFSTNSQPSDLVGHLPVH
QLNYASNARLSSTSNPTPWFTQTTQAFDINSEFSSAAMSSFLPPLTASFLSASMGYKPSV
ADSPSMASFTVNGSRYWETGASANNNSNSSSNSTELQSNSSFYDNNSSSWGLPDCSTP
EKEAPLHLMESQAEDIKWSEYLNNPLLMAAALQNQTPQSLYNMDIKSETHFLTNNSS
NSMWTHNQQQQQEPLQNPNMCAAKDIHRLTAAYGHV

>GrMYB097

MDVYKRGFISETSRSEEEMELRKGPWTEEEDSMLRAYVNIHGEGRWNAVARLSGLRR
TGKSCRLRWLNYMRPEIKRGNISLEEQLLILELHSRWGNRWSKIAQHLPGRTDNEIKN
YWRTRVQKQAKQLNCDVNSKQFKDAMRYVWIPRLVERIRASSESPSSQPSTTTNTTY
NDRISNISSSQMSYANASGSVQVDPSLLPELSGTSSDSLDTQVSSVSDLTDCYNPQSLSN

YLHKGLGLEKEGAATWGRDEEFQATEEHSNGWLVGGGESSMDTVWNEENVWFLQQ
QLHDGI

>GrMYB098

MGRAPCCEKVGLKKGRWTAEEDEILTKYIQTHGEGSWRLLPKNAGLLRCGKSCRLR
WINYLRADLKRGNFTGEEDELLIKLHQSLGNRWSLIASHLPGRTDNEIKNYWNSNLST
KMYCFRRLSNQSLPVILNVTGRKGMKDNRGHSTKKENNGNSSSNKPMEVFIDEVVPF
LSTPSLEKETFPSAVEDSMHLDPYEEDNERRIGVIPSSCQHTGDGDRYVENSMLCPAAT
DSVVEKETDIFSLFGSTESSGMLCFDNILDNELLQTNGDSTLSRWGLNQVSVDLENSG
DLCPNKPAAINKQVESGDCGTGDLTSYFCTTSCFFDDCEVNNILDWDWERNELWDEN
ECIMPSWLWELDYNDKDDRLTLENNDLELHGSAVA

>GrMYB099

MVRAPCCEKMGLKKGPWTPEEDQILINYIQLHGHGNWRALPKQAGLLRCGKSCRLR
WTNYLRPDIKRGNFTREEEDTIINLHEMLGNRWSAIAARLPGRTDNEIKNVWHTHLK
KRLKHSHGSNANNRQPIDPSKDIKREQQPVTIYSQVSPPQSSSDVSTSENNSNTNTSTT
KTETNEDVSEIDENFWSEVLSADNSSMEANFRVVGSDQYFPSSPPPSLPALETVNGYGS
NLYDTDANMDFWYILFTRAADLPELPEF

>GrMYB100

MCFPMSPSSYFLSPWLVAQKVRESFTFDFETPMRKPCCDKQGTNKGAWSKQEDQKLI
DYIRIHGEGCWRSLPKAAGLHRCGKSCRLRWINYLRPDIKRGNFAQDEEDLIIKLHAL
LGNRWSLIAGRLPGRTDNEVKNYWNSHIKRKLMKMGIDPNNHKLNQYPHHVGPLNP
TTTNSMDVACKLRVFSTENDDGVSDAASYLEDETPPTGISNLDLDLTIAFPSSPIKNIIEE
SQKKTASIVTNDEEEQYTVPTLLLFR

>GrMYB101

MGRSPCCEKGHTYKGSWTREEDERLIAYIQAHGEGCWRSLPKAAGLLRCGKSCRLR
WINYLRPDVKRGNFTDEEDELIIKLHSLLGNKWSIIAGRLPGRTDNEIKNHWNTHIKRK
LVSRGIDPLTHRPVNEQAEIHTIYTVSSTAVLREDGRQKNQELNLELQISPPSLHSHPPQ
VLQKRNRKVICFYCSLGIRNSKECTCEGSSHSRSIQHVSFCTWRKAFQSEN

>GrMYB102

MDVTSTPDRKEMDRIKGPWSPEEDELLQQLVQKHGPRNWSLISKSIPGRSGKSCRLRW
CNQLSPQVEHRAFTPEEDETIIRAHARFGNKWATIARLLNGRTDNAIKNHWNSTLKRK
CLPVGEECNFVANGGYDGNLGGEERQPLKRSVSAGLYMSPGSPSGSDVSDSSVPVLSS
SHVYKPIPRTGGVNVDVNVTPAGVEAASSSNDPPTSLSLSLPGVESCEVVSTQPITEST
QNRSEERGGGVMGFSVEFMAVMQEMIRVEVRNYMTQMQQQQQQNGAVSGGAGM
GMCLDGGFRNLMAVNRVGVSKIE

>GrMYB103

MGRAPCCDKANVKKGPWSPEEDAKLKAYIEENGTGGNWIALPQKIGLKRCGKSCRL
RWLNYLRPNIKHGAFSEEEDNIICSLYINIGSRWSIIAAQLPGRTDNDIKNYWNTRLKK
KLLGKQRKEQQARRASWLTVKQEMKREGGDYMLPGMVSQLPYWPQQPPVIPFTTTT
NQDPPLPNDQESIKNLLIKLGGRFSDDHHPQSSTSTPIPNSMNSSRYPLDVSFAQDQLYE
NSMNNTVSPASSISPINSTCSQVTNGTHFNTNEVAVPNDMFQGLDGFTDELRELTYSNN
QQIMNWLEGFYGTDNMVDGSSTTGSSSVESSSWGPIYSLGFPQLVTGFEPCQQNMPQ
ASTYGEQYCTWAV

>GrMYB104

MGRSPCCDENGLKKGPWTLEEDQKLVKYIKENGHGSWRALPKLAGLNRCGKSCRLR
WTNYLRPDIKRGKFSQEEEQTILNLHSILGNKWSAIASHLPGRTDNEIKNFWNTHLRK
KLIQMGIDPMTHRPRIDIFSSLPHLIALANLKELMDQQQPWEEAAQMAKLQCLQYLLQ
NPPSISSPSNNMHTTFTDMETMDISNSLLSSPPQLASVQGLDDSIPFSRLPDLQIPCDYRI
SSSSKDDTAQAQAQKHAMLSQGENTCNSTWLPSPSAVPSVTETLISNLGDASCSSNFD
VAAPSVWLDDHQLFQDPLFHNLL

>GrMYB105

MGRPPCCDKVGIKKGPWTPEEDIILVSYIQQHGAGNWRSVPTNTGLLRCSKSCRLRWT
NYLRPGIKRGNFTPHEEGMIIHLQALLGNKWAAIASYLPQRTDNDIKNYWNTHLKKK
LNKFQSALDPHPSIERKSSLEFASTSSSTSAVGLNQASSTYASSTENISRLLQGWMRSSP
KTTNDNNSSLLLKDTSQYQKWDQNTSLLDADSSAGYKPKAEQEGGNLISHEEFESILS
FENINNVVWDKSTCESTSKGACQDSGNDDDGDDDDDDGDDKVNVAMAPENYKKQK
VDHHNNNNNNKNNPPLSFLEKWLFDESSSGQVEEMNQMMELSSVF

>GrMYB106

MSLPNLNNDFDCCSGVVKEMNFLAPPPILSPSVERDSTNPQMGFQIPSSLMEQHNKCL
SFEPLESRGTMRAAACYGKDGVLGDEKKGLALNLGVEVDDVSNKNSGAVGGTKIGH
TKLCSRGHWRPAEDAKLKELVAQYGAQNWNLIAEHLEGRSGKSCRLRWFNQLDPRIN
RRAFSDEEEERLLSAHRVYGNKWAMISRLFPGRTDNAVKNHWHVIMARKHREQSSFY
RRRKPCSSSFASQAVPKGLDVTFQNNACSDQSTISSNIDESASTCTDLCLTPSPAEVPLG
FFSSFSPVLQTGPSRTKENGDFGKLYGSGNVLYQQQGPMGVANGVDQSTHSSDSEVTA
GEPVGTNKTNHSIESEDGNEKKIHNMRFIDFLGVGAS

>GrMYB107

MKNPSLVGNSTSSKGTPCCSKVGIKRGPWTPEEDEVLANYIKREGEGRWRTLPKRAG
LLRCGKSCRLRWMNYLRPSVKRGRIAPDEEDLILRLHRLLGNRWSLIAGRIPGRTDNEI
KNYWNTHLSKKLISQGIDPRTHKPLNLITHKSPQINHDPVPKSNPSPPIPNPSVLASKTIT
IKTTNTTKDGTPTNLEHGYQQQSQNQQVDKSMEKRHYTESATLIMGEPSSHGNEGDH
MENCNEDMFSSFLDSLINENLLVNQHPVEQQPNNLVAPAAAAAESSQNLSHGDMWET
ELISAMVGFGNEPNSFNNHYHYLHQL

>GrMYB108

MGGVAWTEEEDQLLKKCIERYGEGKWHRVPLLAGLNRCRKSCRLRWLNYLRPNIKR
GTFAEDEVQLIIKLHKLLGNKWSLIAGRLPGRTANDVKNYWNCHLSKKLNNAQETEE
EYQNGMKMETLKPQPRCIAVTGSIKPRTQDHFAPKNQPTIQESTMPTPFNFVEVDKQQ
GQELVKEEEDIREEAAGGVFFGDLATEDHQFGQLDEVNVLSSTEGCSKWDWDDLML
DMDLWTDSL

>GrMYB109

MMMMMGGNNQFTAQNEGGEFLGSNGMNSGGVGGGREGEIPLKKGPWTAIEDAVLV
EYVRSHGEGSWNAVQKNTGLARCGKSCRLRWVNHLRPNLKKGSFSPEEEKIIIELHAK
MGNKWARMATQLPGRTDNEIKNYWNTRVKRRQRQGLPLYPPHVQPFYSQHQHRQR
HSHPPPTTAPNSCFSFQTPIVSPHTSNPMPLHPLHIPHRPPPQNFLYNPHSALTTPPPPLQS
PNSASTPPPLPSPSASTPHHISPLHSPHNPPPFPTLPLINYPNTTADDDFFHSNKRFKHDG
LQSNNYNNNHLDATSSRFTLPFSPMQHYSNRMTLDLPSSSRTTFNHHPHQDSGDFYPL
KMDLRSNQINDDGLLENMLQEAQALAANGNGGNNEIPKEMMYAAQNEEDYSRLINI
DGPSSFGTAIPEWCNDNGESSERQPSAIIDNENHLALDMHHIASLHPADISPNHAARSSS
VRSWDNFPALEQSRFLVKI

>GrMYB110

MMMMMMMRENNQFAAQNEGGGSLGNNGRNNGGVGVGREGEIVLKKGPWTATEDA
VLAGYVRRHGEGNWNAVRKNTGLARCGKSCRLRWANHLRPNLKKGSFSPEEERIIIE
LHAKMGNRWARMATQLPGRTDNEIKNYWNTRVKRRQRQGLPLYPPDVQPHYSEHQH
RQRHSHPSSPIPSPPPTTEPNSCFSFQTPIVSPHTSNPMPLHPLHIPHRPPPQNFLYNPHSA
LTTPPPPLQSPNSASTPAPLPSPNASTPPHISPLYSPHNPPPFPTLPLFDYPNITTDDDFFHS
NKRFKHDGLQSNNYYNNHLDATSSSFTLPFSPMQHYSNGMTLDLPSSSRTAFNQDSGC
FYPLKTDLRSNQINYDGMLENILQEARELAANGRGGNKEMPKEMMNSAQNEEEDYS
RLINIDGLSSLGMAIPEWCNDSGESSERQPSVITDNENHLALDMHQIASLYPADISPNHA
ARSSSVRSWDKFPGLC

>GrMYB111

MSPLNLSNDFNCYNGGEVQERNFLSLPSIFPHQIGRDTKNSEMGFQIPASLMEQSNKSL
SFDPFQTRGTDGVVAAGDGKSGFLGVEKKGLSLNLSVENESNKTPGGSVKNGHTKLC
SRGHWRPAEDARLKELVTQYGPQNWNLIAEHLEGRSGKSCRLRWFNQLDPRINRRAF
SEEEEERLLTAHKVYGNKWALIARLFPGRTDNAVKNHWHVIMARKHREQSSIYRRRR
PSSSASFPKGLDLTVRNNASSDQSTISCSNSTSTDLALTPSSTKKVVPPHQIFTTFSGVH
QKGEKVKVGSSGNGDFGKYGSQQRPMGVVMGVDQYANSSDSNSEISATESVGTNRT
NNSQTAIGDDKINMHFIDFLGVGAS

>GrMYB112

MMVRPPSGNDENGVKKGPWTPEEDRILIDYIQKHGHGSWRALPNLAGLNRCGKSCR
LRWTNYLRPDIKRGKFSDEEEQTIINLHAVLGNKWSAIATHLPGRTDNEIKNFWNTHM
RKKLLQMGIDPVTHRPRTDHLNVLASLPQLLAAANFNNMNNLLDVNALNRLQLDAA
TLAKLHLLHNMLQILGTPAINAMDFLSGPTFRENHQLYHLQPNTGEYQPSNLIEASQN
THPLKDLKFSSYINNDRFASSLFVANNNIPSSSIPQLPALIPASPEHRPAVVENNKINNPNE
ISNPSSTSTTFEAWGDLMDDEASDSYWRDIIDQASSQSWPIS

>GrMYB113

MRNPTKKDNVGTKTTPCCSKVGLKRGPWTPEEDELLSNYINKEGEGRWRTLPKRAGL
LRCGKSCRLRWMNYLRPSVKRGQIAPDEEDLILRLHRLLGNRWSLIAGRIPGRTDNEI
KNYWNTHLSKKLISQGIDPRTHKPLNPQQLSPSPPTLKPSPSSSSSMAKPNNPPSPLPVH

VVNANKQNDYYDGSNEDHQGMIMNNDHYQQQQDHEDDVFSSFLNSLINEDDDALV
SNLGLSQGWESTPFDQPK

>GrMYB114

MPINIITILTINTKRTYTHIHYIKKPPTFSQFQHLNYIPSLFSSPIIQFKGMKGNDKNGLKK
GPWTPEEDRILVDYIQKHGHSKWKSVPALAGLNRCGKSCRLRWTNYLRPNIKRGNFS
SEEEQLIIDLHALMGNKWSAIARHLPGRTDNEVKNLWNSRLKRKLIQMGIDPITHEPLT
DPRLHQLLAAASFSNLINNPLDIVNALMLQSDAVATLAKSLHLSHNMLQALASTPTTM
ASQDPTKACSTSNEYQFGSSSSSLPVNVPNLDTTPQPIPPMAPRPTIVDDHHETNNITNP
SSTTLQEWDDFMDGGEASEPYWRDIIDQASSQSWPIS

>GrMYB115

MGRSPCCEKAHTNKGAWTKEEDDRLIAYIRAHGEGCWRSLPKAAGLLRCGKSCRLR
WINYLRPDLKRGNFTEEEDELIIKLHSLLGNKWSLIAGRLPGRTDNEIKNYWNTHIRRK
LLNRGIDPATHRPLNEAGVHQDVSSTISFNGVKQEKDKMNNPNGFVETDEKKIPAVEE
RCPDLNLDLRISPPLYHQTQQHADPFKTGGKTLCLFCSLGVKNSRQCICSIDTAASSSG
NNTNTAYDFLGLKTGFLDYRSLEMK

>GrMYB116

MKVGACVCALLLSVHQTSKRVPVSCSCFISWINCFVLIISFHFLDKVLIFLRNPLFDKTL
VFNIRIKMGRQPCCEKVGLKKGPWTAEEDKKLISFILTNGQFCWRAVPKLAGLLRCGK
SCRLRWTNYLRPDLKRGLLSEYEEQMVIDLHAQLGNRWSKIASHLPGRTDNEIKNHW
NTHIKKKLRKMGIDPLTHKPLSTAEEQPKEQKKQVPKVDKSKPPETSLQSTITKAKEED
TSMTSSSFDPMDMTMNNENIDGFCTDEVPLIEPHEILIPGPAAPSTSSSSSSSSSSSAHS
PNFLQQFHYFPADFEWPSVNNTNKTDSNNLSLWDTDDFSGWDLLINDDDSDTKLALD
SLSSPLFQCLRMGFDQDSWTYPLL

>GrMYB117

MGRAPCCDKANVKKGPWSPEEDAKLKAYIEANGTGGNWIALPQKIGLKRCGKSCRL
RWLNYLRPNIRHGGFSEEEDNIICSLYISIGSRWSIIAAQLPGRTDNDIKNYWNTKLKKK
LLGKQRKVQQARSASCLSFKQEMKRESENYRVPGMVNQASDWPLLSPPVMPLTSTN
QDLYLRDQDSIRNILVKLGGRFSDDHPQSSTISTTTNPMNFRYPFDVYFSQDHQLYEDS
MNILSSASSISPLNSTCSQTNSTTHFSVNQVGGPNDMIQGLDAFQAELSELIYNNNGFE

GLYGTDNMVDGSSTGTSSVESSSWEDINSLAYPQIVSGFEPCQHQSIPQVSTFDESSYF
GPQ

>GrMYB118

MEQIATDNNTTTIDADQSTVSVQGRISGPTRRSTKGGWTEEEDNMLTIAVQKFNGKN
WKKIGITDVQCLHRWQKVLNPDLVKGPWTKEEDDLIFELVEKQGKKKWSEIAKFLPG
RIGKQCRERWCNHLNPDIKKTAWTVDEELILISAHGAYGNRWAEIAKLLPGRTENSIKN
HWNSSVRKKVDAMAASRINAVDHSPKAECISLGISKPLEQNLDRGRSISFFSMCKDAN
GIMKPSSLNISIKRYEDLTIENCRVHSYDPLPRNRQMHYCDSRSHCNVTFGDHERITSQ
CFLARMHAGSSFQPSSDSLHAPVSASCFRCSGNEIPDANSYISLESILRSAAKSFKNTPSI
IRKRTSQIRTQADNHNINQLPISPTKSPKLENKAHPRL

>GrMYB119

MGRAPCCSKVGLHRGPWTPREDTLLVKYIQAHGEGHWRSLPKKAGLLRCGKSCRLR
WMNYLRPDIKRGNITPDEEDLIIRLHSLLGNRWSLIAGRLPGRTDNEIKNYWNTHLSK
RLLSQGTDPNTHKKLPPDNPVVVLVPKKKRKNKSTKPTEQEKPKIHLPKPSRFTSSVCT
TKSSSQGGENGQVLVPWSEYINDDENRTGFLCYNDNHDLINSSDFECQSHHDHDHDQ
VLGAEGDSNDSLEKIYEEYLQLLKTNEDQVQLDSFAESLLI

>GrMYB120

MGRQSCCYKQKLRKGLWSPEEDEKLLRHITKYGHGCWSSIPKQAGLQRCGKSCRLR
WINYLRPDLKRGTFSQEEENLIIELHAVLGNRYIHIHIHSHIYICFFFFFLLEFLWRILGFS
RFSIRWSQIAARLPGRTDNEIKNLWNSCLKKKLKQKGIDPVTHKPLSEVENGEGNGSN
KPSLVGHHPIHQYTSPPPPPPKLSSNWFNPEFSPPMTASYGPLYYGTTTGGSANNTTWG
LVQTQTEEEEEEETKWTELLNSPLLMAAALQNQTPQSYYNIEIKSETCYLTNSSSSSSSS
NDMWWPQTQQQQLPLQHEPLQNPDICGKDMQTLTAAAFGHI

>GrMYB121

MEVYERGFFSISESGRQIEEVMDVRKGQWTEEEDSMLKAYVTLHGEGRWNSVARFSG
LRRTGKSCRLRWLNYLRPAVRRGSITLEEQLLIIQLHSIWGNRWSKIAEHLPGRTDNEIK
NYWRTKVQKQAKKLECDVNSPKFRDALRYEYTPRLIEQISRAKHESPSGQLTYGNETE
SVQVDQSLLPESSSETQVTYGSTVAETWGIDEQSNGNGWLGGGDYWMESEWCEENI
WFLQQQLYDDEDDVI

>GrMYB122

MGRAPCCEKVGLKKGRWTDEEDEILTKYIQVNGEGSWRSLPKNAGLLRCGKSCRLR
WINYLRTDLKRGNFTAKEDETIVNLHSTLGNRWSLIASHLPGRTDNEIKNYWNAHLSR
KIYRWAMKKNKSCCFNAQKDAISSSNKPMEVVPVIPSHSESEEERNMENISRPNSSSCN
TEKETGSEDIWEPCSKDIMGSELLPPNDDLSLSGYGENSIEDDLNPITSCFVDDDLFGIL
RKFSSRE

>GrMYB123

MVRAPFYDKNGMKKGAWSPEEDHKLRSYIEKFGHWNWRELPKYAGLERCGKSCRL
RWMNYLRPDVKHGNYTEEEDALIMKLHQEYGNRWSMIAARLPGRTDNEIKNHWHA
RLKTRAKRNQTSSTDSQSESAHSWEGEGESMVIDTPPNMILESSRLSPTISSSTEFSSFSP
PPIDSGYTSNLNLSGVAVEPPPYSSTEIYEDQRGGGDFWSEPFVADNVYNQDGYPSSSL
GRGGFDMPLPYDHFYDDDSPDLLLYQMMQGWV

>GrMYB124

MGRTPCCDKTGLKKGPWTPEEDLKLTNYIQIHGPGNWRTLPKNAGLERCGKSCRLRW
ANYLRPDIKRGRFSFEEEETIIQLHSILGNKWSAIAARLPGRTDNEIKNYWNTHIRKRLL
RNGIDPVTHAPRLDLLDLSSILTSTLCNQSLLNASNLLGAQPLLNPQLLRLANTLLSLK
QENPDILVQYLHKNQLSNSQQHLVPSSLQPSHLQVQSTIQNDSTCTTSTPVPCTSALNQ
IELMQNSATGISSNMTSFSCPDTQETLTATLTNTCASLPNYVHWSPNPTDPFTSENSNVQ
SVGGSQMFSFDSVRSTPITSPTPLNSPSTFINSSSSVDDEKESFSSLLKFEIPDSLDISDFM

>GrMYB125

MSTLIEEEIEVRKGPWTTEEDTLLTRYIGRHGVGPWNMLAKCAGTNTLINNLFTLFFM
FWVDLPLTSSGLKRSGKSCRLRWLNYLNPDIKRGNLTLQEQQLILQLHSLWGNRWSKI
AEHLPGRTDNEIKNYWRTRVQNKQPSSSSLKEMSSQFSVPCQLPECIVPSGSVINISQQI
EFPQHETSPNAYAHTCVDNNNLVLNGSYNIGRSGQDMEAFRLASMSAVGEGCWICNE
MTDSLWQCRELGEMGN

>GrMYB126

MQQSPCSDKVGLKKGPWTPEEDQKLLSYIQEHGGGSWRGLPAKAGLQRCGKSCRLR
WINYLRPDIKRGKFSSQEERTIIQLHALLGNRWSAIAAHLPKRTDNEIKNYWNTQLKK
RLTKIGIDPATHRPKTDTLGSTPKDAANLSHMAQWESARLEAEARLVRESKRVSNPPQ
NQFRFTSSSAPPLVSKIDVGLAHATKPQCLDVLKAWQRVVTGLFTFNTDNLQSPTSTSS
FTENRLPISSVGFIDSFLGNSNNSCCGNNWECVEKSSQVAELQERLDNSMGLHDILDFS
SDDVWFQGSYRAENMMEGYSDTLMVCDSGDHQKSLSMEPRQNFNVGTSNASSFEE
NKNYWNNILNFANASPSGSSVF

>GrMYB127

MLSKMRPPSPNRKKEVRLKRGPWTAEEDKLLTAYIQKHGYGSWGSLPHKAGLERCG
KSCRLRWINYLRPDIKRGKFSLEEEQTIIQLHAFLGNRWSAIAAHLPKRTDNEIKNHWN
THLKKRLIKMGIDPMTHKPSTSPSPKNGSNLSHMTQWESARLQAEARLVRESKQVVP
NLTTRPTRRSQLTRSSPRCLDVLKAWQGIVAGMFVFSTQDPRSLTTSTLRFPGWVEAEE
WRGQGKKGSSDADDAWFEEDSVILHSLPIANIMEGLSDAFILNSWMGVDKSTDENTV
MENGNCWDSVLNFLNSSPCGSPVLG

>GrMYB128

MGRPPCCDKIGVKKGPWTPEEDIILVSYIQEHGPGNWRSVPTNTGLLRCSKSCRLRWT
NYLRPGIKRGNFTEQEEKMIIHLQALLGNRWAAIASYLPQRTDNDIKNYWNTHLKKK
LKKVETGVDGQNQEGFSSAQSVSKGQWERRLQTDIRMAKQALSDALSLDKPNSLTNS
IEFKLSHPYLRPSQSQSSTAYASSAENISRLLQNWMKNPPKPAPAPRQTKSAETMTQNS
SCSDEGALSEATPEGFDSFFSFNSSNSDNISHESVSVAENSVFQDESKPNLGDQVPLRLI
EKWLLDDASAQAHDDDLISMSLQDSALLF

>GrMYB129

MGRQPCCDKLGVKKGPWTAEEDQKLINFILTNGQCCWRALPKLAGLRRCGKSCRLR
WTNYLRPDLKRGLLSEAEEQLVIDLHARLGNRWSKIAARLPGRTDNEIKNHWNTHIK
KKLVKMGIDPVTHEPLNKQSKTEQSPSHGHNSADNHITENDGTAANSSEDNSSTTPTE
NCSTTDDSILLDSICNDESLLTSLWLDEPPLADDSWNSTVPAAETCNDQTSLPSWEDSI
AWLLDCQDFGIHDFGFDCLNDNELNTTNTYAKQ

>GrMYB130

MVSRPSENNKPKAKHKKGLWSPEEDLKLRNYVLKHGHGCWSSVPINAGLQRNGKSC
RLRWINYLRPGLKRGTFTPQEEETILTLHHLLGNKWSQIAQNLPGRTDNEIKNYWHSY
LKKRIAKAEEMDSQARSQCTTSSSENKQFTPSPRIFSDQMPTYESFNHIDKLSVSTDRA
VPQHVIDFSKEPQRSPLPKVLFAEWLSLDQECGPAAATSYGFNHNSSSNFQDPFMDAF
LLNEGTFGGSDLHDGLSNGSANEMSSSLFDFETQISGNEFVGSLSGDDICSDFNMNNN
VMYM

>GrMYB131

MTSPSSSSSSSLKKTSTSCSEDEGDHHQQQLRRGPWTIEEDSVLVHYIARHGEGRWNF
LAKHAGLRRTGKSCRLRWLNYLKPDVKRGNLTAEEQFLILELHSKLGNRWSKIAQHL
PGRTDNEIKNYWRTRVQKQARQLNIDANSAAFKNILRYYWMPRLVQKMEESSSPCFS
TTVIPRNQPLMKTDYVDGNSCVSSLEHVDDMKMSQFGIFGSNDCNSNALAKDWYGY
VGDNGSYCHGMETINMASTSALVGEGFPTPAADCHLADDNWVNDGFVDGIWSMGEL
WELRNALH

>GrMYB132

MVDSPPSTIFILLSALLFPIFLGVEFFIELKDQQMCSRGHWRPAEDEKLRESVERYGPHN
WNAIAQKLQGRSAGKSCRLRWFNQLDPRINRSPFTEEEEERLLSAHRIHGNRWAVIAR
LFPGRTDNAVKNHWHVIMARRCRQRSSLYAKTSHTNPSKPLHHSQMGAGHGFLPLLH
KYKQGFPHNTCSPLNPNHCVTTTQDKKEAIEFYDFLQVNTDSNNKSEVTENSRRDDE
EVNQQEAMPLMEHHTKARPSFFNFL

>GrMYB133

MEFDPKLRENMSPQHQHHPMMLSENNILKPLQTTTPLSSSSSKDYYLQDFHHLDDDD
DDQIQVNVSTSSWNPVFGVNAACYDPFDAFPYGCSTNRVDFYECKPFAADHNTGNLG
HGHVMGNFQSSVGLLNLRSTTSNDVIMMGSDVGYLPSFEFPNLGDEVPCVTAENGYR
LLVDSTSSRIRTNWKGRKKTNGVKGQWTMEEDRMLIQLVEQYGVRKWSHIAQMLPG
RIGKQCRERWHNHLRPDIKKDTWSEEEDKVLIQAHREIGNKWAEIAKRLPGRTENSIK
NHWNATKRRQFSKRKCRSKYPRGSILQEYIKSLNLDSTAAAAARSHEKASDGAAVKA
ADNKSSSIKQPQGLDIGPSTDRLVPEYECDEVHEFSFDEKLLQESCSIELLLDQIPSTAA
MADEKSFQMGDMGAVKKEMDLVEMISAHPNI

>GrMYB134

MGRAPCCDKANVKKGPWSPEEDAKLKAYIEQYGTGGNWIALPQKIGLKRCGKSCRL
RWLNYLRPNIKHGGFSEEEDNIICNLYISIGSRWSIIAAQLPGRTDNDIKNYWNTKLKK
KLLGRRKQPSNIHRLSNQDPNDPHQPTGSDDNQFSQGLSNSALERLQLHMQLQTLQN
PFSFYNNPALWPKIHPLQEKMIQNMQASNGNPNLLMQPLLPPNPQPANEQSTVHQQD
YPEISNTKGLEGLDNCLDGISPSDGSVPFGNGDNLMDSTSTAAAVQPVSSFQVELDVFL
NNKSSTTGGYPQEDQMVNQLDCFRDINGSKDSLIWWPTDFDAKSASSNSWDSTSVLQ
SNGMFQDFELGYPM

>GrMYB135

MPMAPTSTKCSKKEVNRGAWTAEEDQKLAQVVEIHGPKRWKAVAAKAGINRCGKSC
RLRWMNYLRPNIKRGNISDQEEDLILRLHKLLGNRWSLIAGRLPGRTDNEIKNYWNSH
LSKKIKQNEKQTRMQEPVLENSKVSEREEPLHKASEEGSSKRDDEYSTSCFNGDSSLF
DLYNKEPLELEWMSHFFETDEIWLNLA

>GrMYB136

MPVAPPISTKCSKKEVNRGAWTDEEDQKLAQVVEIYGPKRWQAVAAKAGLNRSGKSC
RLRWLNYLRPNIKRGNISDQEEDLILRLHKLLGNRWSLIAGRLPGRTDNEIKNYWNSH
LSKKTKQNEKQTIGSRMQEPVLENCKVSESKRDENSTACFINGDDSLFDSYSEEPLNLE
WTSHFFETDELWLNLA

>GrMYB137

MGRAPCCDKANVKKGPWSTEEDSKLREYIEKYGTGGNWIALPQKAGLKRCGKSCRL
RWLNYLRPNIKHGEFTDEEDRIICSLFASIGSRWSIIAAQLPGRTDNDIKNYWNTKLKK
KLMAMSMTSQPHRKPPPFPSSHHTPSVSFSSLSSLYKDCTTYQDPTRSFTAFEPMSSVQ
SDLLSNNNTTLATNSSLIHTPEALVNYMQYYPVKENFLMFGSESSCTSSDGSSGHISFG
REMKQENMSYFQGFCATNGYEDNHNFMLNPGTNNGGKNVNQWAEKPREYNLENDL
EDFKRLISSSSNNCCNNNFIDENKTQEKIMYFYY

>GrMYB138

MMGHGHHSCCNKQKVKRGLWSPEEDEKLINYVTTYGHGCWSSVPKLAGLQRCGKS
CRLRWINYLRPDLKRGSFSPQEAALIIELHSILGNRWAQIAKHLPGRTDNEVKNFWNSS
IKKKFISHEVPALASFADVHNFKPPDEAVDFVSLNVNPSLILSDQRDQLYLSPPLLQSFA
QHHADFKSNPSGDNVDLIHHHFPLTPMLPPPLSSASSLDPSWPLPFITSQHDLDHHQDK

KHHVPVFSNEVVHDIFNEKLMNSTAAINLYDHPLMAAQTVPKLCEILEGNLYNMPLTS
VSLENIDPLGSRLSTCLPISAGSSYAHDMVVATNQMEFIDTIITSMPSASSSSSSSLSALSS
GQYITNPNLPSGGWDP

>GrMYB139

MEKNLSLKRFDVNKGAWTAEEDRKLAEVIAVHGAKRWKTIPTIAGLNRCGKSCRLR
WMNYLRPNIKRGNISDQEEDLILRLHKLLGNRWSLIAGRLPGRTDNEIKNYWNSHLSK
KVNQKEKHSGASARQGCKFAQQRLVENAKEQVREENTSTGFEESNISFDVDDFFDFS
KVDTRNFEWVNRFLEVDDGFKF

>GrMYB140

MGRSPCCDKNGLKKGPWTPEEDQKLIDYIQKHGYGNWRTLPKNAGLQRCGKSCRLR
WTNYLRPDIKRGRFSFEEEETIIQLHSILGNKWSAIAARLPGRTDNEIKNYWNTHIRKR
LLRMGIDPVTHSPRLDLLDLSSILSSSLYNQSQMNISRLLCVNSLVNPELLRLATSFMSS
QRENQNHEFIVDHNVEDNPLCSSQVQDQYQQPLMQSNHNHLPTQVQEIPACSIPFSNE
AELINMDQFPSNFSHLNDWQSNAMPSDLTEDYVPLPPNYDYYATDHHRHQTVKDPSS
SETSNFQSNNSNQSFSFASVLSTPSSSPTQLNSNSTYVNNSGIEEEPDSYCSNILKFEIRD
VLDVNDFM

>GrMYB141

MEAVNGCSSSTSSSDASSSDSYLSARGSNRAEKIKGPWSAEEDRILTRLVERYGPRNWS
LISRYIKGRSGKSCRLRWCNQLSPNVEHRPFSQAEDDTILAAHARYGNRWATIARLLPG
RTDNAVKNHWNSTLKRRAREGQHHHQQQQQEHQQQILRVTHQPMDGGDHCALGS
TEVMIEEEALTALTLAPPGSGVSSRSAVVVAERRREERVPAEFWDVMRDVIAREVREY
MSSTLSAETSAFH

>GrMYB142

MPGHNSQVTTTTSTSHTSPLPIYVKDLDRIKGPWSPGEDEALQRLVHTYGPKNWSLIS
KSIPGRSGKSCRLRWCNQLSPEVQHRPFTPEEDDTIIQAHAQFGNKWAIIARLLNGRTD
NAIKNHWNSTLKRKCSSMTQDFNDDSPQPIKRSASLGAANNVSGPCLNLGTPSGSDLS
DSSLPAASPGYRPLARTGSSKHVETASSTTNPPTILSLSLPGFDPCENSDSGPRSDPIPSPT
QVPVTTVTGHPTAGLVVGMQNGELGMEKQFFSNEFLTVIQEMIKTEVRNYMFGKFH

>GrMYB143

MGRAPCCDKANVKKGPWSPEEDTKLKAYIEQHGTGGNWIALPHKIGLKRCGKSCRL
RWLNYLRPNIKHGGFSEEEDKIICSLYISIGSRWSIIAAQLPGRTDNDIKNYWNTRLKKK
LLGKQRKEHQSRRGNSLKQDMKRSSASVGDSMVPADNINQIPYWPELPVLAAAAAPI
PHSSQEHRIDSQASMRRLLIKLGGRFSEDDHVVNDGTTLHQFPNDLSTTDQDLYEQTV
YVPSSSSSSPMDALSLSNNIGSQFVNSQFAIDGGNLPILQGQSTTFSSELQEMGYSSNPQ
RLDGMEFLYGEGMVDNRGVNPCESIGWGDTSSLVGPPCASEYGVMQQGMLQEYGFS
EMRYPGGAQ

>GrMYB144

MGRSACCDENGLKKGPWTPEEDQKLVHYIKRHGHGSWRALPRLAGLNRCGKSCRLR
WTNYLRPDIKRGKFSQDEEQTILHLHSVLGNKWSAIATHLPGRTDNEIKNFWNTHLKK
KLIQMGIDPMTHQPRTDIFASLPQLIALANLKDLLENSPHLFDDQALILQAEAAHLAKL
QYMQLLLHSAASTTTNDDSLSSPNGIADMEALNLLSSPVPAHVKENPVIPNSSQFSVG
NASSQQLHHPTLLPHLLDPQVPFSFQTSLNNENTEMGNCSSFTMLSQGDNQIDNSSSW
LLPSPTIPPTATETSLSNPGDVSSNSSNNNGAASPYWPELYFDDSIMHEIS

>GrMYB145

MEDSGAASSDDVNKTCPRGHWRPAEDEKLRQLVEQYGAQNWNSIAEKLQGRSGKSC
RLRWFNQLDPRINRRPFTEEEEERLLAAHHIHGNKWALIARLFPGRTDNAVKNHWHVI
MARKQREQSKLCGKRSFQDAFSDSKLSSTGFSTSRKLARCQEAFSSRFGFGDSRFFDQ
FQNQGKDKIFSVSSTSTTSSPSWNFASSTMVASNNPSSAQLSIRDGKNHLLRSGSSYYS
MDTSKLLDQSLYKYHSNASAYCSSFRNSSAFGIHNYRRVVPSPFGYLKLGDTFESNNG
VMTKEFLSITDNAPKSANLRVSNSQQEQEDESDKPKDVPFIDFLGVGISS

>GrMYB146

MGRSPCCEKAHTNKGAWTKEEDQRLIHYIRVHGEGSWRSLPKAAGLLRCGKSCRLR
WMNYLRPDLKRGNFTEDEDELIIKLHGLLGNKWSLIAGRLPGRTDNEIKNYWNTHIK
RKLVSRGIDPQSHRPLNGIAKATPTELDFRNAPTVPKIKPITTATPSLSFKYDESQVKAK
SDSLEGGNCTSSGMTTDEEQPSSRPNELNLELSIGFSINSANSAESKPKVAKPKAVCLC
WQLGFQRSEICSNCENTNGLFRYCTTLG

>GrMYB147

MEAVNGCSSSSSCDTSSSSSSKSSLSAMASSKSEKIKGPWSAEEDRILTRFVEKYGPRN
WSLISRYIKGRSGKSCRLRWCNQLSPSVEHRPFSQAEDETILAAHARFGNRWATIARLL
HGRTDNAVKNHWNSTLKRKAKESKQQQQRKEQISMSTYQQMDEEALSTSLTLAPPGS
GTASVAERRISVSAELWDVMRDVVAREVREYMSSTSSH

>GrMYB148

MACTRKDIDRIKGPWSPEEDEALKRLVQTYGSRNWSLVSKSIPGRSGKSCRLRWCNQ
LSPDVEHRPFTPEEDDTIARAHTRFGNKWATIARFLNGRTDNAIKNHWNSTLKRKCSS
MTDDMNDDSPQPLKKSASLNTGNGGLGLYLSSRSPSGSDLSDLSLPIASPVTITGSLVP
STQTASSATDPPTLLTLSLPGSDTSEITDLGSVSNSFPSSTLVAEPATVPALKLQMEKQFL
NAELLAVMQEMIRKEVRKYMSGSESNGLCFRTEAIRKAVVKRIGISKIE

>GrMYB149

MVVKEAKQVIKQFGKPKKEEKKDKKQKIMGRSPCCDEDGLKKGPWTPEEDQKLVQY
IKNHGHGSWRALPKLAGLNRCGKSCRLRWTNYLRPDIKRGKFSQDEEQTILHLHSILG
NKWSAIATHLPGRTDNEIKNFWNTHLKKKLIQMGIDPMTHQPRTDIFSSLPHLMVLAS
LRDLLENPLQAEAAQLAKLQYLQFLLQSSAASMASNDNYGGATTIGAEDMEGFNLLN
STQFSVGNNETSQLLHYPPQQQVPFGLQTSFNNEKKTSSNSDEMGHCSNFIVSQGDNL
IDHSVAASCPLQSSPTSPPATAGAADASVSNVPGDASSASSYNGGAASPYWSDLYFDDS
LMQDIIS

>GrMYB150

MGRSPCCEKAHTNKGAWTKEEDQRLIDYIRLHGEGCWRSLPKAAGLLRCGKSCRLR
WINYLRPDLKRGNFSEAEDELIIKLHSLLGNKWSLIAARLPGRTDNEIKNYWNTHIKR
KLISRGIDPQTHGPLNQPTNTNKSTELDFRNVPKASKSNFAPNPSRDFNFNEFQVKAKA
ESIEEGTSSSSGMTTDEEQQQQQEQDKYAGNSQELDLELSIGISSSGKNNNSTGVSTAN
SAESKRLLDKSNFQFLGQAMAAKAVCLCCQLGFGTSEICRNCQSTNGFNTYC

>GrMYB151

MSHTKHEREDGMLSKDQTESPLIDDGSCGGGGAGGVVLKKGPWTSAEDAILIDYVK
KHGEGNWNAVQKHSGLFRCGKSCRLRWANHLRPNLKKGAFTQEEEQLIIELHAKMG
NKWARMAAHMPGRTDNEIKNYWNTRIKRRQRAGLPLYPPEVCLQALQESHSTSVVN

ALDKGPNDILQNNSYEIPDVIFDSLKANQNVLPYVPELPVLSASSMLMKGLGSSQYCG
FMQPTIHRQKRLRESPAFFPGYTGAVKNECPLFMQFQEDISDKAAGSFGLSFPIEPDPAA
KNSQPFGVFPGSHTLSNGNFSASEPPLEAVKLELPSLQYPETELGNWGTLTCPPPLLES
VDAFIQSPPPTSGLESDSLSPRNSGLLDALLHEAKTLSSAKNHASDKSSNSSTPGDIAEG
SNFNICETEWEKCGEPLSPMGNSATSIFSECISASGSSLDEQPPAETVTESHVKSEPADR
VLTPEIQKEAPIRLDSSGPDTLLASNWLEQGSGYDKDQAILTDAISSLLGDDLRSEYKN
MEEGTSISSQAWGLDSCAWNNMPAVCQMSELP

>GrMYB152

MANETTTAAMQGENLRKGPWHEEEDERLISFVKLLGSRRWDYIAQASGLKRSGKSCR
LRWVNYLRPNLKHSSISAEEEMIILKLHQKWGNKWSMIARMLPGRTDNEIKNYWRTH
LRKKAVIQDQAGNFRFIQEDDNSSNSKTYNGESYNPFVDISDTQNSCYAAAPVSDFETS
PYETRLSDWISEFLSDQSEIKSQLDSTTSTTTTTTTPHSCNFYPAWFYEENDVWGYSGS
LWNMD

>GrMYB153

MGRIPCCEKDNVKRGQWTPEEDNKLSSYIAQHGTRNWRLIPKNAGLQRCGKSCRLR
WTNYLRPDLKHGQFSDAEEQTIVKLHSVVGNRWSLIAAQLPGRTDNDVKNHWNTKL
KKKLSGMGIDPVTHKPFSHLMAEIATTLAPPQVAHLAEAALGCFKDEMLHLLTKKRID
FQLQQSNPGQGNNTTVPYIKQDEKDDTVEKIKLNLSRAIQEPDMLPLNKPWESTSTRA
TSANFEGGCGVFPTSVTGYHHYGPSSFANEGGGSGSPWSQSMCTGSTCTAGEQVRSH
EKLKDENGEEFQGGKEIKNATSIFNTDCVLWDIPSDDLINPIYREAFNNKE

>GrMYB154

MNGCSSSTSSSDTSSSESSRIKGPWSAEEDRVLTRLVERYGARNWSLISRYIKGRSGKSC
RLRWCNQLSPDVEHRPFTKAEDETILAAHGVYGNRWATIAMLLPGRTDNAVKNHWN
STLKRRAREQKKEQKGSVVVDEEEVLTALTLSPPGSGGGLTVEGRREEGVTAEFWDA
MKGVIASEVREYMSSTLSSNTSKLH

>GrMYB155

MASTRKDMDRIKGPWSPEEDEALQRLVQTYGPRNWTLISKSIPGRSGKSCRLRWCNQ
LSPEVEHRPFTPEEDDTIIRAHARVGNKWATIARLLNGRTDNAIKNHWNSTLKRKCLS
MTEGFNDDSPQPLKRSASVGTGNPSSPSGSDLSDSSLPAPSPVFKTGSVVPSSQHVETA

SSATDPPTLLSLSLPGSDFSEPVSPPGFNPTQVTTQAPAPFPAPVEKQFFSAEFFAVMKE
MIRKEVRSYMAGIEQNELCLRTEAIRNAVVKRIGISKIE

>GrMYB156

MGRAPCCDKANVKKGPWSPEEDAKLKAYIEHYGTGGNWISLPQKIGLKRCGKSCRLR
WLNYLRPNIKHGGFSEEEDEIICSLYVSIGSRWSIIAAQLPGRTDNDIKNYWNTRLKKK
LLGRYLRPEPPFPVVPVPYSSQEQSIQFTNSQCSVVDGANMEQHMLQGQTSSSSLNGM
GLLYGEDIINDTSSLVCLGMFQHYAFNDRISLQ

>GrMYB157

MGRAPCCDKANVKRGPWSPDEDDTLRNYLAKHGTGGNWIALPRKAGLKRCGKSCR
LRWLNYLRPDIKHGGFTDEEDNIICSLYSSIGSRWSLIAAQLPGRTDNDIKNHWNTKLK
KKLFAAKTGVDQNSNNHESTITDSSTTSVPIEAEALVNGSTTTTTSSSYMINMKYQQN
YDYPGLVLDQIDQFTLPGLMEYSITSTANDNYSMSSSSQEVSILCNSSSFAPENNSTAWF
IDGGAEDQGILLDQLDFEGPHYLFTASGQQI

>GrMYB158

MGRSPCCSKEGLNRGAWTALEDKILTDYIKVHGEGRWRNLPKRAGLKRCGKSCRLR
WLNYLRPDIKRGNISADEEELIIKLHKLLGNRWSLIAGRLPGRTDNEIKNYWNTNLSKR
VSDRQKSPAAPSKKPEAARRGTAGNGNANGNGSGSSSTHVVRTRATRCSKVFINPHH
HTQNRDPKPSSTCSNHGDHGESKTMNELLLPIMSESENEGTTDHISSDFTFDFNMGEF
CLSDLLNSDFCDVSELNYSKGFDSSPSPDQPPLDFSDEMLKEWTAAASTHCSHQRVAS
NLQSLPPFLENGIE

>GrMYB159

MAMAPVTTKCNLKEVNRGAWTAEEDQKLAQVIDLHGPKRWKSIAAKAGLKRSGKS
CRLRWMNYLRPNIKKGNISDQEEDLILRLHKLLGNRWSLIAGRLPGRTDNQIKNYWN
SHLSKKIKLNENRNKGSEIQEPVLDNSKGNETVFVPKGSEEGTSKRDDDYNSTPCLFG
DTMSDFHSPEALNWEWMSQFFEINESWDYFAYDII

>GrMYB160

MESDIKVSTPSVGLGSSDGAQRIRPVHGRTSGPTRRSTKGQWTAEEDDILSKAVQRFK
GKNWKKIAECFKDRTDVQCLHRWQKVLNPELVKGPWSKEQEDELIIELVNKYGPKK
WSTIAQHLPGRIGKQCRERWHNHLNPSINREAWTQEEELALVRAHQIFGNRWAELTKF
LPGRTDNAIKNHWNSSVKKKLDSYIASGLLEQLQFPVLANQSQPMPSSSLRMQSTVD
DSGAKCRKESEDISECSQESNMIGCSQSASDLATAAVHTKEQFHLTEMPGVSKERNSSS
APCSEEYYPSFEDVNFSIPEIPCEVGYSSSGDYQFGEHMLITDDECCRVLFSEAVNDGCF
ASENFTQGSNIVELGGCTNTSLCQPSDIQPSETGKTPASQSGLPSRSEVLPTSCCQSFASP
SLLSVEDGTLMYGQEQSQLNCQPFGTQEQEFTMNAHDGFIFTNDDHTNDTDLQEQTY
LAKDSQKLVSVNSIGSELSAMLTCPIADDKRNLPVEQDVRGLCYEPPRFPSLDVPFFSC
DLVPSGGNMQQEYSPLGIRQLMMSSMNCISPFRLWDSPSRDGSPDAVLKSAAKTFTGT
PSILKKRHRDLLSPLSERRREKKLEIDMTSSLTKDFSRLDVMFDESVTGNTSQVSPSKR
KTNARASIEEKENVCQEFHGSLDNGGDHTEPLDDEAQKKDSNGTNSPGNIKKEAWGI
DIKDKTDAHASEKIIQQPSAVLIEHNVNDLLLFSPDRVGLKADQPLLPSSIRTPRNQCHK
SFRAISNQGLSGNACLIVSSPTLKVKNSDGHSISVTAVQCTNSPATLENLADNAGIDTAI
ENYNIFGGTPFRRSIESPSAWKSPWFINSFIPGPRIDTEITIEDMGYVMSPAERSYDAIGLI
KQLSEHTAAAYADALEVLGNETPKSIVKGRLSNNRNMDKENNGVENSSHLTSNILAER
RILDFSECETPRKETENGKSSTTAATVSFSGPSSYLLKGCR

>GrMYB161

MVRPPCCDKLNVKKGLWTEEEDAKILAYVSKHGTGNWTAVPKKAGLRRCGKSCRLR
WTNYLRPDLKRESFTPQEEELIIRLHAALGSRWSIIAQQLPGRTDNDVKNYWNTKLRK
KLSEMGIDPVTHKPFSQVLADYGNIGGLLKSRTRIGSLNRDMKNSFMIKPEPHPPQPAI
ATEGFSNINNRRVMKTMASPGIEPIQENFFPSSNINQHAATCGSLDLLSQLQAIKLVTEA
SNYGGYQIISPQFPNQYTLSLSSPSSSSSTSSTCSTTAQEKAGLAFSWRDFLLEDAFLPY
DHHPQGQDHIPEFESKDIAPQNHSGNETSAEHIDDDKNIIINNNSRVLSGMDSELLSYGI
QASSSTESSFVAAMLDQENEMFSEFANLLEDPCY

>GrMYB162

MAKSDEKPTLRKGPWSSEEDHKLIAYVTRYGIWNWTAMAKAAGLQRSGKSCRLRW
MNYLRPGIKRGNFTREEEETILDLHERLGNRWSVIASRLPGRTDNEIKNYWHTRLSKR
LRHNLVPKSGPFQIPNVETEQKSSPEIALPPAIAIKESNVETSGAIQLPLSSSNPAMKIDES
QTNCSFKAYGGLQNIFEQTFTAEGSYIVENSKAIYSVPGVSTATSQLARFQYQGNSCTD
VWRKFLTNEIIYGG

>GrMYB163

MGRALCGDKNGLKKGPWTPEEDKKLIDYIQKHGYGNWRTLPKNAGLQRCGKSCRLR
WTNYLRPDIKRGRFSFEEEETIIQLHSILGNKWSAIAARLPGRTDNEIKNYWNTHIRKIL
LRMGIDPVTHTPRLDLLGLSSLHNQYLIQSFANPELLRNVDQYQPLTQSNHCHAQVEE
TQTCSVPFYNEEQQLVMEPNVNQFPSNFNDYVMPSILTEDYVPLPPQYNYYGSDYQT
VMDPSIETSNFHPINSNQSLGLGSVVSTPSSSPAPLNSNSAYLNSSSTTEDETESYCTNIL
KFEIPDILDVNDFM

>GrMYB164

MSHTKNEREDGILSKDQTESSLIDDGNCGGGTGGGIVLKKGPWTSAEDAILIDYVKKH
GEGNWNAVQKHSGLFRCGKSCRLRWANHLRPNLKKGAFTQEEEQLIIELHAKMGNK
WARMAAHLPGRTDNEIKNYWNTRIKRRQRAGLPLYPPEVCLQALQDSHGTSAVNGG
DEGPHDILQNNSYEIPDVIFDSLKTSQNVLPYVPELPDISTSSMLMKGLGSQYCSFMPP
TIHRQKRLREATAFFPGYTGAVKNECPLFEQFQDDISDKAAQSFGLSFPIEPDPATKNSL
QFGVFPGSHNLSNGNFSASEPPLEAVKLELPSLQYPETELGNWGTFSCPPPLLESVDAFI
QSPPPTSIAESDSLSPRNSGLLDALLHEAKTLSSAKNHASEKSSYSATPGDIAESSTFNIC
ETEWENCGEPLSPMGHSATSLLSECISASGSTLDEQPPAETFTDCFSESHVKSEPADYVF
TPEIQNEAPIRLDSCHPDTLLASNWLEQDSGYDKDQTIMTDSIAALLGDDLSSEYKNM
AAGTSISSQAWGLGSCAWNNMPAVCQMSELP

>GrMYB165

MAKSVENRALKKGAWSPEEDKKLIAYIKRYGIWNWAEMAKPAGLQRSGKSCRLRWV
NYLRPGIKHGNFTKEEEETIIDLHEKLGNRWSVIASKLPGRTDNEIKNHWHAHLSKRL
KYDLNSLPDMSDAEIDQYSSFETDPPPTNVPNALISESSAATSTNSLPSSSSNPKQ

>GrMYB166

MGRTPCCDKNGLKKGPWTPEEDQKLIDYIQSHGYGNWRTLPKNAGLQRCGKSCRLR
WTNYLRPDIKRGRFSFEEEEAIIQLHSVLGNKWSAIAARLPGRTDNEIKNYWNTHIRK
RLLRMGIDPVTHSPRLDLLDLSSILGCCSFYNQSQMNMSTRLLGGGGGVQPLVNPEIL
RLATSIMSSPQRENQNPDILFHENQYQQSPLMQNTNVAEPVMNNNPNVIIDQFPFNGCF
TDWQNNANAVLPYLTEDNYVPVPSNCYGGGNGEPSFRTPSSSSPTPLNSNNSTYINSSS
TEDESYSSDILKFEIPDFLDVNEFM

>GrMYB167

MEEVRGRRVAFGYGNLLCNNNCKPSLCKSSPPLSAIDRFLWGQTQSLSSHSQAAHQPP
YSVQNNVKINKGTVLGSTAAALLRGFSFPSDAIGGYLPRQTNFEESFLDGLFVDGEILA
LTEDKNPNKEMKASMKGDFPKGVVKRNKKVASAALIKGQWTDDEDRKLIRLVKQYG
VRKWAQIAESLVGRAGKQCRERWHNHLRPDIKKDSWSEEEERILIEAHAKVGNRWAE
IAKFIPGRTENAIKNHWNATKRRQNSRKKNKQNDNQNGKPQSSILQDYIRSQNLNTPT
NSSTTSATPSSSTFSEDLSTQFKYFLPEPSESDDSHPLVVQTYDDELMFMQNFFANNNN
TIHPSLDYSQTGNPTEVFKPVHFVDHNLSKGSSTIDCPTFADTRFGFSSIQEPKNEPRTT
YLFSDLYLSRLLNGATTSSFPNGDGYYNGDVNTNLLSGQASSDGRKEMDLIEMVSSSQ
FYT

>GrMYB168

MGHHSCCNKQKVKRGLWSPEEDEKLINYITTYGHGCWSSVPKLAGLQRCGKSCRLR
WINYLRPDLKRGSFSPQEAALIIELHSILGNRWAQIAKHLPGRTDNEVKNFWNSSIKKK
LISHDHVPALASFADVHSSNHTEEAGFISLNANPNLILTAQQDQLYLSPTAPVLQSFCHH
ADLVHHHFPLTPMLPPPPPPSNTASFDPAWTLPFGPQHDQDDDQHHQVQVFNNEAAQ
NFVSDKLMNPPFDNPLMGPPTVPKLCEILEGNMVCNIPQTSSVSLENNIDPLVSRLSSC
FPPIPAGSNYAHDMQVGASQMEYIDTIITSIPSSSSSSSLSALSSGQYLTNPNLPSSSWDP

>GrMYB169

MGRSPCCDKVGLKKGPWTPEEDQKLLAYIEEHGHGSWRALPVKAGLQRCGKSCRLR
WTNYLRPDIKRGKFSMQEEQTIIQLHALLGNRWSAIATHLPKRTDNEIKNYWNTHLKK
RLAKLGIDPITHKPKSDALLSTDAQSKSAANLSHMAQWESARLEAEARLVRESKLRSH
SHSFQHRLTRPPTAAFASSAGRLVNKTAWNSTAGWSKSSEVNNGVVNNGFGDLESPKS
TLTSSENGVGLSSMGMPDFVGTASASSEIKQEGEQEWKGFGSSTNLAMENGFNDIGN
AMEDGFINLLLNDSTDPSLSDSGKESDGNSGDGTASDDHYEDNKNYWNSILDLVNSSP
SGSPMF

>GrMYB170

MVRAPCCDKMGLKKGPWTHEEDQILISYIQKHGHENWRALPKQAGLLRCGKSCRLR
WINYLRPDIKRGNFSLEEEETIIQLHEMLGNRWSAIAAKLPGRTDNEIKNVWHTHLKK
RLKQYQTKPDNTKKNLKSKTKIKSEPSTTSHSESDEVPSSSSEVVSSIIDGSDHREDNN
MDTWECLVEIDESFWSDALSSDESQVPSLPTDNIMEPNYTFGENLDDSMEFWYDLFIK
AGGSEQGFITQF

>GrMYB171

MGRSPCCEKVGLKKGRWTAEEDEILANYIKANGEGSWRSLPKNAGLLRCGKSCRLR
WINYLRADLKRGNITADEEETIVKFHSALGNRWSLIAAQLPGRTDNEIKNYWNSHLSR
KIYSFSKTIKETKPTDLDAIKRAEDHQRKRRCGRTSRSAMKRQKLALMSLGISKTVTP
NAQESSHGETLEMHGSCTHSNASGQPQGNYDESGSNGGIALSTNSGEESSGDGIENEV
LLGPYEWLDNEIKRLSCILQRSQGADPIGNHGGVADSINGVIKDTQNDARERESYGIGS
SSSNTTEITDHHGDEWQMCNSSVDFIGDFQWCDDHQWELSWDDLEKVFCWSWDDA
NGDDEAGKNT

>GrMYB172

MDVRGRDCVPKAQSSEEDQMELRRGPWTVEEDFKLIDYIATHGEGRWNSLARCAGL
KRTGKSCRLRWLNYLRPDVRRGNITLEEQLLILELHSRWGNRWSKIAQHLPGRTDNEI
KNYWRTRVQKHAKQLKCDVNSKQFKDTMRYLWMPRLVERIQAANAASSTSTTAVNT
AVVGTEPMVFPDDHHLGGGAQQVTSSSNNNYTLENSSTTAASSDSFGTQVSPVSDFTD
YYSNISINHNPNPNCFEAGNYYNNGLDFQCLEQNNPWLDTVDGSDSIFDAEDLYFLQQ
QFNFNM

>GrMYB173

MEDEKESNRQDSPAAGGSGSGSGGDELELIVAESELGGGGGSGVGGGGGSSSNNNNT
RVKGPWSPEEDAVLSRLVAKFGARNWSLIARGIPGRSGKSCRLRWCNQLDPCLKRKPF
TDEEDRIIISAHAIHGNKWASIAKLLPGRTDNAIKNHWNSTLRRRCMELGRFKPGPADT
MEDGSFERTKASSEETLSVGDVNHFKHLEGRDMVMDDRPNLQEDKPPIQEDQFAIEP
KNHPAVCRPVARVSAFSRYNTPSSSKTESGMTSRIPVQGPLAQSSRPDWGVGKILEDLR
CEPIIPLRCGFGCCSTPCGGHSRTSLLGPEFVDYEEPHVFSSHELISIATDLNNIAWIKSGL
ENSCVRIPSNATSQRMSQGSDIHTKSDPMCFTDGQSKLTGMSTEVLPTQTCTMRSEVE
GLS

>GrMYB174

YTFCFWCKLLKYGAKLSVYIIEVVVEVILSLKQATQSLDDPSLRPHSSSNLYRLSNHTY
PILETIMGVKKERPVSSKRSQINKGAWTSEEDTKLAEVIAVHGAKSWNTIASKAGLKR
CGKSCRLRWMNYLRPNIKRGNISEQEEDLILRLHKLLGNRWSLIAGRLPGRTDNEIKN

YWNSHLSKKIKQKEKQGCKDEKRSVENGKEELRQENTCAAGGEDSNISFDVDEFFDF
SDEKFEWMNR

>GrMYB175

MSHTKNEREDRMLSKNQKELPLIDDGNCGDADGGALLKKGPWTSAEDAILIDYVKK
HGEGNWNAVQKHSGLFRCGKSCRLRWANHLRPNLKKGAFTREEEHLIIELHAKMGN
KWARMAANLPGRTDNEIKNYWNTRIKRRQRAGLPLYPPEVCLQALKESHRISAVSGG
DKGTHDILQNNTYEIPDVIFDSLKANQNVLPYVPELPDISASSMMMKVLGSSQYSSFM
PPAIHRQKRLRESAAFSPGCTAAVKNDCPLFEQFEDNMFNKAAKPFGQSFPFEPDPLTK
KSQSFSAFQGSHALSHGNFSASEPTLEAVKLELPSLQYPETELENWGTFSCSSSLLESV
DAFIQSPQTTSAVVLDSHSPRNSGLLDALLHEAKTLSSAKNHASDKTSNSSTPCDIAER
SNFNICETEWENCGETLSPMCHSATSFFSESISASGSGSSLDEQPPAETFTEPHTKSESSD
HVLTPEVEKEAPIWSDGRCPDILLASNWLEQGSGYDKDETIMIDAIATLLSDDLSSEYT
SMLAGTSVSSQAWEPSCCAWNNMPAVCQMSELP

>GrMYB176

MGRSPCCDKVGLKKGPWTPEEDQKLLAYIEEHGRGSWRSLPAKAGLQRCGKSCRLR
WTNYLRPDIKRGKFSLQEEQTIIQLHALLGNRWSAIATHLPKRTDNEIKNYWNTHLKK
RLAKMGIDPITHKPKNDALLSTTDGQSKKAANLSHMAQWESARLEAEARLVRESKIR
SHSLQHHHHFNPPAFTLESPTSTLSVSENAPPIITGLGVSPMPMIEFVGTTSGSSETAGIV
KEEGEQEWKELGSSSNLADYKEGMGNSLSSFTSSLQDMTISIEGGWTPESLRPNNINV
NNVGNIMEEGFTNLLLNDSFNRSLSDSGKESDENSGGSGDGSDYYQDNKNYWNSILN
LVNSSPSDSPMF

>GrMYB177

MGRPPCCDKVGVKKGPWTPEEDIVLVSYIQEHGPGNWRAVPTNTGLLRCSKSCRLRW
TNYLRPGIRRGNFTEHEEKMIIHLQALLGNRWAAIASYLPQRTDNDIKNYWNTHLKKK
LKKLHGSEVYCRDGFTSSAASDQISRGQWERKLQTDINMAKQALSDALSPEKSSDLTE
LKPCHGNTYAKPEGYASSTENIAKLLKGWMRKNPLKPASTNSGVTQQSYDNMVATGV
TTDSANSSEGNDQRCSKPMCEGFESLFVFGSFDSSNSDDFSQSISTEASLSLQDETKPD
LSPQLSLLEKWLFDDAANQGKYYQLSDITLDENPSFFLEGGI

>GrMYB178

MSMKKEGEILYKKGLWTMEEDKLLIDYVKVHGKGQWNKIANRTGLKRSGKSCRLR
WMNYLSPNVKKGDFSEEEEDLIIRLHKLLGNRWSLIAKRVPGRTDNQVKNYWNSHLR
KKLGIIDQNETRIDFCQSSKQVKVCHVDEAATDPSPGHGTTTETTGITVDQSNQQEAID
HRVLNNTTQESMTSESYINTFWIPDHDYELSTLAMIDHFHEYSSFHLS

>GrMYB179

MINGGDRVKGSWSPQEDANLIKLVEQHGPRNWSMISSSIPGRSGKSCRLRWCNQLSPA
VQHRPFTAAEDAVIIQAHAVHGNKWATIARLLPGRTDNAIKNHWNSTLRRKRETELSS
GSSESNSGDKRPSQDASESESGNKKQCLGLVLHEQEQEHENVGMLEPKTLLTLSPPGE
NMEEEAVVVVVKSEESGEEKQRAVVDETACLLSIMQRMIKEEVRSYIDKIIADQNPLK

>GrMYB180

MGRTPCRDKNGLKKGPWTPEEDLKLINYIQIHGAGHWRNLPKNAGLQRCGKSCRLR
WTNYLRPDIKRGRFSFEEEETIIQLHSVLGNKWSAIACRLPGRTDNEIKNYWNTHIRKR
LARNGIDPVTHAQRLDLVDLSSSIISSLLGVQALLNPQLLSLANTLLSLKQENPELLLQY
LQQNQLLQTPTLEPRSQVLQSPMTDCSNQNSQKSYVLVSQPNYEHSNPNPTVPSLVSD
NSHFHSMDGSQNFGLDSVRFTPISSPTPLHSSSTFINGSYTTHYEIERFNSLLKYEIPESL
NINDLL

>GrMYB181

MVRTPYCDKSGLRKGTWTPEEDRKLTAYVTRYGCWNWRQLPKYAGLARCGKSCRLR
WLNYLRPNIKRGNYSKEEEETIIRLHESLGNRWSAIAAQLPGRTDNEIKNHWHTHLKK
RFMDKQNNREKAVEKKEKKLNNHHHLVIDVPTSPPLILESSLSASPQPTSSDQYSTITK
DNTVLSSKDSVTYDHNNNNKASLEGYEANSGNFWNEPFFWDSCNINTSVTLDPASYE
LPEFPFLDGEISCHFDSYDPLDGFYF

>GrMYB182

MDRDVDRVKGPWSPEEDELLRKLVHRHGARNWSLVSRSIPGRSGKSCRLRWCNQLSP
EVEHRPFTSEEDGVIVKAHAKYGNKWATIARLLNGRTDNAVKNHWNSTLKRKLSEAE
DGDSVQESEKRSVKSPRRESPSGSEVSDLGLSVAKSNQHSADVVSTELTLGRSWNESF
EFNNSSSSEKKNEQPLEEKQVAGKNMTTVALAPAATAALTPAAMQEMIRKEVRDYMA
EFDCNSENLRDLWGRED

>GrMYB183

MGRSPCCEKVGLKKGPWTPEEDQKLLAYIEQHGHGSWRALPLKAGLQRCGKSCRLR
WINYLRPDIKRGKFSLQEEQTIIQLHALLGNRWSAIATHLPKRTDNEIKNYWNTHLKK
RLTKMGIDPVTHKPKTNALGSTTGNPKDAANLSHMAQWESARLEAEARLVRESKLVP
SNPPQSNHFTAVAPSPTPATRPQCLDVLKAWQGVVCGLFTLNMDNNNLQSPTSTLNFM
ENTTTLPMSSSSVNGMFNENFGWNSSFNPCESGDILKVEYGSDQIPELKERLDHPMEL
HEMDCSSEGTWFQELFGFNGL

>GrMYB184

MVRAPFYDKNGMKKGEWSAEEDHKLRSYIQRYGHWNWRELPKYAGLKRCGKSCRL
RWMNYLRPELKRGNFTEEEDALIIKLHDEMGNRWSTIAKSFPGRTDNEIKNQWHAHL
KKRTKRDEKEKSDCWQSEATRNENICEGEGEGEDESNSILVDTLDNMILESSPLSPATC
TRIEQSSFSSGRGPMFSFNVVGGLEDNCLPCLGTYKNEGSGDFWSEPFVADNTSSLEK
GGFEMLLEYEDMYHDDSAYLRYEFTQGWI

>GrMYB185

MVRAPFYDKNGMKKGEWSAEEDHKLRSYIQRYGHWNWRELPKYAGLKRCGKSCRL
RWMNYLRPELKRGNFTEEEDALIIKLHDEMGNRWSTIAKSFPGRTDNEIKNQWHAHL
KKRTKRDEKEKSDCWQSEATRYENICEGEGEGEDESNSILVDTPDNMILESSPLSPATST
RTEQSSFSSGSGSMFSFNVVGGLEDNCLPCSGTYKAESSGDFWSQPFVADNTSSLEKG
GFEMLLEYEDMYHDDSAYLLYELTQGWI

>GrMYB186

MKKGEWSAEEDDKLRTYVQKYGHWNWHQLPKFAGLKRCGKSCRWRWMNYLLPGL
KRGNFTEEEDALIIKLHEQFGNRWSTIAKSLRGRTDSEIKNHWHSQLKKSTKGDEEEK
CSSWQSEATQNENICEGEAESNSIDNMTLGSSPPSSPSPSSSSGSMSSLNAVGRLEDTRL
PYLEIYETESSGDFWSQPFVTDNTSSLEKGGFELPLPYDD

>GrMYB187

MNRPGCSSTTIPSSSSSSLGSSMGMVYPDMGSLSLGQNYGILGSSVSSTQDSYGCKVPE
MENERASWGFHFMGNCKTRSFEENHSSDVVEGQSSDCSDGFGDDSRTINLNAILNEE
NPNDNTVSGKETDSGQSKLCARGHWRPAEDTKLKELVALYGPQNWNLIAEKLEGRSG

KSCRLRWFNQLDPRINRRAFTEEEEERLMQAHRLYGNKWAMIARLFPGRTDNAVKNH
WHVIMARKYREQSTAYRRRKLSQSVYRKMEETPTFVCRDAATKAEPPPYCLNIPNRR
LGTISHYQFGTFNGANAGVNGGSNVSPDSSSEVPRKGFIAQQPPFDFIPGVKSNDMMSI
IRQTRYWDRPIDEPQISGFYPHQHHHPSYIMAMQQSEFLSSQGLTDSTAPTAQISGSEPS
SSVPGTKAATSSHYETVPPPFIDFLGVGAI

>GrMYB188

MGWRLPFNGHKEVGLKRGPWTAEEDQILMAYIQQHGHGNWRALPEKAGLKRCGKS
CRLRWINYLRPDIKRGKFSLQEEHTIIQLHALLGNRWSAMAAHLPKRTDNEIKNYWNT
HLKKRLIKLGVDPMTHKPRTDASSFPSGSNLTHMAQWESARLEAEARLVRDSKQVIP
NPIHPKNHLTLNHIQLRPRCLDVLKAWQGVVAGMFAFPTQDLGSPTSTLRFPAIGLNAT
SYTDGEMGFDDSLKCIENSNQMKEIEETITDGCIDEWFEDSFRVGNYENVPMAVGSSN
CCDSVFDLVNSSPYGLSMLYDHNVMK

>GrMYB189

MGRSPCCDKVGLKKGPWTPEEDQKLLAYIEQHGHGSWRALPAKAGLQRCGKSCRLR
WINYLRPDIKRGKFSLQEEQTIIQLHALLGNRWSGIAAHLPKRTDNEIKNYWNTHLKK
RLNKMGIDPVTHKPKTNALGSATGNPKDAATLSHMAQWESARLEAEARLVRESKLVR
FSSSSSSSSSAPQHTSNPVMTPPATRPQCLDVLKAWQGLVTGLFTFNNTTDNLQSPTST
LNFVENTNTLANGLINDNSMELHEMGAWFRQDSSYRAVENMNMEDYSDMMVWESG
DHQQCSSMAAPAENLNETSYGNSSSSSSSSSLEETRNYWNNILNLVS

>GrMYB190

MADCREKMGLKKGPWTPDEDQKLLAYIEEHGLGNWRTFPEKAGLQRCGKSCRLRWI
NYLRPDLKRGKFSLQEEQTIIQLHAFLGNRWSTIAAHLPNRTDNEIKNYWNTHVKKRF
TKMGIDPTTHKPKSNHVVSPTGRTTLNHMAQWESARLEAEARLVKDSKNLPSSSSRPS
PYQKSCNKGSKSQCLDVVKAWQSVVAGMFATSTNNSNRIIFGPDQSSGNYELDSIIPIG
GNVEDELMVGNDRSKCQVPELNERFDNYMSLHDTTHLWAAPIAENDVVEGFPDFLV
HDFDYQIDNEESITV

>GrMYB191

MTELSLFHHLSMEDSSDDVKTTSCPRGHWRPAEDEKLRQLVQQYGAQNWNSIAEKL
QGRSGKSCRLRWFNQLDPRINRRPFTEEEEERLLAAHRIHGNKWALIARLFPGRTDNA

VKNHWHVIMARKQREQSKLCGKRSFQDGLIDSKLCFAPRKSRCQEGFSSRFGFGDSRI
FEFQNPSKDKIFSVTSSSSTSSPSWTFASSTSMASNNTSLVELSGRDHGLAMGGSNLLD
QSLYKCHSNASAYCSSFRNFSAFGLPNYRRVVSSPFRYLGLEINNDVVKEESSSFTDNA
SKLTDIRVASNSHQEQDDDSIKTKDVPFIDFLGVGISS

>GrMYB192

MGRSPCCDKVGIKKGPWTPEEDILLVSYVQEHGPGNWRLVPTNTGLQRCSKSCRLRW
TNYLRPGIKRGNFTPHEEGMIIHLQALLGNKWAAIASYLPQRTDNDVKNYWNTHLKK
KLKKFQPAMELPQMAQACLNKTLATGSSSTIATYASSTENISRLLQGWMRASPNICSN
QSAIILQTQSKDESGGRDLMSNEHFESILRFENMNNVAWEKSACGSTTSKGGFQDSGN
DDQFSVEITQEMKQKTGNNSKFYPSFSSLEKWLLDDSWAGKVEEMNQWMELSPIF

>GrMYB193

MKKPILLGTSTATSRTPCCSKVGIKKGPWTAEEDEVLANYIKIEGGGRWRTLPKRAGLL
RCGKSCRLRWMNYLRPTVKRGPIAPDEEDLILRLHRLLGNRWALIAGRIPGRTDNEIK
NYWNTHLSKNLISQGIDPRTHKPLSPVSHGSSQANQDDDDDVPESNPNPPSSFKSSEL
MGEKTNSMETTNLESHQIYQHQQEVGDDCMGQPSSSGNGGDLVENGNEDHMFSLFL
DSLITDNQQQQQQSNNGELWEAEIMSPMVEFGNPQNYFNHHQQHP

>GrMYB194

MGGVAWTEEEDYLLKKCIERYGEGKWHRIPVLAGLKRCRKSCRLRWLNYLRPNIKRG
SFAAEEVNLIINLHSLLGNRWSLIAGRLPGRTANDVKNYWNCHLSKKLNSAPQSEDDQ
TAAATTTASMKPRRPAHVSPNTQQETSVWAPFHDVQVNQQGQEVAAEEPVTLVGDLE
FDEGGSSKWWDDFIFDMDLWTASL

>GrMYB195

MGRSPCCEKAHTNKGAWTKEEDQRLINYIRVHGEGCWRSLPKAAGLLRCGKSCRLR
WINYLRPDLKRGNFTEEEDELIIKLHSLLGNKWSLIAGRLPGRTDNEIKNYWNTHIKR
KLISRGIDPQTHRPLNQTANTNTVTAPTELDFRNMPTSVSKSSSIKNPSLDFNYNEFQFK
SNTDSLEEPNCTASSGMTTDEEQQEQLHKQQQYDPSNGQDLNLELSIGIVSADSSRVSS
ANSAESKPKVDNNNFQFLEQAMVAKAVCLCWQLGFGTSEICRNCQNSNSNGFYSYCR
PLDS

>GrMYB196

MVRQPYLKKGTWSRDEDHKLIAYIVRYGIWNWNEMPKHAGLQRSGKSSRLRWMNY
LRPNIRRGNFTREEEETIVRLQKILGNRWSAIAARLPQRTDNDIKNYWNTRLKKRVISE
NKNSTAATTSTKETNSSMMENSSDADSSSMLTNILLDSPVSTIDDLLEPPAYSCFSPSGS
DLVAVDDKYSMSTDNFLVSCWETQSYLEQPQMIEGLDWEAVLPNSELWHSHHQQCY
DPLDDFWINPLI

>GrMYB197

MVRQPYLKKGSWSRDEDQKLIAYITRYGIWNWNEMPRFAGLQRSGKSCRLRWVNYL
RPNIRRGNFSREEEETIIHLQKTLGNRWSAIAARLPQRTDNDIKNYWNSRLKKRVIVEN
NNSASSPTETKSKTKSSVEENSSDADSSMMLGIFLDSDTRGMHDFAEIAADTTFFPSGS
DPSVAVDDHYSMAMDNYLVSYWEILSFLEQPQMIEGLDCEAISQNSQQWHSHHQQY
YDPLHDFWVNPLI

>GrMYB198

MVRQPYLKKGTWSHDEDQKLIAYIRKYGIWNWNEMPKFAGLQRSGKSCRLRWMNY
LRPNIRRGNFSREEEETIIHLQKTLGNRWSAIAARLPQRTDNDIKNYWNTRLKKRVMG
EKKNPSSATTETKSSMEENSSDADSSMMVDILLDYQIPTMDDFPELAADTTISLSDCNL
SVAVDDHYNMAMDNNLVSSENYWEIENLWGQSLTMEGLDCEVMSPNSQLWLHEPIY
ACDSYYDPVVELWVNPFI

>GrMYB199

MMGNNNNNHQVSMQKEEGVMLKKGPWTAAEDAVLAEYVRTHGEGNWNAVQRNT
GLARCGKSCRLRWANHLRPNLKKGAFSPEEERIIVELHAIMGNKWARMAARLPGRTD
NEIKNYWNTRVKRRQRQGLPLYPPEIQSLYSQTQPVLTPPSTSSFSFQHSTTMLTSSSPSP
PPRLLSYDPHSTATPPPVQSPSPASTPPPLPSPSHVSPLQSPHKPSFSSIPLADSFTSNTPSS
SLDFYFPRPLPSLEQPLRNKRPRHDENNTNNGGSSFMLPFSSLMKTDPFNPHTVTNNSL
NPQHYSNSYCLDQTTFDMASSSRFLQPHFDPGQFISTPRFGYNQLKTELPSNQIFTQDG
NSQVRFDPKGNNNYSSHNQIQNSNHHSSGLNISGNGLVEDMLQEVQALNEKNEIMAS
QSCLVRPSSSSEGLASGLEAKEESKEQIMNTTKHVDYSHVNPSSMAMAIPEWYINNGE
SSIGQSSTVTYDDLALEFELHDQIASLLPIDTAPDHHGRSSSSYTWVHSFPGIC

>GrMYB200

MEQSNKCLSFEPLENRETMRTSGCDGKNGVWGVEEKGLTLNLDGEEDESNKNSSAVS
GCKSGNTKLCSRGHWIPAEDAKLMELVTYYGPQNWNLIAEHLEGRSGKSCRLRWFN
QLDPRINKRNFTEEEEEMLLVAHRLHGNKWARIARLFDGRTDNALKNHWHVITARKH
REKSGVYKRKKPSSYASRALPKGLGLTIVNSACSASDQSTISSNIDQSAKVSSPVHQMD
VSRRCSLFENVATRGIGYVVYQQGLMEAVMSVNRSATSLDSNSEVSAAESVGTNWTS
HFISGESCGNGNEKIPFIDFLGVGRAS

>GrMYB201

MAMALFESSITENQRGRRGGREKEAKEAMGRPPCCDKVGIKKGPWTPEEDIILVSYIQ
EHGPGNWRSVPTNTGLMRCSKSCRLRWTNYLRPGIKRGNFTPHEEGMIIHLQALLGN
KWAAIASYLPQRTDNDIKNYWNTHLKKKLRKFQSAMEPPSMAGDEKSLDFAESETRA
GSSNLKLNQTSSSYASSTENISRLLQGWMRSSPKINNNNNSDSIGSCSAAANCDLISHG
DDQLESILSFENMKDVALMEKSTSKATFQDSGEKIINPERKQKADNSNNNIDDNNNNN
SNPPLSFLEKWLLDESSSAAQVEEIDQMMELPSIF

>GrMYB202

MGRAPCCDKANVKKGPWSPEEDAKLKAYMEKHGTRGGNWIALPHKIGLKRCGKSC
RLRWLNYLRPNIKHGGFSEEEDNIICNLYLSIGSRWSIIAAQLPGRTDNDIKNYWNTKL
KKKLLGRHKQSDINTNLSDHSNNNNNQFPQGLTNSAMERLQLHLQLQALQNPIFSFY
NNPALWPNILPLQQKVTIQGASNGSTPNIVMEPAAAATTPLTGDTLVDSKPVAMGPLVS
SFRQGEVVDEFVGVQEGGQMAEVDGFDGLKDSMVWWSNGFDAKVGSSNCWGSAS
SVVQFNEMFKDYELGFNM

>GrMYB203

MMRKPNNGSTITTTNNKLRKGLWSPEEDDKLINYMLTNGQGCWSDVARNAGLQRCG
KSCRLRWINYLRPDLKRGAISPEEEELIVQLHSILGNRWSQIAARLPGRTDNEIKNFWN
STIKKRLKNSSPNTIGSSTSNFNKDSNPVGFITMEQQGVLLPTYIDLSSTSSNSSLQSTVT
NPGTAFGATVGYFATNVNCMYGENEMLCGEELYMPPLETVRENLKIENTFESDITTTT
TTNNNNNVDCSMKSENVMTGAAVGNFWLGEEIKVGDWDLEDLMKDVSSFPFLDFQS

>GrMYB204

MEMVKDESCRKGPWTEKEDMVLVNFVHLFGDRRWDFIAKVSGLNRTGKSCRLRWV
NYLHPGLKRGKMTPQEEKLVLELHAKWGNRWSRIARRLPGRTDNEIKNFWRTHMRK
MALEKKRSTSPSSSSSSTVTRVDSLPSSGTGKVSFYDTGGPKMAVFDDIKGYSMDEIW
KDIDMSEGNTTKLPLSENYSEQGCCNHFSCPSMASSPPWDFSWDYSLWKMDDDDDD
DDEEERKVFIATTNNQLFNLFVHKP

>GrMYB205

MGRAPCCDKANVKKGPWSPEEDSKLKDYIEKYGTGGNWIALPQKAGLKRCGKSCRL
RWLNYLRPNIKHGEFTDDEDRIICTLFASIGSRWSIIAAQLPGRTDNDIKNYWNTKLKK
KLMAMNGIPSQPQRKPPPPPPPFSHQPQPVSSLYEDSTAYYYTPFEPIMSSAQSDLLNNT
NLTNNSSLIQTPESLFSHMQYHPMRDNFLMFGGEPSCSSSDGSYGKEMKQEDHMGFQ
GFGASLDYGTNNGVNQWIEKQSGYSGECPLDYNPEDVKRLISSNNNSFFIDENDTQD
KAMYCYYY

Figure S2. Nucleotide sequences of 46 stress-responsive GaMYBs.

>GaMYB081 Cotton_A_01754 locus=CA_chr11:78950087:78956471:+

ATGCGGAAAGGTCAGTCTGATTTGTCATTAGTTGAAGAAGTTGGTAGGAGAGGCAACATGAGAGAAAGCT
GTCATTTGAAGAAAGGGCCTTGGACTTCAGCAGAAGACGCGATTCTGGTGGACTATGTAAAGAAGCATGG
TGAGGGAAATTGGAATGCTGTCCAGAAACACTCTGGACTTTCACGATGTGGGAAAAGCTGTCGCCTGCGT
TGGGCAAACCACCTAAGGCCGGATTTGAAGAAGGGCATGTTTACTCCAGAGGAAGAGCGTCGTATAGTTG
AGCTACATGCTAAGTTGGGAAACAAATGGGCACGAATGGCTGCAGAGTTGCCTGGACGAACTGATAATGA
GATAAAGAACTTCTGGAATACAAGAATTAAGAGACTGCAACGTGCTGGCTTGCCAATTTACCCTCCTGAT
GTGTGCTTGCAAGTAAATTTAAGTCAAGAAGAGAGTCATAACGTGGTTTCAACGCCAAATCAGGACTCCT
TTGGTTCCAATCTCGTGCAGTCAGATGCTTTTGAGATTCCACATGTGGAATTTGAGAATTTAGAGCTCAA
CCAACAATTTTTATCTTATTCACCCGAACTTCTCAACATTCCTCCAAAAACTATTGGTTTGTCCAACGGTT
ATGGCCATGTGTTCCCAATGGCATTCCCTCCGAAGCATCTTCAGGAAAGTGTCAGCAACTATATGTCATTG
CCCAACCAAATGACTGATTTTGCTTGCAAGGAAAATTTTGATGAGCCCTACAAGTCATCTTCTCCATATGA
TTCTGATTTCAGTACCAATGACCAATCATCATTTGGTATACCTCCTGGCAGTGATGCCCTTCTAAATAGTA
ATTTCTCTCCTTCTGAGCCCTTGTCCGGGCCTATGAACTTGGAGCTCCCTTCATACCAATATTCAGATAAT
CAACAAGATAGCTGGGTCAATTCTTCAGTCCCGCTACCTTTGGTTGAGTCTGTTGATACTCTGATCCAGTC
CCCTCCAATGAAGCGGGCTAAGTCAGAATGTTTTGCACTACAAAACAGTGGTCTGCTTGAAGCTGTACTT
TACGAGTCAAAGAAGCTTAATAGCTCAAAGGATGATTCTCGTCAGCAGTCTTCTAATTTGATGCTTGACG
ATGTTGCTGATTTTCACCTGAAGGATTGTGAATTAGAATTCGAAGCACACTGCGACCCGAATTCTCCTTC

AGCTCTTTCTGCTGCATCCGTTCTTAGTGAGCACACTCCTGTTAGTGGAAGCTCATTGGATGATGAATCAC
AGTTTGTTGAGAGCATTCTGGGAGGCAACGGCAAGAATGAAACTCCGAATCATATGGCTATTTCTGGACT
AGATGATTTTCTGGGACGTGGTCAGTTTGGTCATGACAATGGGTGCGTGAACGATAAACCCATTGTAACA
GATGTTATAGCGGCTCTTCTCGGTGAAGATACTTGCTGTGGCAACTAG

>GaMYB089 Cotton_A_09629 locus=CA_chr11:32749955:32751942:+

ATGGATGCTAAAGCAAAGGGAAACGTACGAAGGGAAGAGGAGGATGATCAAATAGAGCTGAGACGAGGA
CCATGGACTGTTGAAGAAGATTTAAAGCTCGTTAATTACATTGCTTCTCATGGTGAAGGTCGATGGAATT
CACTTGCTCTTTTCGCAGGTCTCAAAAGGACAGGAAAAAGTTGCAGATTGAGATGGTTAAATTATCTTCG
ACCCGATGTTCGACGTGGGAACATCACCCTTGAAGAACAACTTTTGATTCTTGAACTTCACTCTCGTTGG
GGAAACCGATGGTCCAAAATCGCCCAACATTTGCCTGGAAGAACTGACAATGAGATAAAAAACTATTGGA
GAACTCGTGTCCAAAAACATGCCAAGCAACTCAAATGTGACGTGAACAGTAAGCAATTCAAAGACACCAT
GCGATACCTATGGATGCCAAGGTTAGTTGAAAGAATTCAAGCCGGCATTGCCTCCTCCTCCTCCACAACAG
CCACTGCCATGGGGCAAATCGGTATACCACAAGTAGCCCTAAGTTATAACACGCTTGACAATTCCAGCACC
ACAGTCTCGTTGGAGACTCAAATTTCACCAACATCGGATTTTTCCGATTGTTACGGTAACATCGTCCCGAT
TAATGAAAACACGAACCCGGATTACTTTCAGGCTAGCCAAATTGGTTCCTCTAATGATTACAACCATGGCA
TGGATTTCCAACCATGGTTGCGTGGAGTTGATAATATTACATCAGATCATTTCTTGGATTGTGATGATTTT
TTGTTCTTACAACAGCAATTTAACTTCAACATGTGA

>GaMYB080 Cotton_A_16524 locus=CA_chr11:16316664:16317553:+

ATGTTATCTCTAAAGTTGAGATGGTTGAATTATCTTCGGCCTGATGTCCGACGTGGGAACATCACACTTGA
GGAACAACTTTTGATTCTTCAACTTCACTCGCGATGGGGAAATCGATGGTCCAAAATCGCCCAATACTTG
CCCGGAAGAACCGACAATGAGATCAAGAACTACTGGAGAACTCGTGTCCAAAAACATGCCAAACAACTCA
AATGTGACGTCAACAGCAAAGAATTCAAGGACACCATGCGTTACCTATGGATGCCTAGGTTAGTTGAAAG
GATTCAAGCTGCCACCACCACCACTGACACCGCGTCCGCTTCAGCTGCCACGACGAGCAATGCGAGCATTG
AGCAAATGTTGTTGCCTAATTTGAGTTATTACACTCTAGATAACGTTAGCACTGCTGCCTCATCGGACTCT
ATCGGGACTCAAGTTTCACCAATTTCAGATTCAACTGATTATTACAACGGTGTCTTGGCTAATTATAACCC
AAATCAGGATTATTTCCAAGCTAACCATGAATCCTTAATAAGTCCCATTGGGAATTATTGCAACAATGGCG
ATGGCATGGATTTTGGGTTGATAGAATTGAACCACCATTGGGTAGGTGATGGTGATGGTGATGGATTGCA
TAATTTATTGAACGCTGAAGATTTTTTCTTCTTACAGCACCAGTTCAACTTTAACATGTGA

>GaMYB074 Cotton_A_30606   locus=CA_chr11:10727942:10729019:-

ATGGGGAGACCACCTTGTTGTGACAAAGCTGGTTTGAAGAAAGGTCCATGGACTCCTGAAGAAGACATCA
TCTTGGTATCTTATATTCAACAACATGGTCCTGGGAATTGGAGGGCTGTTCCTACAAATACAGCGTTGCAT
AGATGTAGCAAGAGTTGCAGGCTTAGATGGACTAATTACTTAAGGCCTGGGATTAAGAGGGGAAACTTTA
CAGATCATGAAGAGAAATTGATAATCCACCTTCAAGCTCTTTTAGGCAACAGATGGGCTGCAATAGCTTC
TTACCTTCCTCAGAGAACTGACAATGACATTAAAAACTATTGGAACACTCACTTGAAAAAGAAGCTGAAG
ACGATCAATGGCAGTGAAAGTTATTCTAGAGAAGGGTTTTCATCAGAAAAACACCAAATTTCAAGAGGCC
AATGGGAGAGAAAGTTGCAGACTGATATCCAAATGGCTAAACAGGCTTTAGCTGATGCATTGTCACCAGA
GAAATCAAGTGGTTTAGCTTCAAAACCAAGAGGATATGCATCAAGCACTGAGAATATAGCTAAGTTATTG
AAGAAATGGATGAGAAATCCACCAAAACCTGTTTCAAGTGATAATATGGGAGGGATTGGGACTCCATGGA
AGGAACACAAAAGCAGCAACAGCATTGAAATGTCTGAGGTTTTTGAGTCATTGGAAGTGTTTGAATCATT
TGATTCTTCAAATTCTGATTTCTCACAATCTTTGTCACCTGATCAAGCAAGCCTTTTCCAAGATGAAAGT
AAACCAGATGTGAATGAACTAGGGCAACTCACATTGCTTGAGAAATGGCTTTTTGACGATGGTGCAAGTC
AAGGGAAAGATGACCAGCTTAGTGATATCAAACTAGATGGAAATGCTATTTTTTTCTAA

>GaMYB077 Cotton_A_36850   locus=CA_chr11:89036210:89037134:+

ATGTCATCCTTTACAAAAGTTAGTTCTAGTACAAGTTGTAGTGAAGAAGATACAGAGCAACTCAGAAGAG
GGCCATGGACGCTAGAGGAGGACTCTCTCCTAACTCATTACGTTGCTCGCCATGGTGAAGGTCACTGGAA
TTTGCTTGCAAAACATGCAGGGTTAAGGAGAACTGGGAAGAGTTGCAGGTTGAGATGGTTGAATTATCTG
AAACCAGATGTGAAGCGTGGGAACCTTACACCAGAAGAACAGTTTTTGATCCTTGAACTCCATTGCAAAT
GGGGCAACAGGTGGTCCAAGATTGCACAAGAGCTACCAGGGAGAACCGACAATGAGATCAAGAATTACTG
GAGAACCCGTGTGCAAAAACAGGCACGTCATCTTAAGATCGATGCTAATAGTGCAGCATTTCGAAATGTT
ATTAGATGTTTTTGGATGCCAAGATTGCTTCAAATGATGAAAGGGTCGTCAACACTTCCTCAACAAGTAG
CAGGGCTGGAGCAGCAAAATTCAGACTCGGAGCAGTGTGCGAGTTCATGTGTTTCTTCTTCAGAATCCAT
GAATAATAACATGTCGAAAATATCTGAATTTGCAGAGTACCAAACAAGTTCATTCGGCATCACTGATAACA
ATGAGTACAACACGGTTGCAAAGGATTGCTATTATGTTGATAACAGTAGTTGCTACGGCATGGAAACCATC
AACCTGCCATCCACATCAGCAGTTGGGGATTTTATGACTGCCGACTGCCACATGGCGGACAACAATTGGG
TTAATGATGGTTTTGCAGATGGGATATGGAGCATGGGAGAACTATGGGAACTTCCATTGAAGTGGAACCT
CTGA

>GaMYB155 Cotton_A_14866 locus=CA_chr8:31334270:31335091:-

ATGGCTTCTACGAGGAAAGATATGGATCGGATCAAAGGTCCATGGAGTCCAGAAGAAGATGAAGCTTTAC
ACCGTTTAGTACAAACGTACGGACCAAGGAATTGGACTTTGATAAGCAAATCAATTCCGGGTCGATCCGG
GAAATCATGTCGGTTACGATGGTGTAACCAGCTTTCACCGGAAGTTGAACACCGGCCTTTTACCCCCGAA
GAAGATGATACTATAATCCGAGCCCATGCTCGAGTCGGTAACAAATGGGCTACCATAGCTCGTTTACTCAA
CGGCCGTACCGATAACGCCATTAAAAATCACTGGAACTCGACGTTAAAGCGAAAATGCTTGTCGATGACC
GAAGGTTTTAACGACGATTCCCCTCAGCCGCTTAAACGATCCGCCAGTCTCGGAACCGGCAATCCAAGTAG
TCCATCGGGGTCCGATTTGAGTGACTCGAGTTTACCAGCTCCCTCACCGGCTTTCAAAACGGGTTCTTTAG
TCCCTTTGAGTCAACATGTTGAAACAGCTTCATCGGCAACGGACCCACCTACTTTACTCAGCCTCTCGTTA
CCCGGTTCTGACTTTACCGAACCTGTTTCTCCACCCGGATTCAACCCGACCCAAGTGACCACACAGGCTTC
GGCACCGGTCCCAGCTCCGGTGGAGAAGCAGTTTTTCAGTGCCGAGTTTTTTGCAGTAATGAAAGAGATG
ATAAGAAAAGAAGTGAGGAGTTACATGGCGGGGATTGAGCAAAATGAGCTTTGTTTACAAACGGAAGCTA
TTAGAAACGCTGTTGTTAAGCGTATTGGAATTAGCAAAATCGAGTAG

>GaMYB066 Cotton_A_08735 locus=CA_chr5:50574294:50575877:-

ATGGATTTGATACGCGGTCCTTGGACTGTTGAAGAAGATTTTAAGCTCATCAATTACATTGCCATCCATGG
CGAAGGTCGTTGGAATTCCCTTGCTCGTTGTGCAGGTCTGAAGCGAACTGGGAAAAGTTGCAGGCTGCGA
TGGTTGAATTATCTACGCCCAGATATTAGACGTGGGAACATCACTCTTGAAGAACAGCTTTTGATTCTCGA
ACTTCACTCTCGTTGGGGAAATCGATGGTCGAAAATTGCTCAACATTTGCCTGGAAGAACCGACAATGAG
ATCAAGAACTATTGGAGAACTCGTGTCCAGAAACATGCGAAACAGCTTAAATGTGATGTCAACAGCAAGC
AATTCAAGGACACCATGCGTTATTTATGGATGCCTAGGTTAGTCGAAAGAATCCAAGCTGCCGCCGGTTCC
ACCACCACAACCACTACTACCACCGCGGTCGTCCCCTCCGCCTCTGTTATGGGCAAAGACTTTTCGACACA
AGTAACCCCGAGTTATACTTCGGAGAATTCGAGCACGGCCACCGCGGCTTCGTCGGAGTCATTCGAGGCCC
AAGTTTCGCCGGTTTCGGATTTGACTAATTGTTACAATAATGGGTTCCAGGTTACTAATAACAATAACCCT
AATCCTGATTATAATTTCCAAGCCTGTAGCAGCCAAGTTGATTACTATGAGCACAGTCTCTCAGCTGCTAA
TTATTATAACCATGGCGATGGCGGCTGCTTGGATCTTCAGACCAACAATTATCCCATGTTAGATGGTGCTG
ATTTATCAGACAATTTGTTGAATGCTGAGGATTTTTTGTTCTTACAGCAACAGTTCAACTTCAACATGTG
A

>GaMYB069 Cotton_A_17954 locus=CA_chr5:17498490:17499692:-

ATGGGGAGAACACCTTGCTGTGACAAAAGTGGCCTCAAGAAAGGTCCCTGGACTCCCGAGGAAGATCTTA
AGCTTACTAACTATATTCAGGTTCATGGACCAGGAAACTGGCGTACTCTCCCTAAGAATGCCGGTCTCCAA
AGATGTGGGAAGAGTTGCCGTCTCAGATGGACGAACTACCTTAGACCTGATATCAGGAGAGGAAGGTTCT
CATTTGAAGAAGAAGAAACTATAATTCAACTACACGGTATCTTGGGGAACAAGTGGTCGGCTATTGCGGG
TCAATTGCCAGGAAGGACCGATAATGAAATTAAGAATTACTGGAACACCCATATCAGGAAAAGGCTTCTA
AGGAATGGGATCGATCCAGTGACACATGCTCCACGTCTTGATTTGCTTGACCTTTCTTCCATTATCAGCTC
AACTTTGTGCAACCAATCTCTTCTCAATGTGTCAAACTTGTTAGGTACACAAGCTCTCCTAAATCCCCAAC
TGCTCAGCCTAGCAAACACCCTCTTGTCTCTAAAACAAGAAAACCCAGAAATGCTGATGCAATATCTCCAA
CAAAACCAACTAAATCTAGACCCCTCGTTACAAGCTCCCATTCAAACTGCTTCAGCTTGCACCACCACATC
CACCGTCCCTTGTTGTACTTCGATGCTAAGCCAAACAGGCCTCATACAAGCCTCCGGAGAAGGCTTTTTCT
CTTCCGATATGACGAACTTCAGCTACCCAAATTCCCAAGAAATTTTGACAACTTCCACTTTAACCAATGAA
TTTGCTGCGCAACCACATTACGTGCAGTGCAGCACAAATCCTACAGTTCCCATTCTGTCTGAAACTTCCAA
TTTCCAATCAGTGGATGGCAGCTTTGATTCGGTGAGGTCGTCGGCGATATCAAGTCCAACGCCTTTGAAC
TCATCATCTACATTCGTGAACAGCAGCAGTACCGATGACGAGAGAGAAAGCTTTAGCAGCTTGCTTAAGT
TTGAAATACCGGAGGGCTTGGATATCAATGATTTTATGTAA

>GaMYB020 Cotton_A_05930 locus=CA_chr1:140699894:140701027:-

ATGGGAAGACAACCTTGTTGTGACAAAGCTGGTGTCAAGAAAGGGCCATGGACTCCTGAAGAAGATCTCA
TCTTGGTGTCTTATATTCAACAACATGGTCCTGGGAATTGGAGGGCTTTTCCCACCAAAACAGGTTTGCT
TAGATGTAGCAAGAGTTGCAGGCTTAGGTGGACTAATTACTTACGGCCTGGGATTAAAAGGGGTAACTTC
ACAGAAAATGAAGAGAAAATGATAATCCACCTTCAAGCACTCTTAGGCAACAGATGGGCTGCAATAGCAT
CTTACCTCCCTGAAAGAACAGACAATGACATCAAAAATTATTGGAACACTCATTTGAAGAAGAAGCTGAA
GAAGCTACAAGGCAATGAAGGTTCTTCTAGATATGGGTTTTCATCATCATCATCGTCATACCAGATTTGTA
GAGGTCAGTGGGAGAGAAAGTTGCAGACTGATATCCATATGGCTAAAAAGGATTTATCTGATGCATTATC
ACCTGAGAAATCAAGTGACCACACATCTTATCCAAAACCAAGTGGATATGCTTCAAGCACTGAAAACATA
GCTAAGTTGTTGAAAGGGTGGATGAGAAATAATCCATGGAAAATGGCTGATTCTACAGATTATAGTGAAG
AAGGGACTGATCCAATGAAGGAAGACAAGAACAGCAAGGAAATGGCAGAGGCATTTCAATCACATTTAG
GGTTTGAATCTTTAGATTCTTCACTTTCTGATATCTCACCATCTATGTCACCTGAGGCAAGCCATTCACAA
TATGAAAGTAAGCAACACGTCAATGCTCAAAGCCAACTTTCAATGCTTGAGAAATGGCTTTTTGATGAAG
GCAAAGATTACCAACTTTGTGACAATATATTAGACCAAAATGTTCTTTTTTTCTGA

>Cotton_A_27019 locus=CA_chr1:109655118:109656188:+

ATGAGAGCAGCTATGCCTCCTATTAATACAAAGAACATGTCTACTCCTCTAGAAGAGGAGGAAAGTGAGCT
GAGAAGAGGACCATGGACCCTTGAAGAGGATACTCTCCTAACACATTACATTGCTCGTCATGGAGAAGGG
CGTTGGAATATGTTGGCAAAATGTGCAGGTCTCAAGAGAACTGGCAAAAGTTGCAGGCTAAGGTGGCTGA
ATTATTTAAAACCAGACATTAAGCGTGGAAACCTGACTCCCCAAGAACAGCTCTTGATTCTTGAACTTCA
TTCCAAGTGGGGAAACAGGTGGTCGAAAATTGCACAGCATCTTCCTGGAAGAACTGACAATGAGATCAAG
AACTACTGGAGAACAAGGGTGCAAAAACAGGCACGCCAACTGAATATCGAGTCCAACAGCCAGAGATTCC
TTGATGCGGTTCGATGTTTTTGGATGCCAAGATTGGTTCAAAAAGTGGAGCAGGCCTCACCATCTTCCTC
CTCCTCCTCTTCTTCTTCTTACTTGAAAGAAATGTGCACCCAAAGCTCAGTTCCTTCTCAATTATCTAGCT
GCTGCACAGTCCCTTCATTCCCAACATTTTCCCCACTTGCGAACAAAACCACAGACATTTCAAATTCAAGT
TCAGTCACCACCCAGAACATTTGTCCCACAGATTCCATCAACATTTCGGACCAAACTGAAATTCCCCAACA
CCTAACAGGTTCCAACGTATATGGCCATACAACTCTTGACCAATGTTACAATATCGACAGCAATGATTATG
GCATGGAGGGTATCAGCTTTGCATCTATGTCAGCTGTAGGCTTCTATGAAGGAACCTCATCTGAAGGCAAC
TGGATGTGCACTGAGATGAGTGATAATTTATGGAACATGGATGACATATGGCAGCTTAGATCTATGTAA

>GaMYB007 Cotton_A_29987 locus=CA_chr1:145221464:145222644:+

ATGTTATCCTATACAAATTCTAGTAAAGGCGATGCTGAGCAGCTTAGAAGAGGAACTTGGTCTGCTGAGG
AAGACTCTCTCCTAATTCATTATATTGCTCGCCATGGCGAAGGCCACTGGAATTTGCTCGCAAAACGAGCA
GGGTTGAGGAGAACTGGAAAGAGTTGCAGGCTGAGATGGCTAAATTATCTGAAACCAGATGTTAAGCATG
GAAACCTTACTCCACAAGAAGAGTGTTTGATTCTTCAACTCCATTCCAAGTGGGGTAACAGGTGGTCAAA
GATTGCAAAATATTTACCAGGAAGAACAGACAATGAGATAAAAAACTACTGGAGAACCCGTGTTCATAAA
CAAGCACGTCATCTAAAGATTGATGCTAATAGCACACCATTTCAACATGTTATTCGATGTTGTAATTGGAT
GCCAAGACTGCTTCCAAAAATGGAATCACTAAACCAAACTTCTCAGCATGATCAAGAATTAATGACAATG
GAACAAGTGCCAGTACAGGAGCAGATTTCAGGATCTACGGATATCATGTCAACCATATCCCAAGTTGCAGA
GTCTCAAACAAGTCCTTTTAGCATCATTAGTAGCAATGATGACTACACACTTGCAAAGTATTGCTACCATG
ATGACAATATTGATAAAAACGGTTGCTATTACAACTTGGCATCCACATCAGCATTTGAGGATTTACCATAC
CTGGATGGCGACGGCCACAAGCAAGATTATACTTGTGTTAATGATGGCTTTGCAGATGGTCTATGGAGCAT
GGGGGAACTATGA

>GaMYB180 Cotton_A_14958 locus=CA_chr12:106046280:106047356:-

ATGGGGAGAACACCTGGCTGCGACAAAAATGGCCTCAAGAAAGGTCCCTGGACTCCCGAGGAAGATCTTA
AGCTTATTAATTATATTCAGATTCATGGCGCCGGACATTGGCGTAACCTCCCCAAGAATGCTGGGCTCCAA
AGATGTGGAAAGAGCTGCCGTCTCCGTTGGACGAACTACCTGAGACCTGATATAAAGAGAGGAAGGTTTT
CATTTGAAGAAGAAGAGACTATTATTCAACTACACAGTGTTTTAGGAAATAAGTGGTCCGCCATTGCTTG
TCGGTTGCCGGGGAGGACTGACAATGAGATCAAGAATTACTGGAATACACATATAAGGAAAAGGCTTGCA
AGGAATGGAATCGATCCTGTAACACATGCTCAACGTCTTGATTTAGTTGACTTGTCTTCTTCCATTATTAG
CTCATTATTAGGTGTCCAAGCTCTCCTAAATCCTCAACTGTTAAGCTTAGCTAACACCCTCTTGTCGTTAA
AACAAGAAAACCCAGAATTATTACTACAATATCTTCAACAAAACCAGCTTCTTCAAACTCCCACGTTAGAA
CCACTAAGCCAACTTCTTCAAGCTCCCATGACAGACTGTAGCGATCAAAATTCCCAGAAAAGTAATGTATT
AGTTTCGCAACCAAATTATGAACATTCTAATCCAAATCCAACGGTACCATCACTTGTATCTGACAATTCAG
ATTTCCATTCAATGGATGGTAGCCAGAATTTTGGGTTGGATTCAGTGAGGTTCACGCCGATATCAAGTCC
AACTCCGTTGCATTCATCATCTACATTCATCAACAACAGCTACACTACCCATTACGAAATAGAAAGATTCA
ACAGTTTGCTCAAATATGAAATTCCAGAAAGTTTGAATATTAATGACCTTTTGTAA

>GaMYB049 Cotton_A_19829 locus=CA_chr12:45215969:45216975:+

ATGGATGTTTACGAGACTGGTTTCATATCTGAAACCCCACAAAGTGAAGAAGATATGGACGTAAAGAAAG
GTCCATGGACTGAAGAAGAAGACTTCACGCTTAAAGCTTATGTTAATATCCATGGCGAAGGTCGCTGGAA
CTCAGTTGCTCGCTTATCAGGATTGAAAAGAACCGGCAAAAGCTGTAGATTAAGATGGCTGAACTATTTG
AGACCGGAAGTGAGACGTGGGAACATTAGTCTCCAAGAACAGCTATTGATTCTTCAACTCCATTCTCGAT
GGGGCAACAGATGGTCTAAAATTGCACAACATTTGCCTGGAAGGACAGACAATGAGATAAAGAACTATTG
GAGAACCAGAGTCCAAAAACAGGCGAAACAGCTTAAATGCGACGTTAACAGCAAGCAATTCAGGGACGCC
ATGCGGTACGTTTGGATCCCTCGTCTGGTCGAACAAATCTGCGCTTCATCCGGATCCCACTCGGCTCAACA
ATCCTCCTCCACCACCACTACCTACGCAGATACGATCGGTTCGGTCCGAGTTGATCCGAGTCTCTTGCCCG
AGTTATCAGGCACTTCATCAGATTCCCTGGACGCCCAAGTCTCTTCCGTTTCAGACCTGACAAATTCAAAA
TACCCGAATAGCTTACAAAACGGGTCGGGTTCAGGAAATACAATAGCGGGAACATGGGGTGGGGTTGAAA
TTGAGGCGACGGACGGTGGAGATTCGATGGAGAGTGTGTGGAACGAAGAGAATATTTGGTTCCTAAGACA
GCAGCTTTATGATGACGATGATTTGAATTAG

>GaMYB050 Cotton_A_19831 locus=CA_chr12:45164419:45165370:+

ATGACTCCACAAAGTAAAGAAGATATGGATGTAAGGAAAGGTCCATGGACTGAAGAAGAAGACTACATGC
TCAAAACTTATGTCAATGTTCATGGTGAAGGTAGCTGGAACTCAGTTGCTCGCTTATCAGGATTGAAAAG
AAGTGGAAAAAGCTGCAGATTAAGATGGCTGAACTACTTGAGACCAGAAGTGAAACGTGGGAACATAAG
TCTCCAAGAGCAGCTTTTGATTCTTCAACTTCATTCTCGATGGGGCAACAGATGGTCAAAAATTGCACAA
CAATTACCCGGGAGGACGGACAATGAGATAAAGAACTATTGGAGAACCAGGGTCCTAAAACAGGCAAAAC
AGCTTAAATGCGACGTTAACAGCCAAGAATTCAGGGACACCATGCGTTGCGTTTGGATTCCTCGTCTGAT
TGAACGGATCTGTGCTTCATCCGGATCCCCCTCGGCTCAACCCTCCACCGCCTATGAAAAGACGAGTCTCC
TGCCCGCGTTATCAGGCACTTCATCAGACTCCCAGGACGCCGAACTCTCCTCCGTTTCAGACCTCAAAGAT
TGTTACAACCCACCAAGCAATTCAAACAACCCGAATAGCTTACAGAACGGGAGGGGTTTATGGTCGGAAA
ATTTAGGCGGCACTTGGAATATAGAGGATGGGGTTGGATTTCAGGCGACGGAGGGTGGAGAGTCGATGGA
GAGTGTGTGGAACGAAGAGAATATTTGGTTCCTAAGACAGCAGCTTTATGATATGATATGA

>GaMYB177 Cotton_A_19852 locus=CA_chr12:43954511:43955656:-

ATGGGGAGACCACCTTGCTGCGACAAAGTCGGTGTGAAGAAAGGTCCATGGACTCCTGAAGAAGATATCG
TCTTGGTGTCTTATATTCAAGAACATGGTCCTGGGAATTGGAGAGCTGTTCCTACCAATACAGGGTTGCTC
AGATGTAGCAAGAGTTGCAGGCTTAGATGGACTAATTACTTGAGGCCCGGGATTCGAAGGGGTAACTTTA
CAGAACATGAAGAGAAGATGATAATCCACCTTCAAGCTCTTTTAGGCAACAGATGGGCTGCAATAGCATC
TTACTTGCCTCAAAGAACAGACAATGACATTAAAAACTACTGGAACACTCATCTGAAGAAGAAGCTGAAG
AAACTCCATGGAAGTGAAGGTTATTGCAGAGATGGGTTCCCATCATCAGCTGCATCAGACCAGATTTCAC
GAGGTCAATGGGAGAGAAAGTTGCAGACTGATATAAATATGGCTAAACAGGCTTTATCAGATGCATTGTC
GCCTGAGAAATCAAGTGATTTAGCTGAGTTGAAACCTTGTCATGGGAACACTTATGCCAAACCAGAAGGG
TATGCATCAAGCACAGAGAATATAGCTAAGCTGTTAAAAGGGTGGATGAGAAAGAACCCATTAAAGCCTG
CTTCAACAAACTCTGGTGTCACTCAACAATCATATGACAACATGGCGGCTACTGGGGTGACCACCGATTCA
GCTAATTCTAGTGAAGGGAATGATCAAAGGTGCAGCAAGCCAATATGTGAGGGTTTTGAGTCATTGTTTG
TGTTTGAATCTTTTGATTCTTCAAATTCAGATGATTTCTCACAATCCATGTCAACTGAGGCAAGCCTTAG
TTTGCAAGATGAAACAAAGCCAGATCTTAGTCCCCAACTTTCATTGCTTGAGAAATGGCTTTTTGATGAT
GCTGCAAATCAAGGGAAATATTACCAGCTTAGTGATATCACATTAGATGAAAACCCTAGTTTTTTCTAG

>GaMYB151 Cotton_A_20268 locus=CA_chr12:67440779:67443791:+

ATGAGTCACACAAAAAATGAGAGGGAAGATGGAATGCTCTCAAAGAATCGGAAAGAATTGCCGTTGATT
GATGATGGAAACTATGGAGATGCAGATGGGGGAGCTCTTCTGAAGAAAGGACCATGGACGTCTGCTGAAG

ATGCAATTTTGATTGACTATGTGAAGAAGCATGGAGAAGGCAACTGGAATGCTGTTCAGAAGCACTCTGG
ATTGTTTCGTTGTGGAAAAAGTTGCCGCTTACGTTGGGCAAATCACTTGAGGCCAAACTTGAAGAAAGG
GGCATTTACACGAGAAGAAGAGCATCTGATTATTGAACTCCATGCAAAGATGGGAAATAAATGGGCTCGG
ATGGCTGCAAACTTGCCTGGTCGCACAGATAATGAGATAAAGAATTATTGGAATACCCGGATTAAGAGAC
GTCAACGTGCTGGATTACCTCTGTATCCTCCTGAAGTGTGCGTGCAAGCACTGAAGGAGAGCCATCGTACC
AGTGCAATTAGTGGAGGGGATAAAGGAACTCATGACATCTTGCAGAACAACACCTATGAGATACCTGATG
TCATATTTGACAGTTTAAAGGCCAATCAGAATGTCCTGCCTTATGTCCCTGAACTTCCTGATATATCTGCT
AGCTGCATGATGATGAAAGTTCTAGGTTCTTCCCAGTATAGTAGTTTCATACCACCAGCAATTCATCGCCA
GAAGCGTCTTCGAGAATCAGCTGCCTTTTCCCCTGGTTACACTGCTGCTGTTAAAAATGACTGCCCCTTG
TTTGAGCAGTTTGAAGACAATATGTTCGACAAGGCTGCTCAACCCTTTGGGCAGTCTTTTCCATTTGAAC
CAGATCCTTTAACTAAAAAATCCCAGTCATTTAGTGCATTCCAAGGTAGCCATGCCCTTTCCCATGGCAAT
TTCTCTGCTTCAGAGCCCACTTTGGAGGCTGTGAAGTTGGAGCTCCCTTCACTCCAATATCCTGAAACTG
AATTAGAAAACTGGGGCACGTTATCTTGCTCCTCATCTTTACTTGAGTCTATTGATGCTTTTATCCAGTCA
CCACAAACAACCAGCGGAGTGGTCTTGGATAGTCTTTCACCCCGTAATAGTGGCCTGCTGGATGCTTTACT
CCATGAGGCAAAAACTCTAAGCTCTGCAAAGAACCATGCATCTGACAAGTCTTCAAATTCATCAACTCCC
TGTGATATAGCAGAAAGGTCCAATTTCAACACTTGCGAGACAGAATGGGAAAATTATGGTGAGACTCTTT
CTCCTATGTGTCATTCAGCAACTTCTTTCTTCAGTGAAAGTATCAGTGCAAGTGGCAGTTCATTGGATGA
ACAACCACCTGCTGAAACCTTTACTGAGCCTCATACAAAATCAGAATCATCGGATCATGTTTTGGCCCCAG
AGGTGGAAAAAGAGGCTCCTATTTGGTCAGACGGTAGATGCCCTGATACTTTGTTTGCTTCAAATTGGCT
CGAGCAGGGTTCTGGTTATGATAAGGACCAGACAATCATGATTGATGCCATAGCAACCCTTCTCGGTGATG
ATCTGAGTAGTGAGTACAGGAGCATGTTAGCCGGAACATCAGTTTCAAGTCAAGCGTGGGAACCCAGCTG
GTGTGCATGGAATAACATGCCTGCTGTGTGTCAGATGACTGAACTTCCTTGA

>GaMYB178 Cotton_A_23436 locus=CA_chr12:81322816:81323553:+

ATGTCCATGAAAAAAGAAGGTGAAATTCTATACAAAAAGGGATTATGGGCAATGGAGGAAGACAAGTTAC
TCATTGATTATGTCAATGTCCATGGAAAAGGACAATGGAACAAAATAGCCAACAGAACAGGTTTGAAGAG
AAGTGGGAAAAGTTGTCGGCTAAGGTGGATGAATTACCTGAGTCCTAACGTTAAAAAGGGTGATTTTTCT
GAAGAAGAAGAAGACCTCGTCATTAGACTTCATAAGCTTCTTGGAAACAGGTGGTCTTTGATTGCGAAAC
GAGTTCCAGGTCGAACTGACAATCAAGTCAAGAATTACTGGAATAGTCATTTGAGGAAGAAACTAGGGAT
CATTGATCAAAACAAGACAAGGATCGATTTTTGTCAAAGTTCAAAGCAAGTCAAAGTGTGTCATGTTGAT
GAGGCAGCCACGGATCCAAGTCCTGGACATGGAACAACCACTGAAACCACGGGTATAACAGTGGATCAGA
GTAACCAGCAGGAAGTCATTGATCATCGGGTCTTAAACAATACTACTCAAGAATCAATGACCACTGAGAG
TTATATCAACACTTTCTGGATTCCTGACCATGATTATGAGCTAAGTACACTTGCCATGATTGACCACTTCC
ATGAATGTTCTTCTTTTCATCTTAGCTAG

>GaMYB105 Cotton_A_18881 locus=CA_chr4:124244912:124246128:-

ATGGGAAGACCTCCTTGCTGTGATAAAGTTGGTATCAAGAAAGGTCCATGGACCCCTGAGGAAGATATCA
TACTTGTCTCTTACATCCAACAACATGGTCCAGGGAATTGGAGATCAGTACCCACTAATACTGGGTTATTG
AGATGCAGCAAAAGTTGCAGGCTTCGATGGACTAATTATCTGAGACCAGGCATCAAGCGAGGGAACTTCA
CTCCTCATGAAGAAGGCATGATAATTCACTTGCAAGCTTTATTGGGTAACAAATGGGCAGCAATTGCTTC
ATACCTACCACAAAGGACAGATAATGATATAAAGAACTACTGGAACACCCACCTGAAGAAGAAACTAAAC
AAGTTTCAGTCAGCTTTGGACCCCCACCCTTCAATTGAGAGAAAGAGCAGCTTAGAATTTGCTTCAACTT
CTTCAAGTACTAGTGCTCTTGGGCTAAATCAAGTCTCTTCAGCATATGCTTCAAGCACTGAGAATATTTCT
AGGCTTCTTCAAGGTTGGATGAGATCATCACCAAAGATAACTAATGATAATAATAGCTCTCTACCTCTCAA
GGATACTTCTCAATATCAGAAATGGGATCAAAATACCAGCTTAGATGCTGATTCTTCAGCTGGATATAAAC
CCAAAGCTGAACAAGAAGGCGGCAATTTGATCTCCCATGAAGAGTTTGAGTCGATTTTATCGTTTGAGAA
CATTAACAATGTTGTATGGGACAAGTCTACTTGTGAATCTACATCAAAGGGTGCTTGTCAAGATTCAGGA
AATGACGATGATGGTGATGATGATGATGATGGTGGTGGTGGTGGTGGTGGTGACAAGGTCAATGTTG
CAATGGCCCCAGAGAATTATAAGAAGCAAAAAGTTGATCATCACAACAACAATAATAAGAATAATCCGCCA
TTATCGTTTCTTGAGAAGTGGCTTTTTGATGAGAGTTCTTCAGGTCAAGTAGAAGAGATGAATCAGATGA
TGGAACTGTCTTCAGTCTTCTAA

>GaMYB097 Cotton_A_23218 locus=CA_chr4:135539006:135540069:-

ATGGATGTTTACAAGAGAGGTTTCATATCTGAAACTTCAAGAAATGAAGAAGAGATGGAGCTAAGGAAAG
GTCCATGGACTGAAGAAGAGGACTCCATGCTTAAGGCGCATGTCAATATCCACGGCGAAGGTCGCTGGAA
CGCTGTTGCTCGCTTATCAGGATTGAGAAGAACCGGAAAAAGCTGCAGATTGAGATGGCTGAACTATATG
CGACCAGAAATCAAACGAGGAAACATCAGCCTCGAAGAGCAGCTATTGATTCTTGAACTCCATTCTCGCT
GGGGCAACAGGTGGTCGAAAATTGCACAACACTTGCCTGGAAGAACAGACAATGAAATAAAGAATTATTG
GAGAACAAGAGTCCAGAAGCAGGCCAAGCAGCTTAACTGTGACGTTAATAGCAAACAATTCAAGGACGCC
ATGCGTTACGTTTGGATCCCCCGTTTAGTTGAACGGATCCGTGCTTCATCCGAGTCTCCCTCGAGTCAACC
TTCTACCACCACCAAAACCACCTACAATGATTGTATCAGCAACATAAGCAGCAGTCAAATGAGCTACGCAA
ATGCGAGTGGGTCGGTCCAGGTTGACCCAAGTCTTCTGCCCGAATTATCAGGCACGTCATCAGACTCCCTT
GACACCCAAGTCTCTTCCGTTTCGGACCTGACCGATTGTTACAATCCACAGAGCCTTTCAAACTACTTACA
CAAGGGGTTAGGTTTGGAAAAGGAGGGAGCGGCCACATGGGGCAGAGATGAAGAATTCCAGGCGACAGA

GGAGCATAGCAATGGTTTGTTGGTTGGTGGTGGGGAGTCATCGATGGACACTGTGTGGAATGAAGAGAAT
GTTTGGTTTTTACAGCAGCAGCTTCATGATGGGATCTAA

>GaMYB102 Cotton_A_32784 locus=CA_chr4:88681417:88682316:-

ATGGATCGGATCAAAGGTCCATGGAGCCCCGAAGAAGATGACTTGCTCCAGCAGCTGGTACAGAAACATG
GCCCCAGAAACTGGTCTTTGATCAGCAAATCAATCCCCGGCCGATCCGGTAAATCCTGTCGGCTCCGATGG
TGCAATCAACTGTCACCGCAAGTTGAGCACCGTGCCTTCACCCCGGAAGAAGACGAGACCATCATCCGAG
CACATGCCAGGTTCGGTAACAAGTGGGCCACCATAGCCCGACTCCTCAACGGCCGTACCGACAACGCCATT
AAAAACCACTGGAACTCCACGCTAAAACGTAAGTGCTTGCCGGTTGGGGAAGAGTGTAATTTCGTTGCTA
ATGGAGGGTATGATGGTAATCTGGGAGGAGAGGAACGGCAACCGTTGAAAAGATCGGTGAGTGCTGGTCT
ATACATGAGTCCAGGGAGCCCATCGGGATCGGATGTGAGCGATTCTAGTGTTCCCGTCTTATCATCTTCTT
ACGTGTACAAGCCGATCCCAAGGACCGGCGGTGTTAACGTTGATGTAAATGTTACGCCAGCTGGAGTGGA
AGCGGCATCATCTTCCAACGATCCACCGACCTCACTGAGTCTGTCTTTACCGGGGGTGGAGTCATGTGAGG
TGGTGTCAACCCAGCCAATAACGGAGTCAACTCAGAATCGGAGTGAAGAAAGGGGAGGTGGGGTGATGG
GTTTCAGTGCGGAGTTTATGGCGGTGATGCAAGAGATGATAAGGGTTGAGGTGAGGAATTACATGACGCA
GATGCAGCAACAGCAGCAGCAGCAAAACGGCGCAGTTTCGGGAGGAGCGGGAATGGGGATGTGTTTGGA
TGGGGGGTTCAGGAATCTTATGGCTGTGAACCGAGTCGGGATGAGTAAGATCGAGTGA

>GaMYB183 Cotton_A_11755 locus=CA_chr7:106053392:106054475:+

ATGGGGAGGTCACCATGTTGTGAGAAGGTAGGGTTGAAGAAAGGTCCATGGACCCCAGAAGAAGATCAA
AAGCTCTTAGCTTACATTGAACAACATGGCCATGGAAGCTGGCGTGCCTTGCCTTTAAAAGCTGGGCTTC
AAAGATGTGGAAAGAGTTGCAGACTGAGATGGATTAACTACTTGAGACCTGATATCAAAAGAGGAAAGTT
CAGTTTACAAGAAGAACAGACCATTATTCAACTCCATGCCCTTCTTGGAAACAGGTGGTCTGCCATAGCTA
CTCATTTGCCGAAAAGAACAGACAATGAGATCAAGAACTACTGGAACACACATCTAAAGAAAAGGCTAAC
CAAAATGGGGATCGATCCTGTCACCCACAAGCCTAAAACCGATGCACTCGGCTCCACCACTGGTAACCCTA
AAGATGCTGCTAACCTTAGTCACATGGCTCAATGGGAGAGTGCTCGTTTAGAAGCTGAAGCTAGACTGGT
TCGTGAGTCCAAGCTAGTTCCTTCAAACCCTCCTCAAAGCAACCATTTCACTGCCGTTGCGCCTTCGCCGA
CTCCGGCAACTAGACCGCAATGCCTCGACGTACTCAAAGCATGGCAAGGTGTCGTCTGCGGGTTATTCACT
TTCAACATGGACAATAACAACTTACAGTCCCCTACGTCAACGTTGAACTTCATGGAGAACACCACAACATT
GCCCATGTCATCATCATCGTCTGTTAATGGAATGTTTAATGAAAACTTTGGTTGGAACTCATCGATTAATC
CATGTGAAAGTGGGGATATTTTGAAAGTTGAATATGGCAGTGATCAAATTCCAGAGTTAAAGGAAAGATT

GGATCATCCAATGGAATTGCATGAAATGGACTATTCTTCAGAGGGTACATGGTTTCAAGAGTTGTTTGGA
TTTAATGGTTTATGA

>GaMYB199 Cotton_A_15547 locus=CA_chr13:90419177:90421878:+

ATGATGGGAAACAACAACAACAACAACCACCACCAAGTAAGTATGCAGAAAGAGGAAGGTGTGATGCTGA
AAAAGGGACCGTGGACGGCCGCAGAGGATGCGGTGTTGGCGGAGTACGTGAGGACTCATGGAGAAGGGA
ACTGGAATGCAGTGCAAAGGAACACAGGGTTGGCTCGTTGTGGGAAAAGTTGTAGGCTAAGATGGGCTA
ACCATTTAAGGCCTAACTTGAAGAAAGGAGCTTTTTCTCCTGAAGAAGAAAGGATTATTGTGGAGTTGCA
TGCTATCATGGGAAATAAATGGGCTCGTATGGCTGCTCGGCTTCCAGGAAGAACGGACAATGAAATCAAG
AACTATTGGAATACAAGGGTAAAAAGAAGGCAACGGCAAGGTCTCCCACTTTACCCTCCTGAAATCCAAT
CCTTGTATTCTCAGACTCAGCCTGTACTAACACCATCTTCAACCTCCTCTTTCTCCTTCCAACATTCCACCA
CCATGCTAACATCATCATCACCATCTACGCCGCCACGACTTCTTGCCTACGATCCACATTCAGCCGCAACGC
CACCGCCTGTCCAAAGTCCAAGTCCAGCCTCAACGCCGCCCCCTCTTCCAAGCCCTTCCCATGTCTCTCCA
CTTCAATCTCCCCATAAGCCTTCATTTTCATCTATCCCTCTAGCCGATTCTTTCACATCCAACACTCCATCT
TCCTCCCTCGACTTTTACTTCCCTAGACCTTTGCCTTCCTTGCAACAGCCCCTTCGTAACAAGCGACCAAG
ACACGACGAAAACAACACCAACAATGGGGGTTCGAGCTTTATGTTACCCTTTTCATCGTTGATGAAAACG
GATCCTTTCAATCCCCACACCGTGACTAATAACTCGTTGAACCCTCAACATTACTCCAACTCATATTGTTT
GGATCAAACCACATTTGACATGGCTTCATCTTCAAGGTTCCCCCAACCCCATTTCGATCCGGGACAGTTTA
TATCGACTCCCAGATTCGGTTATAATCAGTTGAAAACCGAGCTCCCTTCAAACCAAATTTTCACCCAAGTT
GGTAATTCACAAGTTAGACTTGATCCTAAAGGGAATAACAATTACAGCAGTCATAATCAGATTCAGAATAG
CAACCACCATAGCTCGAGCTTGAATATCAGTGGGAATGGCTTGATAGAGGATATGTTACAGGAGGTTCAG
ACGTTGAATGAGAAGAATGAAATTATGGCGAGTCAGAGTTGTTTAGTTAGATCAAGTTCAAGCTCCGAGG
GTTTAGCTTCAGGGTTGGAAGCAAAAGAAGAATCAAAAGAACAAATTATGAACACAGCAAAACATGTAGA
TTACTCCAACGTAAATCCATCGTCCATGGCGATGGCTATCCCCGAATGGTACATCAACAACGGCGAAACTT
CCATCGGCCAATCATCGACTGTAACATATGATAATTTAGCGCTTGAATTCGAATTGCATGATCAAATAGCT
TCGTTATTGCCCATCGACACTGCACCCGACCACCACGGTCGATCTTCCAGCTCCTATACTTGGGATCATAGC
TTTCCTGGAATTTGTTAA

>GaMYB201 Cotton_A_22356 locus=CA_chr13:117716152:117717271:-

ATGGGAAGACCTCCATGTTGTGATAAAGTGGGTATCAAGAAAGGTCCATGGACTCCTGAAGAAGATATCA
TCCTCGTCTCTTACATTCAAGAACATGGTCCTGGAAACTGGAGATCAGTCCCTACCAATACTGGGTTAATG

AGATGCAGCAAAAGTTGCAGACTTAGATGGACTAATTATCTCAGGCCAGGAATCAAAAGAGGCAACTTCA
CTCCTCATGAAGAAGGGATGATTATTCACTTGCAAGCATTACTGGGTAACAAATGGGCAGCCATAGCATCG
TATTTACCACAAAGAACAGACAATGACATAAAGAATTACTGGAACACCCATCTGAAGAAGAAGCTAAGGA
AGTTTCAGTCAGCTATGGAGCCTCCTTCAATGGCGGGTGATGAGAAGAGCTTAGATTTCGCAGAGAGTGA
GACTGGAGCTGGTTCAAGTAATCTTAAGCTAAACCAAACCTCCTCTTCATATGCTTCAAGCACCGAGAATA
TATCTCGTCTCCTTCAAGGTTGGATGAGATCGTCACCCAAGATTAACAACAACAACAACAACAGCGACAG
CATTGGGAGTTGTTCAGCTGCTGCAAACAATGATTTGATCTCTCATGGAGATGATCAGTTAGAATCGATT
CTATCGTTTGAGAACATGAAGGATGTTGCATTAATGGAGAAATCTACTTCAAAGGCTACTTTTCAAGATT
CTGGTGAAAAGATCATTAACCCAGAAAGGAAGCAAAAAGCTGATAACAATAACAATAATATTGATGATAA
TAATAATAACAACAGTAATCCTCCATTATCGTTTCTTGAGAAGTGGCTTTTGGATGAGAGTTCTTCTGCAG
CTCAAGTCGAAGAAATTGATCAGATGATGGAATTGCCTTCAATTTTCTAA

>GaMYB192 Cotton_A_28778 locus=CA_chr13:123886772:123887846:+

ATGGGAAGATCTCCTTGCTGTGATAAAGTTGGCATCAAGAAAGGTCCATGGACCCCTGAGGAAGACATCC
TTCTTGTTTCTTACGTCCAAGAACACGGACCTGGGAATTGGAGACTAGTGCCCACTAACACTGGGTTGCA
GAGATGCAGCAAAAGTTGCAGGCTTCGATGGACAAACTATCTGAGACCAGGAATCAAGAGAGGGAACTTT
ACACCTCATGAAGAAGGGATGATTATTCATTTGCAAGCTTTATTGGGTAACAAATGGGCAGCCATTGCTT
CATATTTACCACAAAGAACAGATAATGATGTAAAGAACTACTGGAACACCCACTTGAAGAAGAAGCTAAA
GAAGTTTCAGCCAGCTATGGAACTCCCCCAGATGGCTCAGGCTTGCTTGAATAAAACCCTAGCTACTGGTT
CAAGTTCAACCATTGCTACATATGCTTCAAGCACTGAGAATATTTCTCGTCTTCTTCAAGGTTGGATGAGA
GCATCACCAAATATTTGCAGTAATCAGAGTGCCATAATTCTCCAAACCCAGTCGAAAGATGAAAGTGGAG
GTGGTGATTTGATGTCGAACGAACATTTTGAGTCCATTTTATGGTTTGAGAACATGAATAATGTTGCATG
GGAGAAATCCGCTTGTGGTTCTACTACTTCAAAGGGTGGTTTTCAAGATTCTGGGAATGATGATCAGTTC
AGTGTTGAAATTACCCAAGAAATGAAGCAAAAAACTGGGAATAATAGCAAATTTTATCCTTCATTTTCAT
CTCTTGAGAAATGGCTTTTGGATGACAGTTGTGCAGGTCAAGTTGAAGAGATGAATCAGTCGATGGAACT
TTCCCCAATTTTCTAA

>GaMYB163 Cotton_A_31426 locus=CA_chr13:28599638:28600754:+

ATGGGAAGAGCACTTTGTTTTGATAAAAATGGGCTCAAAAAAGGGCCATGGACTCCTGAAGAAGATAAA
AAATTGATTGATTATATTCAAAAACATGGTTATGGAAATTGGAGGACACTTCCAAAGAATGCTGGGTTGC
AAAGATGTGGAAAGAGTTGTCGTCTTCGATGGACTAACTACTTGAGGCCTGATATCAAGAGAGGAAGGTT

TTCTTTTGAAGAAGAAGAGACTATAATTCAGCTACATAGTATATTGGGCAACAAGTGGTCTGCCATTGCTG
CTCGGTTGCCCGGAAGAACAGATAACGAAATAAAAAACTATTGGAACACACACATTAGAAAAATTCTTCT
TCGAATGGGGATCGATCCGGTGACCCATACTCCTCGACTGGATCTTCTCGGTCTCTCTTTCCTTCACAACC
AATATCTTATCCAGTCTTTTGCTAACCCTGAGCTTCTTCGTAATGTTGACCAATATCAACCATTGATGCAA
TCTAACCATTTCCATGCACAAGTCGAAGAAACCCCAACTTGTAGTGTTCCATTTTATAACGAAGAACAACA
ACTCGTGATGAAACCCAACTTGAACCAATTCCCATCAAATTTCAATGACTATGTTATGCCTTCAATTTTGA
CTGAAGATTACGTGCCGCTACCGCCGCAGTACAACTATTACGGCTCGGATTATCAAACCGTAATGGACCCT
TCAATCGAAACCTCGAATTTCCATTCCATCAACAGCAACCAGAGCTTGGGGTTGGGGTCAGTTGTATCGA
CGCCTTCGTCGAGCCCCGCCCCATTGAATTCAGACTCCGCATACCTCAACAGTAGCAGCACCACTGAAAAC
GAAACAGAAAGCTATTGCACCAACATTTTGAAGTTGGAAATCCCTGATATTTTGGATGTTAATGATTTCA
TGTAA

>GaMYB190 Cotton_A_38532 locus=CA_chr13:79708899:79709459:+

ATGGGGATCGATCCCACCACACACAAGCCCAAATCGAACCATGTCGTCAACCCCACCGGTCGAACCACAGT
GAACCACATGGCTCAATGGGAGAGTGCTAGGCTCGAAGCAGAAGCCAGGCTCGTCAAAGACTCAAAAAAA
CTACCCTCATCTTCTTCAAGACCTTCCCCATATCAGAAAAGTTGTAACAAAGGCTCAAAATCCCAGTGTCT
TGACGTTGTTAAAGCATGGCAAAGCGTAGTGGCTGGTATGTTCGCCACCTCTACTAACAACTCAAACCGCA
TCATATTCGGACCAGACCAGAGCTCCGGAAATTACGAGCTTGATTCAATTATACCTCTTGGAGGTAATGTT
GAAGACGAGTTAATGGTAGGCAACGATAGATCAAAGTGCCAGGTACCAGAATTGAATGAAAGGTTTGATA
ATTACATGTCTTTGCATGATACGACGCATCTTTGGGCTGCTCCCATAGCTGAAAACGACGTCGTAGAAAGC
TTTCCAGATTTCTTGGTGCATGATTTTGATTACCAAATTGACAACGAGGAGTCTATAACCATTTAA

>GaMYB188 Cotton_A_40699 locus=CA_chr13:38345977:38347110:-

ATGGGGCGGCGGCTGCCGTTGAATGGCCATAAGGAGGTGGGTTTGAAGAGAGGGCCGTGGACGGCTGAG
GAAGACCAAATACTAATGGCTTACATTCAACAGCATGGCCATGGTAACTGGTGTGCCTTGCCTGAGAAAG
CAGGACTTAAACGATGTGGAAAGAGCTGTCGACTGAGGTGGATTAATTACGTAAGGCCTGATATCAAAAG
AGGAAAGTTTAGCTTACAAGAAGAACACACCATTATTCAACTCCATGCCCTTCTTGGAAACAGGTGGTCA
GCAATGGCAGCTCACTTGCCAAAAAGAACAGACAATGAGATCAAGAACTATTGGAATACGCATCTCAAGA
AAAGGCTGATAAAGCTTGGCGTTGATCCCATGACTCACAAGCCTCGTACAGATGCCGCAAGCTTCCCATCT
GGTTCAAACTTAACCCACATGGCTCAGTGGGAGAGTGCTCGGCTGGAGGCTGAAGCTAGGTTGGTCCGTG
GCTCCAAACAGGTCGTCCCAAACCCCATCCACCCAAAGAACCACCTTACCCTCAATCATAGTCAGCTCAGG

CCCAGGTGCCTTGACGTACTCAAAGCATGGCAAGGCGTAGTTGCAGGCATGTTTGCCTTCCCCACCCAGGA
CCTTGGGTCCCCAACTTCAACGCTTAGGCTCCCTGCCATTGGACTGAATGCCACTAGCTACACAGATTGTG
AAATGGGGTTTGATGACAGTTTGAAATACATTGAAAACTCGAACCAAATGAAAGAAATTGAGGAAACAA
TAACGGATGGCTGCATTGATGAATGGTTTGAGGACTCATTTAGGGTAGGAAATTATGAAAGTGTACCCAT
GGCTGTCGACGGTAGTAATTGCTGCGGGAGTGTATTCGATTTGGTGAATTCTTCACCATATGGTTTGTCTA
TGTTATATGATCATAATGTCATGAAATGA

>GaMYB140 Cotton_A_04729 locus=CA_chr10:20561870:20563152:-

ATGGGAAGATCACCTTGTTGTGATAAAAATGGGCTCAAAAAAGGGCCATGGACGCCGGAAGAAGATCAGA
AGTTGATTGATTATATTCAAAAACATGGGTATGGAAATTGGAGGACGCTGCCGAAGAATGCCGGTCTTCA
AAGATGTGGAAAGAGTTGCCGTCTTCGATGGACTAATTACTTGAGACCTGATATCAAGAGAGGAAGATTT
TCTTTCGAAGAAGAAGAGACTATAATTCAGCTACACAGTATATTGGGCAATAAGTGGTCGGCTATTGCTGC
TCGGTTGCCTGGAAGAACAGACAATGAAATAAAAAACTATTGGAACACACACATTAGAAAGAGGCTTCTT
CGCATGGGAATAGATCCGGTGACTCATAGTCCTCGGCTGGACCTACTCGACCTATCCTCCATACTAAGCTCA
TCTTTTTACAACCAATCCCAAATGAACATTTCAAGGTTACTGGGTGTGAATTCCTTGGTTAACCCAGAGC
TTCTAAGGCTGGCCACTTCTTTCATGTCATCTCAACGTGAAAACCAAAACCACAAGTTCATCGTTGATCAT
AATGTTGAAGATAACCAGCTTTGCAGCTCCCAAGTCCAGGACCAGTATCAACAACCATTAATGCAGTCTAA
CCATAATCATCTGCCAACGCAAGTCCAAGAAATCCCAGCTTGTTGTATTCCTTTTTCCAATGAAGCAGAGC
TCATGAATCTGGACCAATTCCCATCAAATTTCTCACACTTAAATGACTGGCAAAGCAATGCAATGCCTTCG
AATTTGACGGAGGATTACGTGCCGCTACCGACAAACTATGACTATTACGCCACTGATCATCATCATCATCA
AACCGTGAAGGATCCTTCATCATCCGAAACCTCAAATTTCCAATCCAACAACAGCAACCAGAGTTTCAGC
TTCGCGTCAGTTTTATCAACGCCTTCCTCAAGTCCAACTCAATTGAATTCCAACTCAACATACGTCAACAA
TAGCGGCATTGAAGAAGAACCAGATAGCTACTGCAGCGACATTTTAAAGTTTGAAATCAGAGATAGTTTG
GACGTTAATGATTTCATGTAG

>GaMYB142 Cotton_A_08691 locus=CA_chr10:17404417:17405313:+

ATGCCATCTACTAGGAAAGACTTGGATCGGATCAAAGGTCCATGGAGTCCGGAAGAAGACGAGGCTTTGC
AGCGGCTGGTTCATACGTACGGACCCAAGAACTGGTCTTTAATAAGCAAATCAATACCTGGTCGATCAGGA
AAGTCTTGTAGGCTCCGATGGTGCAACCAGCTTTCCCCCGAGGTTCAGCACCGACCCTTCACTCCCGAAGA
AGATGATACCATAATCCAAGCTCATGCCCAATTCGGTAACAAATGGGCTATCATCGCTCGACTCCTCAATG
GTCGTACGGATAACGCGATTAAGAACCACTGGAACTCCACCCTAAAGAGAAAATGTTCTTCCATGACTCA

AGATTTGAACAATGACTCGCCACAACCAATTAAAAGATCAGCCAGTCTTGGTGCTGCTAATAATGCCTCG
GGTCCTTGCTTGAACCTCGGTACCCCTTCAGGATCCGATTTGAGCGACTCGAGTTTACCCGCTGCCTCACC
GTTTTATAAGCTGTTGGCAAGAACCGGTTCCAGTAAGCATGTTGAAACGGCATCATCCACTACCAATCCAC
CTATTATACTCAGCCTCTCGCTGCCCGGATTCGATCCTTGCGAAAACTCGGATTCGGGACCTAGATCCGAC
CCTATTCCTAGCCCCACCCAGGCGCCAGTAACAACAGTAACGGCTCACCCAACTGCCGGTCTCGTAGTGGG
GATGCAAAACGGGGAATTGGGGATGGAGAAGCAATTTTTTAGCAATGAATTTTTGACAGTTATACAAGAG
ATGATTAGAACAGAAGTGAGGAACTACATGTTTGGGATTGAACAAAATGGTCTTTGTTTGCAAACTGAAG
CTATCAGAAATGCTGTTGTTAAACGTATTGGTATTAGCAAGATCGAGTAG

>GaMYB142 Cotton_A_16664 locus=CA_chr10:8108703:8109569:+

ATGGCTTGTACAAGGAAAGATGTTGATAGGATTAAAGGTCCATGGAGTCCAGAAGAAGATGAGGCTTTAA
AGCGTCTGGTTCAAACCTACGGTTCCAGGAACTGGTCTTTGGTAAGTAAATCGATACCGGGTCGATCCGG
AAAGTCTTGTAGGCTACGGTGGTGCAACCAGCTTTCCCCTGAGGTTGAACACCGACCCTTCACTCCCGAG
GAAGACGATACCATAGTCCGAGCCCATACTCGATTCGGTAACAAATGGGCTACCATCGCTCGACTCCTTAA
TGGTCGAACCGATAACGCGATTAAGAACCACTGGAACTCTACGCTCAAGCGAAAATGCTCTTCGATGACC
GATGATTTGAACGACGATTCACCTCAGCCGCTTAAAAGGTCAGCCAGTCTCAATACTGGTAATGGCGGATC
GGGTCTCTACTTGAACCCGAGTAGTCCTTCAGGATCCGATTTGAGTGATTCGAGTTTGCCCGTTGCCTCAC
CGATAACAATAACGGGGTCTTTGGTGCCTTCAACTCAAACGGCATCCTCGGCTACTGATCCTCCTACTTTA
CTCACCCTCTCGTTACCCGGATCGGATACTAGTGAAACCACTGACTTAGGACCCGTATCCCAACCCGGATC
CAACTCTTTCCCAAGTTCTACCTTGGTGGCAGAACCAGCAACTGTTCCGGCTCTGAAATTGCATATGGAG
AAGCAGTTTTTGAACGCTGAGTTATTGGCAGTGATGCAAGAGATGATAAGAAAAGAAGTACGGAAGTACA
TGAGTGGGAGTGAATCCAATGGGCTTTGTTTTCGAACGGAGGCCATTAGAAACGCCGTCGTTAAGCGTAT
CGGGATTAGTAAGATCGAGTGA

>GaMYB094 Cotton_A_26928 locus=CA_chr10:45886374:45887285:-

ATGGATCGGATCAAAGGTCCATGGAGCCCCGAAGAAGATGATTTGCTTCAAAAACTAGTCCAAAAATATG
GACCTAGAAATTGGTCTTTGATCAGCAAATCGATCCCGGGTAGGTCCGGGAAGTCCTGTCGGCTCCGATG
GTGCAACCAACTGTCTCCGCAAGTTGAGCATCGGGCCTTCACCCCCGAGGAAGACGAGACCATCATCAGA
GCTCATGCCCGATTTGGTAACAAGTGGGCCACAATAGCCCGACTCCTCAACGGTCGTACGGACAACGCCAT
TAAAAACCACTGGAACTCAACGCTAAAACGCAAGTGCTTGTCGGTTGGGGAAGAGAGTAATTTCATTATA
CATGGTGGGTATGACGGTAATTTGGGAGGGGAAGGGGAGCAACAACCGTTGAAAAGATCAGTTAGTGCTG

GGCTTTACATGAGCCCGGGGAGCCCATCTGGGTCCGATTTGAGCGATTCCAGCGTCCCCGTTTTATCATCT
TCACACGTGTACAAGCCCATCCCGAGGACCGGCGGAGTCAGCGTTGACGTCAATGTTATGCCATTACCTGG
GGTCGAAGCGGCGGCGTCTTCTTCTAACGATCCACCGACTTCACTGAGTTTGTCTTTACCTGGGGCTGAA
TCGTATGAGTTGTCAGTTTCAACCCTCCCAGTTACCGAGTCTACTCAGACGAGGAACGAAGCAAAAAATG
ATGGAAAGGGAGGCGAGGTGATGGGGTTCAGTGCGGAGTTTATGGCGGTGATGCAAGAAATGATAAGAA
TGGAGGTGAGAGATTACATGGTTCAGATGCAACAGCAAAACGGTGGCGTTTCGAGAGGAGAGGGAATGG
GGATGTGTTTGGATGCGGGGTTTAGGAATGTTTTGTCTATGAGTCGAGTTGGGGTTAATAAGATTGACTA
A

>GaMYB175 Cotton_A_27562 locus=CA_chr10:43496705:43499563:-

ATGAGTCACACAAAACATGAGCGGGAAGATGGAATGCTCTCTAAGGATCAGACAGAATCGCCATATATCG
ATGATGGTAGCTGTGGAGGAGGTGGAGCTGGGGGAGTTGTTCTGAAGAAAGGACCGTGGACATCAGCTG
AAGACGCGATTTTGATTGACTATGTGAAGAAGCATGGTGAAGGCAACTGGAATGCTGTTCAGAAGCACTC
TGGACTATTTCGTTGTGGAAAGAGTTGCCGCTTACGTTGGGCTAATCACCTGAGGCCGAACTTGAAGAAA
GGGGCATTTACTCAAGAAGAAGAACAGCTGATCATTGAACTCCATGCAAAGATGGGAAATAAATGGGCTC
GGATGGCAGCACATTTGCCTGGTCGTACGGATAATGAGATAAAAAATTACTGGAATACCCGGATTAAGAG
ACGCCAACGTGCTGGGTTACCTCTGTATCCTCCTGAAGTGTGCTTGCAAGCACTGCAGGAGAGCCACAGT
ACCAGTGTGGTTAATGGATTGGATAAAGGACCTAATGATATCTTGCAGAACAATAGCTATGAGATACCTGA
TGTCATATTTGACAGTTTAAAGGCCAATCAAAATGTGCTTCCTTATGTTCCTGAGCTTCCTGTTTTATCCG
CTAGCAGCATGCTGATGAAAGGTCTAGGTTCTTCTCAATATTGTGGTTTTATGCAACCAACGATCCATCGC
CAGAAGCGTCTTCGGGAGTCACCAGCCTTTTTTCCTGGTTACACTGGTGCTGTTAAAAACGAATGCCCTT
TGTTTATGCAGTTTCAAGATGATATATCAGACAAGGCTGCTGGATCCTTTGGGCTGTCTTTTCCAATTGA
ACCAGATCCTGCAACAAAGAACTCCCAGCCTTTTGGTGTATTCCCAGGTAGCCATGCCCTTTCAAATGGCA
ATTTCTCTGCTTCAGAGCCCCCTTTGGAGGCTGTGAAGTTGGAGCTCCCTTCACTCCAATATCCAGAAACT
GAATTAGGTAACTGGGGCACATTAACTTGCCCACCACCTTTACTTGAGTCTGTTGATGCTTTTATCCAGTC
ACCACCTCCAACCAGTGGACTGGAGTCCGATAGTCTTTCTCCCCGTAATAGTGGCCTGCTGGATGCTTTAC
TTCATGAGGCAAAAACTCTAAGCAGTGCAAAGAATCATGCATCTGACAAAAGTTCAAATTCATCTACCCC
CGGTGATATAGCTGAAGGTTCAAATTTCAACATTTGTGAGACGGAATGGGAAAAGTGTGGTGAACCTCTT
TCTCCAATGGGTAATTCAGCAACTTCTCTTTTCAGCGAGTGTATCAGTGCAAGTGGCAGTTCATTGGATG
AACAGCCACCTGCTGAAACTGTTACAGAGTCCCATCTGAAATCCGAACCAGCTGACTGTGTTTTGACCCC
AGAGATACAAAAAGAGGCTCCTATTCGGTTGGACAGTAGTCGCCCCGATACTTTACTTGCTTCAAATTGGC
TAGAGCAGGGTTCTGGTTACGATAAGGACCAAGCCATCTTGACCAATGCCATATCAAGCCTTCTTGGCGAT
GATTTGCGTAATGAGTACAAGAATATGGCAGAAGGAACATCTATTTCAAGTCAAGCATGGGGTCTCGATT
CTTGTGCATGGAATAACATGCCTGCTGTCTGTCAAATGTCTGAACTCCCTTGA

>GaMYB093 Cotton_A_34762 locus=CA_chr10:63459245:63460368:+

ATGGGTAGACCACCTTGTTGTGACAAAATCGGTGTGAAAAAAGGTCCCTGGACACCAGAAGAAGATATCA
TACTCGTGTCTTATATTCAAGAACATGGTCCAGGAAATTGGAGATCAGTTCCTACCAATACAGGCTTGCTT
AGATGTAGCAAGAGTTGCAGGCTTCGATGGACTAACTATCTCAGGCCTGGTATTAAACGTGGTAACTTCAC
TGAAGAAGAAGAGAAGACCATTATCCATCTCCAAGCTCTTTTAGGCAATAGATGGGCAGCCATAGCTTCTT
ACCTCCCTCAAAGAACAGATAATGATATAAAAAACTATTGGAACACCCATTTGAAAAAGAAGTTGAAAAA
GGGTCAAAATCAAGATGGGGTTTCTTATTGTCAATCGGTCCCTAAGGGTCAATGGGAAAGAAGGCTTCAA
ACAGATATCCGGATGGCTAAACAAGCACTCTCCGAGGCATTATCTCTTCGGAAACAAAACCCTTCAACTGA
TTCAAACGACTTCAATGATCTCTTAAACTCAGCTCAGCCTCATCAACCACCCACTTACGCTTCCAGTGCCG
ACAACATATCCCGGTTGCTCCAAAACTGGATGAAAAACACACCCAAACCGGCTTCCGCCACCGCCGCCGCG
AACTCGGCCGAAACAATGACCCGGAGTTCCTTCAACTCTAATGATGAAGGGGCATTGAGTGACAAGGGTT
TGGACTCATTCTTCAGCTTCAACTCTAGCAGTACTTGTTCAGATAATGATGTCTCACTTGAATCTGAAAAC
AGCGTCGTTTTCCAAGTGGAAAGTAAGCCCAACATGGGGGATCAAATGCCTCTTACTTTGATAGAGAAAT
GGTTGTTGGATGATGTGCTGTTTTGA

>GaMYB169 Cotton_A_05028 locus=CA_chr3:91178440:91180116:+

ATGGGGAGATCACCTTGCTGTGACAAAGTGGGGTTGAAGAAAGGACCATGGACACCAGAAGAAGATCAG
AAACTCTTAGCTTACATTGAAGAACATGGTCATGGAAGCTGGCGTGCCTTGCCGGCCAAAGCCGGTCTTC
AAAGATGTGGAAAGAGCTGCAGGCTTAGATGGACTAATTATCTCAGACCTGATATTAAGAGAGGAAAGTT
TAGCATGCAAGAAGAGCAAACTATCATTCAACTTCATGCCCTTTTAGGGAACAGGTGGTCGGCTATAGCCA
CTCACTTGCCAAAGCGAACAGATAATGAGATAAAAAACTACTGGAATACTCATCTTAAGAAAAGGTTAGC
CAAATTGGGGATTGATCCGATCACTCACAAGCCAAAATCTGATGCTTTACTCTCCATTGATTCTCAATCCA
AGAGTGCAGCAAACCTTAGCCATATGGCTCAGTGGGAAAGTGCTAGGCTTGAAGCTGAAGCTAGATTGGT
TCGAGAATCAAAGCTACGTTCACATTCACATTCATTTCAACACCGCCTGACTCGTCCACCAACAGCAGCTT
TTGCTTCTTCAGCCGGTGAGCTTGTGAATAAGACAGCTTGGAACAGTACTGCCGGGTGGAGTAAATCCAG
TGAAGTTAATAATGGTGTTGTAAACAATGGATTTGGTGACCTCGAATCTCCCAAATCAACACTTACTTCCT
CAGAAAATGGGGTTGGATTGAGTTCAATGGGTATGCCAGATTTTGTTGGCACTGCATCAGCTTCATCTGA
GATCAAACAAGAAGGTGAACAAGAATGGAAAGGCTTTGGAAGTTCAACTAATTTGGCCATGGAGAATGG
GTTCAATGATATTGGAAACGCAATGGAGGATGGCTTCATTAACCTTCTTCTCAATGATTCCACCGACCCCA
GTTTATCAGATAGTGGCAAAGAATCTGATGGAAACAGCGGTGATGGTATTGCCAGAGATGATCACTATGA

AGATAACAAGAATTATTGGAACAGCATTCTTGATTTGGTGAATTCTTCCCCATCGGATTCACCAATGTTCTAA

>GaMYB166 Cotton_A_03861 locus=CA_chr9:61362712:61364226:+

ATGGGAAGAGCACCTTGCTGTGATAAAAATGGTCTGAAGAAAGGTCCATGGACACCTGAAGAAGATCAGAAACTTATCGATTATATTCAAACACATGGTTATGGTAATTGGAGAACATTGCCCAAGAATGCTGGGTTACAAAGGTGTGGAAAGAGTTGTCGTCTTCGTTGGACTAATTATTTAAGACCTGACATAAAACGAGGCAGGTTTTCATTTGAAGAAGAAGAAGCCATAATTCAACTCCATAGTGTGTTGGGCAACAAGTGGTCTGCCATAGCTGCACGGTTGCCTGGAAGGACAGATAATGAAATAAAGAACTATTGGAACACACATATTAGGAAGAGGTTGCTTCGAATGGGGATTGATCCAGTGACACACAGTCCACGATTGGATCTTCTTGATTTGTCTTCAATCTTAGGTTGTTGTTCTTTTTATAACCAGTCTCAAATGAACATGTCGACGAGGTTGCTCGGCGGCGGCGGTGGTGTTCAACCCTTGGTGAACCCTGAGATTCTAAGGTTAGCCACGTCTATCATGTCATCACCTCAACGTGAAAACCAAAACCCAGATTTTCTTTTCCATGAAAATCAATACCAGCAACCGCCATTAATGCAAAACACAAATGTAGCAGAACCGGTGATGAACAACAACCCTAATGTAATAATAGATCAGTTTCCATTTAATGGCTGTTCCACTGACTGGCAAAACAATGCCAATGACGTACTCCCATATTTGACCGAAGATAATTACGTTCCGGTACCGTCAAACTGTTACGGCGGCGGAAACGGAGAACCGAGCTTCAGGACGCCGTCTTCATCGAGTCCAACGCCGTTGAATTCAAATAATTCAACGTACATCAACAGTAGCAGCACTGAAGATGAAAGCTATAGCAGTGATATATTGAAGTTTGAAATCCCAGATTTTTTGGATGTTAATGAATTCATGTAA

>GaMYB172 Cotton_A_26728 locus=CA_chr9:60364674:60366041:+

ATGGATGTTCAGGGAAGGGATTGTGTCCCAAAAGCTCAAAGTAGTGAGGAGGATCAGATGGAGTTGAGAAGAGGTCCTTGGACTGTGGAAGAAGACTTCAAGCTCATCGATTACATTGCCACACATGGCGAAGGTCGATGGAATTCTCTTGCTCGTTGTGCAGGTCTCAAAAGGACGGGCAAAAGCTGTAGATTAAGATGGTTGAATTATCTCCGACCTGATGTTCGACGTGGGAACATTACTCTTGAGGAACAACTTTTGATTCTTGAGCTTCATTCACGCTGGGGAAACAGATGGTCCAAAATCGCCCAACATTTGCCTGGAAGAACCGACAATGAGATCAAGAACTACTGGAGAACCCGTGTCCAAAAACATGCCAAGCAGCTTAAGTGCGACGTCAACAGCAAGCAATTCAAAGACACCATGCGTTACCTTTGGATGCCTAGGTTAGTGGAAAGAATCCAAGCTGCCAACGCCGCCTCATCCACCTCCACCGCCGCCGTTACTACCTCCGTCGTGGGTACGGAACCAATGGTGTTTCCCGATGACCACCACCTTGGGGGCGGCGCACAACAAGTGACCTCCTCAAGTAATAATAATTACACCCTAGAGAATTCCAGCACAACTGCAGCCTCATCGGACTCTTTCGGGACACAAGTTTCACCGGTTTCGGATTTCGCTGATTATTACAGTAACATCTCCATTAATCATAACCCTAATCCAAACTGCTTCGAAGCTGGTAATTACCACAACAACGGCTTAGATTTTCAGTGT

TTGGAGCAAAACAACCCCTGGCTGGATACCGTGGATGGTTCAGACAATATCTTCGACGCTGAGGATCTTT
ACTTCTTACAGCAGCAATTCAATTTTAACATGTGA

>GaMYB189 Cotton_A_40365 locus=CA_chr9:26797762:26798928:-

ATGGGACGGTCTCCATGCTGTGATAAGGTGGGGTTGAAGAAAGGGCCATGGACTCCCGAAGAAGATCAAA
AGCTCTTGGCTTACATTGAGCAACATGGCCATGGAAGCTGGCGTGCTTTGCCTGCCAAAGCTGGGCTTCA
AAGATGTGGCAAGAGTTGCAGACTTAGGTGGATTAACTACTTAAGACCTGATATCAAAAGAGGAAAATTC
AGTTTACAAGAAGAACAGACCATCATTCAACTCCACGCCCTTCTCGGAAACAGGTGGTCGGGTATTGCAG
CTCATTTACCAAAAAGAACAGACAATGAGATTAAGAACTACTGGAATACACATCTAAAGAAAAGATTGAA
CAAGATGGGGATCGATCCCGTGACGCACAAGCCTAAAACTAATGCCCTCGGGTCCGCAACCGGTAACCCCA
AGGATGCAGCTACCCTCAGCCATATGGCTCAATGGGAGAGTGCTCGGTTAGAAGCCGAAGCCAGGCTGGT
AAGAGAGTCAAAATTCGTTGGCTCCTCCTCTTCCTCTTCTTCTTCTTCTTCTTCCTCGGCTCCACAGCACA
CTAGCAACACTGTCATGACTCCGCCGGCGACTAGGCCACAATGCCTCGATGTGCTCAAAGCTTGGCAAGGT
TTAGTCACCAGATTGTTCACTTTCAATAACACAACTGACAACCTCCAGTCTCCGACGTCAACGTTGAACTT
CGTGGAGAACACCAACACGTTGGCTAATGGATTAATCAATGAAAACTCCATGGAGTTGCATGAAATGGGT
GCATGGTTTCGTCAAGATTCTTCATACAGGGCAGTAGAGAACATGGACATGGAAGATTATTCGGATATGAT
GGTCTGGGAATCTGGGGATCATCAGCAGTGGTCATCAATGGCGGCGCCAGCTGAAAACTTGAACGAAACA
AGCTATGGTAATAGTAGTAGTAGTAGTAGTAGTAGTTTAGAGGAGAATAGGAATTACTGGAATAACATCCTTAA
TTTGGTAAGTTAA

>GaMYB131 Cotton_A_01409 locus=CA_chr6:120239220:120240546:-

ATGTCACCATCATCATCATCGTCATCATCGTCTCTTAAAAAAACAAGCACAAGTTGCAGTGAAGATGAAG
GTGATCATCATCAACAGCAGCTGAGGAGAGGACCTTGGACTATAGAGGAAGATTCAGTCCTTGTTCATTA
CATTGCTCGACATGGCGAAGGTCGATGGAATTTTCTTGCAAAACATGCAGGTTTAAGGAGAACAGGCAAG
AGTTGCAGGTTGAGATGGTTGAATTATTTGAAACCAGATGTTAAGCGTGGGAACCTTACTGCTGAAGAAC
AGTTTTTGATCCTTGAACTCCATTCTAAATTGGGTAACAGGTGGTCCAAGATTGCACAGCATCTGCCTGG
AAGAACCGATAATGAGATCAAGAATTACTGGAGAACCCGTGTGCAAAAACAGGCTCGGCAGCTTAACATC
GATGCTAACAGCGCGGCGTTTAAGAACATTCTCCGATATTATTGGATGCCAAGATTGGTTCAAAAGATGG
AAGAGTCGACGTCGCCCTGTTTTTCATCGACGGTTATTCCTCGGAACCAACCGTTAACGAAGACCGATTAC
GTTGACGGGAATTCATGTGTCTCTTCATTACAACATGTGGATAATATGAAAATGTCCCAATTTGAGATATT
TGGTAACAATGATTGCAATAGTAATGCTCTTGCAAAGGATTGGTATGGTTATGTTGGTGATAATGGTAGCT

ATTGCCATGGCATGGAAACCATCAACATGGCGTCCACGTCAGCACTGGTCGCGGAGGGATTTCCGACCCCC
GCCGGGGATTGCCACTTGGCGGATGATAATTGGGTTAATGATGGTTTTTAGATGGGATATGGAGCATGG
GGGAACTATGGGAACTTAGAAATGCATTGCATTGA

>GaMYB127 Cotton_A_06162 locus=CA_chr6:68332194:68333356:+

ATGCGGCCACCGTCTCCGAACAGAAAGAAAGAGGTGAGATTGAAGAGAGGGCCATGGACAGCTGAAGAA
GACAAATTACTGACGGCTTACATTCAAAAACATGGCTATGGCAGCTGGGGTTCCTTGCCTCACAAAGCTG
GACTTGAACGATGTGGAAAGAGCTGCCGACTGAGATGGATTAACTACTTAAGACCTGATATCAAAAGAGG
AAAGTTTAGTTTAGAGGAAGAACAGACCATCATTCAACTCCATGCCTTTCTTGGAAACAGGTGGTCGGCA
ATAGCGGCACACTTGCCTAAGAGAACAGACAATGAGATCAAGAATCACTGGAACACACATCTAAAGAAAA
GGCTAATCAAAATGGGTATTGATCCCATGACTCACAAGCCCTCAACCTCCCCATCACCCAAAAATGGTTCA
AATTTAAGCCACATGGCCCAGTGGGAGAGTGCACGTCTACAGGCTGAAGCCAGGTTGGTCCGTGAGTCAA
AACAGGTTGTCCCAAATCTTACCACCCGCCCCACTAGGAGGAGTCAACTCACCAGAAGCAGTCCCAGGTGC
CTTGACGTACTCAAAGCCTGGCAAGGTGTAGTTGCCGGGATGTTTGTTTTCTCCACCCAGGATCCCAGGT
CCCTAACAACCTCAACTCTTCGTTTCCCTTCAGCTGGATGGGGAGAAGCTGAGGAATGGCGGGGCCAGGG
AATGAAGGGTTCCAGCGATGCTGATGATGCATGGTTTGAGGAGGACTCAGTCATACTACACAGTCTACCTA
TTGAAAATATAATGGAAGGTTTGTCGGATGCTTTTATTTTGAATTCATGGATGGGTGTCGACGAATCAAC
AGATGAAAACACTGTAAAGGAGAATGGTAATTGCTGGGATAGCGTACTCAACTTGTTGAGTTCTACACCA
TGTGGTTCGCCTGTGTTGGGATAA

>GaMYB126 Cotton_A_06163 locus=CA_chr6:68367472:68368994:+

ATGCAGCAGTCTCCATGTAGCGACAAGGTGGGGTTGAAGAAAGGGCCATGGACTCCAGAAGAAGACCAAA
AACTCTTGTCTTATATTCAAGAACACGGCGGTGGAAGCTGGCGAGGCTTGCCCGCAAAAGCTGGACTTCA
AAGATGTGGCAAGAGTTGTAGACTTAGGTGGATTAACTACTTAAGACCAGATATCAAAAGAGGAAAGTTC
AGTTCGCAGGAAGAACGAACCATCATTCAACTCCATGCCCTTCTTGGAAACAGGTGGTCGGCTATTGCGG
CTCATTTGCCAAAAAGAACAGACAATGAGATCAAGAACTACTGGAATACACAGTTGAAGAAAAGGTTGAC
GACGATAGGGATCGACCCTGCAACTCACAGGCCTAAAACCGATACCCTCGGTTCAACTCCCAAGGATGCCG
CTAACCTTAGCCACATGGCTCAATGGGAGAGTGCTCGGTTAGAAGCTGAAGCTAGATTGGTGAGAGAGTC
GAAACGAGTTTCAAACCCTCCGCAAAACCAATTTAGGTTCACGTCTTCATCGGCTCCTCCACTGGTAAGC
AAAATTGATGTTGGTTTGGCTCATGCTACTAAACCGCAATGCCTCGATGTACTCAAAGCTTGGCAACGTGT
AGTCACTGGATTGTTCACTTTCAACACTGACAACCTCCAATCTCCAACATCGACGTCGAGCTTCACGGAA

AACACGTTACCAATCTCATCTGTCGGGTTCATTGACAGCTTTGTGGGGAACTCAAATAACAGCTGTTGCG
GAAATAATTGGGAATGTGTGGAGAAATCGAGCCAAGTTGCTGACTTACAGGAAAGATTGGATAACTCAAT
GGGGTTGCATGACATATTGGATCTCTCCTCAGAAGATGTATGGTTTCAAGGCTCATACAGGGCGGAAAATA
TGATGGAAGGGTATTCGGACACGTTAATGGTTTGTGATTCTGGGGATCATCCGAAGAGTTTGTCAATGGA
GCCTAGACAAAACTTTAATGTTGGAACAAGTAATGCTAGTAGTTTCGAAGAAAACAAGAATTACTGGAAC
AACATCCTTAATTTTGCGAATGCTTCCCCTTCTGGTTCTTCTGTCTTTTGA

>GaMYB109 Cotton_A_14816 locus=CA_chr6:87863684:87866091:+

ATGATGATGATGATGATGGGAGGGAACAACCAATTTACTACACAAAACGAGGGTGGTGAGCCCTTAGGTA
ACAATGGGATGAACAACAGTGGTGTCTGTAGTGGAAGAGAAAGCGGGATACCGTTGAAAAAGGGGCCGT
GGACGGCGATAGAGGATGCGGTGTTGGCAGAATACGTGAGGAGTCATGGTGAAGGGAGTTGGAATGCTGT
GCAAAAGAACACAGGGTTGGCACGTTGTGGGAAGAGTTGTAGGCTTAGATGGGTTAACCATTTGAGACCA
AACTTGAAGAAAGGATCTTTTTCTCCTGAAGAAGAAAAGATTATTATTGAGTTGCATGCTAAGAAGGGGA
ATAAATGGGCTCGTATGGCTATTCAGCTTCCTGGAAGAACAGACAATGAAATCAAGAACTATTGGAACAC
AAGGGTGAAGAGAAGGCAACGCCAAGGCCTCCCACTTTACCCACCTAACGTCCAACCCTTCTATTCCCAGC
ACCAGCACCAGCACCAGCACCGCCAGCGTCACTCACATCCTCCCTCACCTATTCCTTCTCCTCCGCCAACCA
CCGCACCCAACTCCTGTTTCTCCTTCCAAACTCCCGTAGTGTCTCTTCACACCTCAAATCCCATGCCTCTT
CATCCTCTCCACATCCCTCACAGACCACCACCTCAAAACTTCCTTTACAATCCTCATTCAGCCTTAACAAC
GCCGCCTCTGCCACTCCAAAGCCCGAATTCAGCCTCGACGCCGCCGCCTCTCCCTAGCCCTTCCGCTTCAA
CACCACCCCATATCTCCCCACTTCACTCTCCCCATAACCCACCTCCTTTCCCAACTCTCCCTCTAATCAATT
ATCCCAATACCACCACCGACGACGACTTCTTCCATAGCAACAAGCGGTTCAAGCATGACAGACTTCAAAGC
AACAATTACAATAACAATCATCTTGATGCTACAAGCTCGAGCTTTACATTGCCGTTTTCACCAATGCAGCA
CTATTCGAATCGAATGACACTCGATCTTCCTTCATCGTCGAGGACAACATTTAATCATCATCCTCATCAGG
ATTCTGGGGATTTTTACCCGTTGAAAATGGATCTCCGTTCGAACCAAATCAACGACGATGGGTTGTTGGA
GACAATGCTGCAGGAAGCTCAGGCACTAGCCGCCAATGGCAGCGGTGGCAATAATGAAATACCAAAAGAG
ACGATGAACGCTGCACAAAATGAAGAAGACTATTCGAGGCTAATCAACATTGATGGTCCTTCTTCCTTTA
GCATCGCGATCCCCGAATGGTGCAACGACAGTGGCGAAAGCTCCGAGCGGCAGCCCTTTGCTATAATAGAT
AATGAAAATCATTTAGCCCCTGATAACCATCAGATAGCTTCATTGTACCCAGCTGATATTTCGCCCCACCA
TGCTGCCAGGTCTTCGAGCGTTCGATCCTGGGACAACTTTCCGGTACTAGAACAATCAAGATTTTTAGTG
AAGATATAA

>GaMYB110 Cotton_A_14822 locus=CA_chr6:87897424:87900237:+

ATGATGATGATGATGATGATGGGAGGGAACAACCAATTTACTGCACAAAACGAGGGTGGTGGGTCCTTAA
GTAACAATGGGATGAACAACGGCGGTGTTGGTGTCGGAAGAGAAGGTGAGATAGTGTTGAAAAAGGGGC
CATGGACAGCGGCGGAGGATGCGGTGTTGGCGGGGTACGTGAGGAGGCATGGTGAAGGGAATTGGAATG
CTGTGCGAAAGAACACAGGGTTGGCACGTTGTGGGAAGAGTTGTAGGCTTAGATGGGCTAACCATTTAAG
ACCAAACTTGAAGAAGGGATCTTTTTCTTCGGAAGAAGAAAGGATTATTATTGAGTTGCATGCTAAGATG
GGGAATAGATGGGCTCGTATGGCTACTCAGCTTCCAGGAAGAACAGACAATGAAATCAAGAACTATTGGA
ACACAAGGGTGAAGAGAAGGCAACGCCAAGGCCTCCCACTTTACCCACCCCACGTCCAACCCCATTATTCC
CAGCACCGCCAGCGCCACTCACATCCTCCGTCACCCATCCCTTCTCCTCCGCCAACCACCGAACCCAACTCC
TGTTTCTCCTTCCAAACTCCCGTTGTGTCTCCCCACACCTCAAACCCCATGCCTCTTCACTCTCTCCACAT
CCCTCACAGACCACCACCTCAAAACTTCCTTTACAATCCTCATTCAGCCTTAACAACGCCGCCTCCGCCAC
TCCAAAGCCCGAATTCAGCCTTGGCGCCTCCACCTCTCCCTAGCCCGAACGCTTTAACACCACCCCATACC
TCCCCACTTTACTCTCCCCATAACCCATCTCCTTTCCCAACTCTCCCTCTCTTCGATTATCCCAACATCACC
ACCGACGACGACTTCTTCCATAGCAACAAGCGGTTCAAGCATGACGGACTTCAAAGCAACAACTACAATA
ACATTCATCTTGATGCTGCCAGCTCGAGCTTTACGTTGCCGTTTTCACCAATGCAGCACTATTCGAATGGA
ATGACACTCGATCTTCCTTCATCATCGAGGACAGCATTTAACCATCATCCTCATCAGGATTCTGGGTGTTT
TTACCCGCTGAAAACGGATCTCCGTTCGAACCAAATCAACGACGATGGACTGTTGGAGAATATACTGCAG
GAAGCTCGGGCGCTGGCCGCCAATGGCCGCGGTGGCAACAAAGAAATGCCGAAAGAGATGATGAACTCTG
CACAAAATGAAGAAGAAGACTATTCGAGGCTAATCAACATTGATGGTCTTTCTTCCTTGGGCATGGCGAT
CCCCGAATGGTGCAACGACAGTGGCGAAAGCTCCGAGCGGCAACCCTCCGTTATAACAGATAATGAAAAT
CATTTAGCCCTTGATATGCATCAGTTAGCTTCATTGTACCCTGCTGATATTTCGCCCAACCATGCTGCCAG
GTCTTCGAGCGTTCGATCTTGGGACAAGTTTCCGGGATTGTGCTGA

>GaMYB125 Cotton_A_23032 locus=CA_chr6:20629952:20630774:+

ATGTCCACTCTTATTGAAGAGGAGATTGAGGTGAGAAAAGGGCCATGGACCACGGAAGAGGATACGCTCC
TAACCCATTACATTGGTCGTCATGGGGTAGGTCCTTGGAATATGTTGGCAAAATGTGCAGGTCTTAAGAG
AAGTGGTAAAAGTTGCAGATTAAGGTGGCTTAACTATTTGAACCCTGACATTAAGCGCGGAAACCTCACT
CTCCAAGAACAACAATTGATTCTTCAACTTCATTCCCTGTGGGGAAATCGATGGTCAAAAATTGCAGAGC
ATCTTCCGGGAAGAACAGACAACGAGATCAAGAACTATTGGAGAACAAGGGTGCAAAACAAGCAGCCATC
CTCTTCTTCTTTGAAAGAAATGAGCTCCCAATTTTCAGTTCCTTGTCAATTACCAGAATGCATCGTCCCTT
CAGGTTCAGTCATAAACATTTCAGAGCAAATTGAATTTCCCCAACACGAAACAAGTCCAAACGCATATGC
CCATACTTGTGTCGACAACAATAACCTAGTTCTTAATGGAAGTTACAATATTGGCAGAAGTGGGCAGGACA

TGGAGGCATTCAGACTGGCATCCATGTCAGCTGTGGGTGAAGGGTGTTGGATATGTAATGAGATGACAGA
TAGTTTATGGCAATGTAGGGAGTTGGGAGAGATGGGGAACTAG

>GaMYB176 Cotton_A_35093 locus=CA_chr6:3749539:3751292:+

ATGGGGAGATCACCTTGCTGTGACAAAGTAGGGTTGAAGAAAGGACCTTGGACACCTGAAGAAGACCAG
AAACTCTTGGCTTACATTGAAGAACATGGTCGTGGAAGCTGGCGTTCCTTGCCCGCCAAAGCCGGTCTTC
AAAGATGTGGGAAAAGTTGCAGGCTCAGATGGACTAATTATCTAAGGCCTGATATTAAGAGAGGAAAATT
TAGTTTGCAAGAAGAGCAAACTATCATCCAACTTCATGCCCTTTTAGGGAACAGGTGGTCTGCGATTGCC
ACTCACTTGCCAAAAAGAACAGATAATGAGATAAAAAATTACTGGAACACTCATCTTAAGAAAAGGTTAG
CTAAAATGGGGATCGATCCCATCACTCACAAACCCAAAAACGATGCTTTACTCTCCACTACTGATGGTCAA
TCCAAGAATGCAGCCAACCTTAGCCATATGGCTCAATGGGAGAGTGCTAGACTCGAAGCTGAAGCTCGAC
TGGTTCGAGAATCAAAGATCCGTTCACATTCAGTTCAACATCATCACCACTTCAATCCCCCTGCTTTTACC
CTTGAGTCTCCTACATCCACTCTTTCTGTCTCAGAAAATGCACCACCAATCATTACAGGGCTCGGTGTGAG
TCCGATGCCGATGATCGAGTTCGTCGGCACCACCTCGGGTTCCTCCGAGACGGCGGGGATCGTTAAAGAA
GAAGGTGAACAAGAGTGGAAAGAGCTTGGAAGTTCAAGCAATTTGGCTGATTACAAAGAGGGTATGGGG
AATTCTTTGTCATCATTCACATCTAGTCTTCAGGATATGACAATTTCTATTGAAGGAGGATGGACTCCAGA
GTCTTTGAGGCCAAACAACATTAATGTTAATAATGTTGGGAATATAATGGAGGAAGGTTTCACTAATCTT
CTTCTTAATGATTCCGTCGACCGGAGTTTATCGGATAGCGGCAAAGAATCTGACGAAAACAGCGGAGGCA
GCGGGGATGGCAGTGATTACTATGAAGATAACAAGAACTATTGGAACAGTATTCTTAATTTGGTGAATTC
TTCCCCATCAGATTCACCAATGTTCTAA

>GaMYB121 Cotton_A_39138 locus=CA_chr6:61766975:61767900:+

ATGGAAGTTTACGAGAGAGGTTTCTTCTCCATATCTGAAAGTGGTCACCAAATTGAAGAAGAGATGGACG
TTAGGAAAGGTCAGTGGACTGAAGAAGAAGACTCCATGCTCAAGGCTTATGTCACTCTTCACGGTGAAGG
TCACTGGAATTCCGTCGCTCGTTTCTCAGGGTTAAGAAGAACAGGGAAAAGTTGCAGATTGAGATGGCTA
AATTACTTGAGACCTGCTATTAGACGAGGCAGCATCACCCTTGAAGAACAATTATTGATTATTCAACTCCA
TTCTGTTTGGGGCAACAGGTGGTCGAAGATTGCCGAACACTTGCCTGGAAGGACAGACAATGAAATAAAG
AACTACTGGAGAACCAAAGTCCAGAAACAGGCAAAGAAGCTTGAATGCGACGTTAACAGCCGCAAATTCA
GGGACGCCTTGCGCTACGAGTATATCCCTCGTTTAATAGAACAGATCAGCCGTGCTAAACAGGAGTCACCC
TCTGGTCAACTCACCTATGGAAACGAAACTGAGTCGGTCCAGGTCGACCAGAGTCTCCTACCTGAGTCTTC
GTCGGAAATCCAAGTCACATACGGTTCAACTGTAGCAGAGACATGGGGTATAGATGAGCAGAGCAATAGC

AATGGTTGGCTTGGTGGTGGAGATTACTGGATGGAGAGTGAGTGGTGTGAAGAGAATATTTGGTTCTTAC
AACAGCAGCTTTATGATGATGAGGATAATGAGATCTAA

>GaMYB0124 Cotton_A_17954 locus=CA_chr5:17498490:17499692:-

ATGGGGAGAACACCTTGCTGTGACAAAAGTGGCCTCAAGAAAGGTCCCTGGACTCCCGAGGAAGATCTTA
AGCTTACTAACTATATTCAGGTTCATGGACCAGGAAACTGGCGTACTCTCCCTAAGAATGCCGGTCTCCAA
AGATGTGGGAAGAGTTGCCGTCTCAGATGGACGAACTACCTTAGACCTGATATCAGGAGAGGAAGGTTCT
CATTTGAAGAAGAAGAAACTATAATTCAACTACACGGTATCTTGGGGAACAAGTGGTCGGCTATTGCGGG
TCAATTGCCAGGAAGGACCGATAATGAAATTAAGAATTACTGGAACACCCATATCAGGAAAAGGCTTCTA
AGGAATGGGATCGATCCAGTGACACATGCTCCACGTCTTGATTTGCTTGACCTTTCTTCCATTATCAGCTC
AACTTTGTGCAACCAATCTCTTCTCAATGTGTCAAACTTGTTAGGTACACAAGCTCTCCTAAATCCCCAAC
TGCTCAGCCTAGCAAACACCCTCTTGTCTCTAAAACAAGAAAACCCAGAAATGCTGATGCAATATCTCCAA
CAAAACCAACTAAATCTAGACCCCTCGTTACAAGCTCCCATTCAAACTGCTTCAGCTTGCACCACCACATC
CACCGTCCCTTGTTGTACTTCGATGCTAAGCCAAACAGGCCTCATACAAGCCTCCGGAGAAGGCTTTTTCT
CTTCCGATATGACGAACTTCAGCTACCCAAATTCCCAAGAAATTTTGACAACTTCCACTTTAACCAATGAA
TTTGCTGCGCAACCACATTACGTGCAGTGCAGCACAAATCCTACAGTTCCCATTCTGTCTGAAACTTCCAA
TTTCCAATCAGTGGATGGCAGCTTTGATTCGGTGAGGTCGTCGGCGATATCAAGTCCAACGCCTTTGAAC
TCATCATCTACATTCGTGAACAGCAGCAGTACCGATGACGAGAGAGAAAGCTTTAGCAGCTTGCTTAAGT
TTGAAATACCGGAGGGCTTGGATATCAATGATTTTATGTAA
